# Neural mechanisms of brand love relationship dynamics: Is the development of brand love relationships the same as that of interpersonal romantic love relationships?

**DOI:** 10.3389/fnins.2022.984647

**Published:** 2022-11-10

**Authors:** Shinya Watanuki

**Affiliations:** Department of Marketing, Faculty of Commerce, University of Marketing and Distribution Sciences, Kobe, Japan

**Keywords:** consumer neuroscience, neuromarketing, brand equity, brand relationship management, consumer psychology

## Abstract

Brand love is a relationship between brands and consumers. Managing the relationship is an important issue for marketing strategy since it changes according to temporal flow. Brand love theories, including their dynamics, have been developed based on interpersonal romantic love theories. Although many brand love studies have provided useful findings, the neural mechanism of brand love remains unclear. Especially, its dynamics have not been considered from a neuroscience perspective. The present study addressed the commonalities and differentiations of activated brain regions between brand love and interpersonal romantic love relationships using a quantitative neuroimaging meta-analytic approach, from the view of brain connectivity. Regarding the mental processes of each love relationship related to these activated brain regions, decoding analysis was conducted using the NeuroQuery platform to prevent reverse inference. The results revealed that different neural mechanisms and mental processes were distinctively involved in the dynamics of each love relationship, although the anterior insula overlapped across all stages and the reinforcement learning system was driven between both love relationships in the early stage. Remarkably, regarding the distinctive mental processes, although prosocial aspects were involved in the mental processes of interpersonal romantic love relationships across all stages, they were not involved in the mental processes of brand love relationships. Conclusively, although common brain regions and mental processes between both love relationships were observed, neural mechanisms and mental processes in brand love relationship dynamics might be innately different from those in the interpersonal romantic love relationship dynamics. As this finding indicates essential distinctiveness between both these relationships, theories concerning interpersonal romantic love should be applied cautiously when investigating brand love relationship dynamics.

## Introduction

Brand love is one of the most crucial concepts in marketing and consumer psychology (Batra et al., 2012). Studies on brand love have been conducted based on interpersonal romantic love studies (Shimp and Madden, 1988; Ahuvia, 1994, 2005a,2005b; Fournier, 1998; Thomson et al., 2005; Carroll and Ahuvia, 2006; Albert et al., 2008, 2009, 2013; Park et al., 2008; Albert and Valette-Florence, 2010; Lastovicka and Sirianni, 2011; Sarkar, 2011; Ahuvia et al., 2014; Langner et al., 2015; Bagozzi et al., 2017). Although the definition of brand love is controversial, major interpretations have converged. Shimp and Madden (1988) clustered brand love relationships into eight types (i.e., “non-liking,” “liking,” “infatuation,” “functionalism,” “inhibited desire,” “utilitarianism,” “succumbed desire,” and “loyalty”), based on Sternberg’s love theory (Sternberg, 1986). These types were decided based on a combination of three psychological components (i.e., “liking,” “yearning,” and “decision/commitment”). For example, the infatuation type of brand love consists of the “yeaning” component. The utilitarianism type of brand love consists of both the “liking” and “decision/commitment” components. Thomson et al. (2005) organized constructs of emotional bonds between consumers and brands by applying Bowlby’s attachment theory (Bowlby, 1969). Batra et al. (2012) proposed comprehensive brand love constructs that are composed of multiple factors including constructs such as passion, integration of the brand with self, emotion, trust, and rational evaluation. Moreover, the state of loving brands is a predictor of brand loyalty (Thomson et al., 2005; Batra et al., 2012). Like interpersonal romantic love relationships, brand love relationships also develop dynamically. Although brand love relationships are basically unidirectional from a consumer’s perspective, brand managers can generate and develop a quasi-interactive relationship by stimulating consumers’ perception *via* marketing-based communication such as advertisements and direct mails. The present study interprets brand love in a broad sense and defines it as a phenomenon or mental state generated by subjective emotional feelings for objects formed by the relationships between the consumers and objects in a consumption context.

**FIGURE 1:**
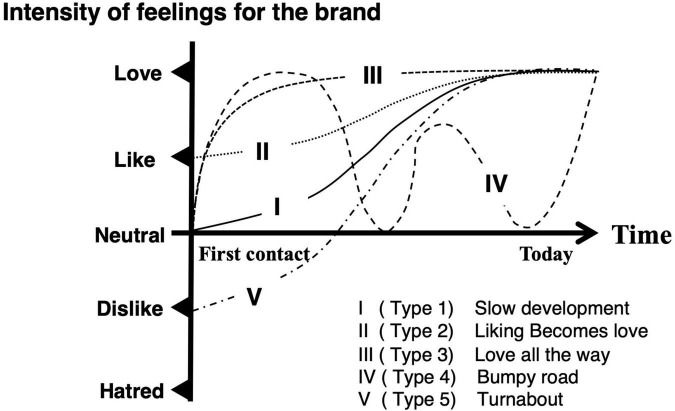
Brand love trajectories as defined by Langner et al. (2016) were modified and reorganized by the present author. Reprinted with permission from Springer Nature (License 5423700948437).

Shimp and Madden (1988) pointed out that the dynamics of the relationship between brands and consumers could be described as a logistic or an S-curve function. In the early stage of the brand love relationship, the more a consumer gets acquainted with a brand, the more he/she becomes familiar with it. Their relationship becomes stronger and the slope of the function gets steeper. Intensive emotions toward a brand in consumer perception contribute to building their relationship in this stage. In the mature stage, their relationship becomes moderate by accumulating experiences. Finally, the slope of the function becomes flat. Although they did not qualitatively or quantitatively validate the dynamics of the brand love relationship, a few studies precisely investigated its dynamics. Langner et al. (2016) first investigated the trajectories of brand love relationships. They conducted an interview survey regarding temporal trajectories of brand love. They classified its trajectories into five types (i.e., Type 1, “Slow development;” Type 2, “Liking becomes love; “Type 3, “Love all the way;” Type 4, “Bumpy road;” and Type 5, “Turnabout”), as described in Figure 1. In all the trajectories of developing brand love relationships, intensities of the feelings toward a brand surged during the early stage of the relationship and calmed down over time. Gumparthi et al. (2021) conducted further research on the triggering factors of turning point in trajectories by means of an approach same as that of Langner et al. (2016). They also classified the developmental trajectories of brand love relationships into five types (i.e., “Turnaround to Love,” “Drop in Love,” “Gradual Development,” “Liking to Love,” and “Roller Coaster Ride”). The trajectory patterns that they identified were almost similar to the ones revealed in the study from Langner et al. (2016), except for the “Drop in Love” type. Although both Langner et al. (2016) and Gumparthi et al. (2021) investigated the temporal development of brand love relationship trajectories by means of a qualitative approach, Schmid and Huber (2019) statistically validated the development of brand love relationships based on Batra’s brand love framework (Batra et al., 2012). They adopted six brand love dimensions derived from the original brand love dimensions outlined by Batra et al. (2012) (i.e., “Self-brand integration,” “Positive emotional connection,” “Passion-driven behaviors,” “Long-term relationship,” “Attitude valence,” and “Anticipated separation distress”). “Self-brand integration” is composed of four sub-components (i.e., “Current self-identity,” “Desired self-identity,” “Attitude strength,” and “Life meaning”). “Positive emotional connection” is composed of three sub-components (i.e., “Emotional attachment,” “Positive affect,” and “Intuitive fit”). “Passion-driven behaviors” are composed of three sub-components (i.e., “Passionate desire to use,” “Willingness to invest,” and “Things done in the past”). They categorized the development of the brand love relationships into four lifecycle stages (i.e., “Exploration,” “Expansion,” “Maturity,” and “Decline”) based on the assumption that a relationship lifecycle in business, which is between firms or between salesperson and consumers, follows an inverse U-shaped track (Dwyer et al., 1987; Jap and Anderson, 2007; Palmatier et al., 2013). The inverse U-shaped trajectory means that the relationship lifecycle has dynamical patterns such as growth, flattening, and decline. The “Exploration” stage is the early stage of brand love relationships, which includes the first contact with a brand. Consumers get to know the characteristics of a brand from “Exploration” to “Expansion.” Over time, the relationship between consumers and a brand reaches the “Maturity” stage. After a stable relationship is attained in the “Maturity” stage, the momentum of their relationship begins to “Decline.” They investigated the fluctuations and differentiations of the brand love components between adjacent lifecycle stages. Their study demonstrated that the brand love relationship lifecycle is almost similar to the interpersonal romantic love relationship lifecycle even though some differences were observed between these relationships. It should be noted that their findings do not mean that brand love relationships are completely identical to interpersonal romantic love relationships. Concretely, at the “Exploration” stage, emotion-related constructs such as the “positive affect” sub-component and “Attitude valence” component got a significantly higher score, compared to other components and sub-components. In the “Expansion” stage, most of the components got a significantly higher score than those of the “Exploration” stage components. However, scores of the two sub-components (“Passionate desire to use” and “Willingness to invest”) in the “Passion-driven behavior” component were not significant. Moreover, the score of the “Passionate desire to use” sub-component was lower than that of the “Exploration” stage. They pointed out that this is a distinctive characteristic from interpersonal romantic love context. In the “Maturity” stage, although all indices of brand love relationship components were superior to those of the “Expansion” stage, the margin of increase was moderate. Interestingly, although the individual scores of self-related sub-components of the “Self-brand integration” (“Current self-identity” and “Desired self-identity”) were increased, they were not statistically significant compared to the “Positive affect” sub-component. Finally, in the “Decline” stage, all indices exhibited downward trends.

**FIGURE 2:**
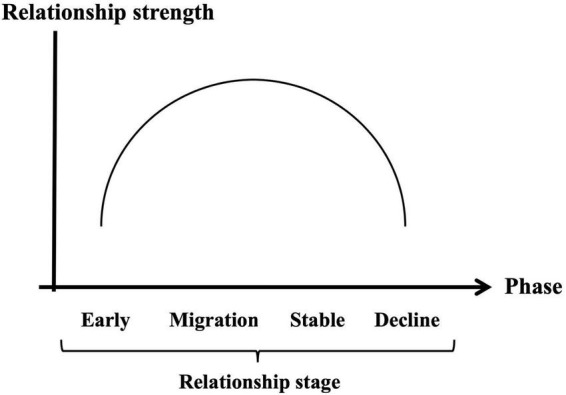
Generalized brand love relationship dynamics based on inverse U-shape model.

Taken together, the developmental trajectory of brand love relationships can be generally classified into four phases (i.e., early stage, migration stage, stable, and decline stage) like interpersonal romantic love relationships. Thus, positioning the relationship strength such as the brand love components (Batra et al., 2012) and the intensive love feelings (Langner et al., 2016; Gumparthi et al., 2021) on the vertical axis, and positioning the relationship stages on the horizontal axis, the dynamics of brand love relationships can be generally organized as shown in Figure 2. In the early phase including the first contact, emotional constructs played a crucial role in developing brand love relationships. In the developing phase from the early to migration stage, most of the brand love-related constructs got higher scores or positions than in the previous phase which means that the relationship was reinforced. This developmental phase phenomenon in brand love relationships is the same as that in interpersonal romantic love relationships. Interpersonal romantic love relationships during the developmental phase are enhanced by a reinforcement learning system (Langeslag et al., 2014; Edalat, 2015). Moreover, according to Schmid and Huber (2019), the indices of motivation-related constructs driven by intensive passionate emotion were consistently flat around the low score level across all the phases unlike the interpersonal romantic love relationships.

In this way, detailed theoretical and empirical studies on the dynamics of brand love relationships have been conducted. However, there are no studies on the neural mechanisms underlying brand love relationship dynamics. Although our previous study showed that activated brand regions in brand love relationships were largely different from those in the interpersonal romantic love relationships, it did not assess the dynamics of relationships between brands and consumers in terms of neural mechanisms. As many brand love studies noted (Ahuvia, 1992, 1994, 2005a,2005b; Albert et al., 2008; Batra et al., 2012; Albert and Merunka, 2013; Langner et al., 2015; Bagozzi et al., 2017), elucidating commonalities and differences between the mental processes involved in loving objects and those involved in loving persons can help in understanding the perceptions of the consumers. Therefore, the present study aims to identify the neural mechanisms of brand love relationships and interpersonal romantic love relationships by comparing distinct brain regions of each love relationship across all relationship stages and try to decode the mental processes derived from these neural mechanisms. Since the present study focuses on the dynamics of the relationship, the ascendant (product quality and brand awareness) and consequence (brand loyalty, willingness to pay a price premium, and positive word of mouth) of brand love are also included in the research objects as the series of brand love phenomena. In the present study, several hypotheses are validated to achieve the research objectives. Hypotheses to be assessed are as follows:

Hypothesis 1: If the neural mechanisms of brand love relationship dynamics are the same as those of the interpersonal romantic love relationship dynamics, activation of the same brain regions should be observed and the same constructs of the mental processes should be decoded across all stages.

Hypothesis 2: If relationships between consumers and brands are reinforced during term from the early stage to migration like the interpersonal romantic love relationships, reinforcement learning-related brain regions should be observed as shared brain regions between brand love and interpersonal romantic love relationships in the early stage and reinforcement learning-related constructs should be distinctively decoded in the early stage.

Hypothesis 3: If the function of motivation-related constructs, which is driven by intensive passionate emotion, in brand love relationship dynamics is weaker than that in interpersonal romantic love relationship dynamics, motivation-related brain regions should be weakly observed, and motivation-related constructs should be weakly decoded across all stages.

## Materials and methods

**FIGURE 3:**
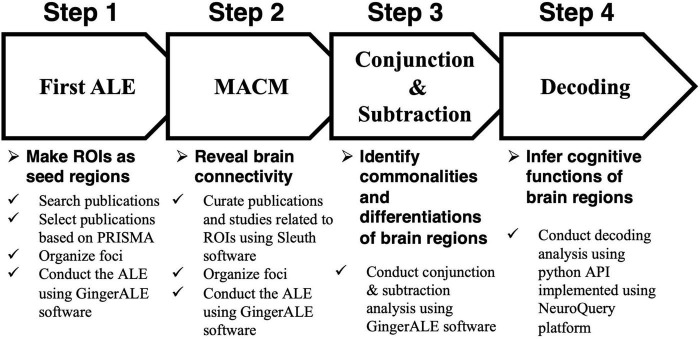
Steps of the analysis. ALE, activated likelihood estimation; ROI, region of interest; PRISMA, preferred reporting items for systematic reviews and meta-analyses; MACM, meta-analytical connectivity modeling; API, application programming interface.

Assuming that the relationship dynamics of both brand love and interpersonal romantic love relationships develop across four stages, we conducted an analysis to assess several hypotheses by identifying the neural mechanisms of commonalities and differences between brand love and interpersonal romantic love relationships at each stage using a quantitative neuroimaging meta-analysis. The following procedures were carried out and the analysis pipeline is described in Figure 3. First, we conducted a quantitative neuroimaging meta-analysis to detect each love relationship-related brain region in each stage. In this study, an activated likelihood estimation (ALE) was adopted as a method of quantitative neuroimaging meta-analysis. The detailed explanation of the ALE is described in section “A quantitative neuroimaging meta-analysis approach and an activated likelihood estimation method.” Second, since identifying brain networks has become a major approach when analyzing mental processes (Seeley et al., 2007; Buckner et al., 2008; Menon, 2015), I tried to precisely identify the brain networks related to each relationship stage using meta-analytical connectivity modeling (MACM). MACM is a data-driven approach that reveals brain networks by identifying co-activated brain regions (Robinson et al., 2010; Laird et al., 2013). A detailed explanation of the MACM is described in section “Meta-analytical connectivity modeling.” Third, a conjunction analysis was conducted in each stage to identify the overlapping and distinctive brain regions between brand love and interpersonal romantic love relationships. When conducting the conjunction analysis, the activated brain regions in each stage, which were obtained by the MACM, were used. A detailed explanation of the conjunction and subtraction analysis is described in section “Conjunction and subtraction analysis.” Finally, we conducted a decoding analysis to infer and interpret the cognitive functions of these brain regions in each love relationship in each stage using the NeuroQuery platform to avoid reverse inferences. A detailed explanation of the decoding analysis is described in section “Decoding analysis.”

### A quantitative neuroimaging meta-analysis approach and an activated likelihood estimation method

Several approaches for quantitative neuroimaging meta-analysis have been proposed. There are two types of quantitative neuroimaging meta-analysis methods. One type is the image based meta-analysis method (IBMA), which uses actual neuroimaging data. Although this approach is robust because it uses actual experimental brain image data, it is difficult for researchers to access these neuroimaging data for their study. The other type of quantitative neuroimaging meta-analysis method is to use peak coordinates reported in studies. This approach is referred to as the coordinate-based meta-analysis method (CBMA). The CBMA approach is more feasible for obtaining data because the coordinates are reported in a paper. In this study, we decided to adopt the CBMA method for quantitative neuroimaging meta-analysis. Moreover, we adopted the ALE method (Eickhoff et al., 2009) among the CBMA methods because the ALE method is the most popular among them (Acar et al., 2018). Since many analysis tools for the ALE method are available (Fox and Lancaster, 2002; Fox et al., 2014), this method is more viable for conducting a quantitative neuroimaging meta-analysis. Furthermore, the activated neuroimages produced with the ALE method were validated in comparison with the results from the IBMA, hence the effectiveness of the ALE method has been ensured (Salimi-Khorshidi et al., 2009). We conducted the meta-analysis based on publications from January 2000 to January 2022 that were indexed in PubMed database and found using keywords related to the research objective (i.e., Brand love relationships: “brand,” “consumer,” “fMRI,” “neural,” “choice,” “purchase,” “decision-making,” and “preference”/Interpersonal romantic love relationships: “romantic,” “love,” “fMRI”). We then added publications from other databases, such as Plassmann’s list (Plassmann et al., 2012) on branding study. (3) Selected publications for meta-analysis from searched and gathered publications, according to the following criteria: (i) Duplicated publications were eliminated (ii) Title and Abstract screening; studies which were irrelevant to the objective and theme of this study, were eliminated. Criteria for eliminating are as follows. (ii-1) fMRI was not used, (ii-2) Coordinates of the activated brain regions were not reported, (ii-3) Studied participants with a disease or reported the main results with diseased experimental participants (iii) Full text screening; studies in which brand logo-related objects were not used as experience stimuli, were eliminated. We considered whether the studies were conducted in a consumption context. Although the experimental stimuli without brand logos were used in both Klucharev et al. (2008) and Plassmann et al. (2008), both studies were adopted as publications for samples of this meta-analysis. Findings in these studies can be broadly interpreted as those in which brand logos were used as experimental stimuli since these studies were referenced in the study investigating that brand equity has influences on the decision making of the consumers (Plassmann et al., 2012). According to Plassmann et al. (2008), a wine with a higher price was associated with high quality and favorability. This finding is presumed to have the same effects of brand associations (Aaker, 1991; Keller, 1993). Klucharev et al. (2008) investigated that endorsement of experts has influenced the preferences of the consumers. This finding can be interpreted as the effect of brand knowledge. Regarding broadly interpreting and adopting publications for this meta-analysis, there might be insufficient publications employed in each stage in the present analysis if publications are going to be classified into four stages. Additionally, the product categories to which the experimental stimuli belonged (for example, consumer package goods, durable goods, or confectionary) did not matter. Regarding the interpersonal romantic relationships, unfaithful romantic love studies and studies with missing relationship durations were excluded. (iv) Activated foci of brain regions needed to be reported within the three-dimensional stereotactic space of the Talairach or the Montreal Neurological Institute (MNI) in the paper. (v) Relationship duration; (v-1) In brand love relationships, since relationship durations between brands and consumers were not described, the eligibility of the articles with respect to the stages of the brand love relationship was assessed based on levels of relationship strength such as the emotions of the consumers and self-integrity toward brands in each stage, according to the considerations of previous studies (Langner et al., 2016; Schmid and Huber, 2019; Gumparthi et al., 2021). According to the studies by Langner et al. (2016) and Gumparthi et al. (2021), the intensity of feelings toward brands becomes higher and gradually becomes moderate over time from the early stage, including the initial contact phase, to the current stage, except in the “Bumpy road (Type 4)” study typified by Langner et al. (2016) and “Drop in love” study typified by Gumparthi et al. (2021). Schmid and Huber (2019) described that the time dynamics of the relationship strength are not assessed according to the relationship length, but according to four stages based on subjective reports describing the inverse U pattern of the relationship life cycle in the business management studies (Dwyer et al., 1987; Jap and Anderson, 2007; Palmatier et al., 2013). Given that the present study considers that the relationship strength changes at each stage according to the inverse U pattern similar to the study by Schmid and Huber (2019), study selection was conducted based on the following criteria. In the early stage, a phase that includes first contact with the brands, although consumers start being familiar or make positive or negative decisions regarding the brands, they have limited knowledge of, infrequent contact with, and no emotional attachment to brands. The migration stage is an intermediate phase between the early and stable stages. Consumers have some types of emotional attachment (liking or favorite) toward brands in this stage. The stable stage is a phase where the relationship between the brand and the consumers matures and is well-established. Consumers have a high level of emotional attachment with brands (the most favorite brand, the most frequently buying, and the most preferred brand). The decline stage, which leads to breaking up relationships such as ceasing the usage of brands, is a phase where ties of relationships were weakened by decreasing the emotions and self-relevant thoughts toward the brands. Therefore, studies, in which it was difficult to infer the relationship strength, were eliminated. Deppe et al. (2005) and Kato et al. (2009) did not directly assess the brand love relationships. However, since they used familiar brands as experimental stimuli and did not assess the emotion and affection to the brands, I considered the relationship stage of the experimental participants in their studies to be almost parity to the state of early stage relationships. Plassmann et al. (2007) assessed the framing effect based on loyalty and did not directly assess the brand love. Since brand love is the ascendant of brand loyalty and their study can be positioned as the series of brand love relationship dynamics, Plassmann et al. (2007) was classified into the stable stage. Regarding Yoon et al. (2006), their study validated the discrepancy of brain activation between the brand and the person from the view of self-relevant constructs and did not directly assess the brand love. The study was classified into the stable stage since the brands used in their study have high familiarity and were associated with self-relevant constructs, which are crucial constructs of brand love. While Weber et al. (2007) assessed loss aversion for goods, not brand love, we believe that the implications of that study may also be relevant to the emotion of separation distress in the stable stage of brand love relationships. (v-2) In the interpersonal romantic love relationships, since relationship durations were described in most of the studies, each stage was classified based on these durations. The minimum relationship duration was 3 months, and the maximum relationship duration was 290.16 months. The mean relationship duration was 49.08 months, and the median relationship duration was 14.2 months. By referencing the median and mean values of the of relationship durations as norm values, studies were assigned to each stage. Therefore, the studies of the early stage romantic love relationships were defined as studies with relationship durations from 1 to 12 months, which is somewhat shorter than the median. Since the term of 49.08 months was presumed to be a very long duration to define the terminal durations of the migration stage, studies of migration-stage romantic love relationships were defined as studies with durations between 12 and 36 months, Studies of the stable stage of romantic love relationships were defined as studies with relationship durations over 36 months.

**FIGURE 4:**
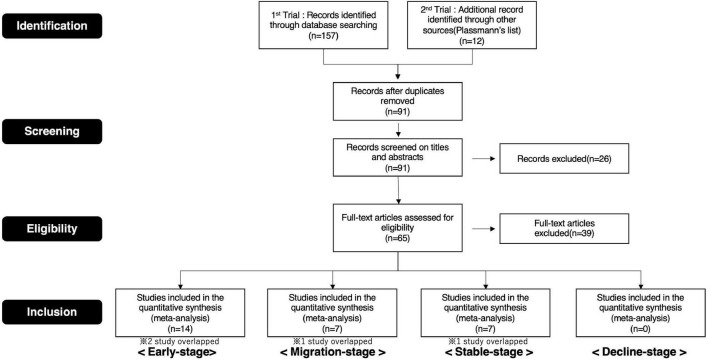
PRISMA flow diagram (brand love relationships).

**FIGURE 5:**
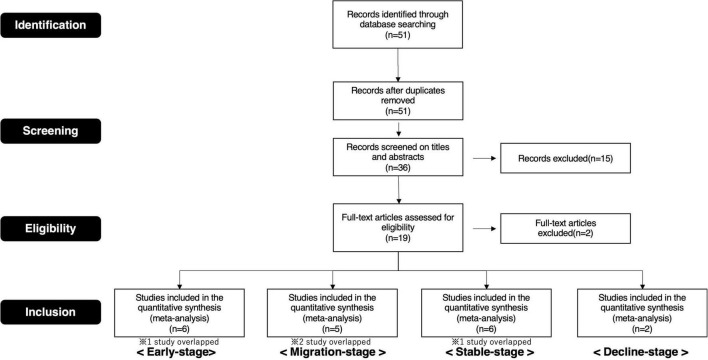
PRISMA flow diagram (interpersonal romantic love relationships).

Therefore, we selected publications for this study based on these procedures (Supplementary Tables 1A–C, 2A–C. Details of the screening process are depicted in the preferred reporting items for systematic reviews and meta-analyses (PRISMA) flow diagram (Figures 4, 5).

The ALE is a method that makes a brain activation map using foci gathered from selected publications, based on a statistical method. Three types of brain activation maps can be created by statistical methods (i.e., modeled activation maps, ALE maps, and thresholded activation maps) based on these foci. First, modeled activation map is created depending on the gaussian probability density function. Therefore, the more the input foci with a large experimental sample size, the more the probability with which the activated brain regions are accurately estimated because the variances of the gaussian probability density function are minimized. These modeled activation maps are calculated for each focus. Second, these modeled activation maps are aggregated and calculated into a united map. This united map is referred to as an ALE map. Finally, the ALE map is compared with the maps created by the null distribution and a more accurate ALE map is produced. The thresholded ALE map is obtained through a permutation test. The permutation test is conducted at each voxel between the ALE map and the randomness map created by the null distribution (Eickhoff et al., 2009; Turkeltaub et al., 2012). The thresholded ALE map obtained by these procedures is used for analysis in this study. To calculate the thresholded ALE map, we adopted the GingerALE version 3.02.^1^ The GingerALE is implemented for calculating the ALE algorism (Fox and Lancaster, 2002; Fox et al., 2014). Foci in each stage of each love relationship were organized in each text file. In this study, six files were created (the early, migration, and stable stages in each love relationship). These text files were used as input files for the GingerALE. We set a brain coordinate system as the MNI space. Since the false discovery rate (FDR) was not recommended in the preference menu of the GingerALE manual, the FDR was not used in this study. The detailed parameters were as follows. Given that it was difficult to conduct the ALE using a cluster level threshold because of the insufficient sample size, the first ALE was performed using thresholds that were set as *p* > 0.001 (uncorrected) and a minimum cluster size of 100 mm^3^. These parameters are the same parameters used in our previous brand love-related study (Watanuki and Akama, 2020). A more conservative threshold (*p* > 0.0001; uncorrected) was applied when calculating ALE values using data from the brand love relationships in the stable stage. To view the file, the Mango software (version 4.1),^2^ which is a brain image viewer, was used. The file was overlaid onto a canonical anatomical T1 brain template in the MNI space.

### Meta-analytical connectivity modeling

The MACM is a method used to identify brain regions correlated with the seed brain regions using an ALE algorithm. This ALE analysis is referred to as “second ALE” in the present study. Since Smith et al. (2009) reported that the brain networks revealed by MACM were consistent with the results of brain networks analyzed by a resting state networks approach, MACM is an effective approach for identifying brain networks. When executing the MACM, the regions revealed by conducting a quantitative neuroimaging meta-analysis at the first trial were used as seed brain regions. Since MACM was performed by using huge data and brain regions related to ROI were thresholded, significant brain connectivity could be obtained. Through conducting the ALE in two steps, we could obtain robust brain network images and locations regarding each stage of both love relationships. When curating brain regions correlated with the seed regions, we used the BrainMap database. The BrainMap database includes two types of databases. One type is the functional database. The other type is the voxel-based morphometry (VBM) database. We used the former type of database because our study is subject to a functional brain activation study with fMRI. The functional database in the BrainMap database covers 3,406 papers, 111 paradigm classes, 76,016 subjects, and 131,598 experiments. The MACM was conducted using the following procedures: **(i)** Set all brain regions calculated by the first ALE, that is, the brain regions related to each stage in each love relationship, as seed regions, **(ii)** Curate studies related to each seed region *via* the Sleuth version 3.0.4,^3^ from the BrainMap database. The Sleuth software is a curating tool for matching brain regions between the curated studies and the seed regions (Robinson et al., 2010; Vanasse et al., 2018). We set several appropriate conditions when curating studies, as follows: (ii-a) Context in the experiments was set as “Normal Mapping (experiments using normal participants who are not under the influences of drug and are not in the middle of the treatment from a disease);” (ii-b) Activation in the experiments was set to “Activation Only;” (ii-c) Imaging modality in the experiments was set to “fMRI;” and (ii-d) MNI image in the location set each seed region obtained by the first ALE. **(iii)** After the coordinates related to the seed regions were organized and aggregated by the Sleuth software, these coordinates were produced in text files. Six text files were prepared for conducting the second ALE. Brain coordinates that were correlated with brain regions related to each stage of each love relationship were stored in each text file. **(iv)** Conduct the second ALE using the GingerALE software. The text files obtained at the step (ii-d) were used as input files. When calculating the ALE algorism, we set the parameters according to Woo et al. (2014). The detailed parameters were as followed; (1) Cluster-level correction for multiple comparisons at *p* = 0.05, (2) Cluster-forming threshold of *p* = 0.001, (3) Permutation size = 5,000. The thresholded ALE maps were outputted as NIfTI files. When calculations of the ALE did not work in applying these parameters because of the insufficient sample size and exceeding the abilities of my personal computer specks, the ALE was conducted by setting the permutation size as 2,000 permutations. In case the calculations with 2,000 permutations did not work, the permutation size was set as 1,000. By testing the data of stable-stage brand love relationships when conducting the MACM, results with 5,000 permutations were equivalent to those with 2,000 permutations and mostly similar to those with 1,000 permutations. In testing with 1,000 permutations, the calculated peak coordination was equivalent to those with 5,000 permutations. Only small discrepancies of the maximum ALE score between the results with 5,000 and 1,000 permutations were confirmed (5,000 permutations = 0.14449802 and 1,000 permutations = 0.14449804). Eventually, the conducted analysis and permutation numbers show that the analysis using 5,000 permutations was the stable stage of brand love and the early stage of interpersonal romantic love relationships. While the analysis using 2,000 permutations was the migration stage of brand love, migration stage of interpersonal romantic love, and stable stage of interpersonal romantic love relationships. Finally, the analysis using 1,000 permutations was the early stage of brand love relationships.

### Conjunction and subtraction analysis

A conjunction and subtraction analysis were performed using GingerALE software. In this analysis, we aimed to identify the overlapping and distinctive brain regions in each stage, as well as the statistical significance of each region. Since overlapping and distinctive brain regions in the two stages were compared with the brain regions produced by the null distribution, each significant brain region was generated based on an appropriately set significance level. Parameters set for this conjunction analysis were as follows: (i) *p*-value = *p* < 0.01, (ii) Number of permutations = 10,000, (iii) Minimum cluster size = 100 mm^3^.

### Decoding analysis

A decoding analysis was conducted based on the results of the conjunction and subtraction analysis. To interpret the cognitive functions of the identified brain regions, and to avoid the reverse inference problem, a decoding analysis was conducted using the NeuroQuery platform^4^ (Dockès et al., 2020). The NeuroQuery platform is an automated neuroimaging meta-analysis platform that stores 13,459 studies, and 6,308 terms and phrases. The application programming interface (API) for a decoding analysis is implemented by Python language.^5^ Vocabulary size covered in the NeuroQuery is much superior to the Neutosynth platform^6^ (Yarkoni et al., 2011), which stores 1,314 terms. Further, terms and phrases covered in the NeuroQuery are calculated based on full-text articles and selected based on an occurrence rate of more than 0.05% of the publications. This means that NeuroQuery can appropriately interpret input neuroimages. NeuroQuery accepts activated brain image data with NIfTI file format. When inputting an activated brain image data, the NeuroQuery calculates terms and phrases, which are related to activated brain regions. The procedure of analysis with NeuroQuery is described in Supplementary Figure 1. These terms and phrases are produced along with the similarity score (Supplementary Figure 1B), which is the matched degrees between the inputted activated brain regions (Supplementary Figure 1A) and the terms related to the brain regions (Supplementary Figure 1H). The score is calculated with ranges from 0.00 (not at all matched terms) to 1.00 (the most matched terms). “In expansion” and “Publication related to the query” fields are helpful for inferring constructs of the decoded terms (Supplementary Figures 1F,G). Since the terms and phrases stored in the NeuroQuery platform were objectively analyzed by a text mining technique, there are some terms and phrases that were difficult to interpret. In case that the decoded terms apparently express mental processes-related terms such as “reward,” “emotion,” and “self-referential,” the decoded terms were easy to interpret. However, since some terms such as “flexibility” and “outcome” are generally used, it was difficult to specifically interpret these terms. Since “outcome” is used in a wide variety of situations, it is difficult to interpret the term appropriately and objectively. We could analyze the related terms to the target term (in this case, “outcome”) using “In expansion” and “Publication related to the query” (Supplementary Figures 1C,D). The related terms were produced in the “In expansion” field (Supplementary Figures 1E,F). According to results of the “In expansion” field, the term “outcome” is related to “reward” (Supplementary Figures 1E,F). Moreover, in the “Publication related to the query” field, reward-related publications were listed (Supplementary Figure 1G). Therefore, in this study, the uninterpretable terms were inferred according to this procedure.

## Results

### First activated likelihood estimation

**FIGURE 6:**
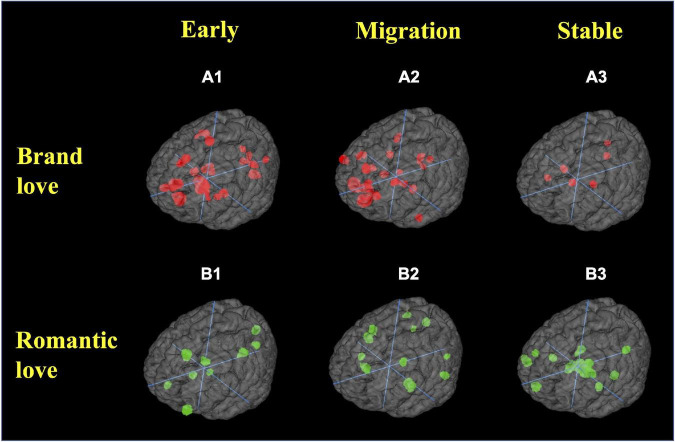
Region of interest. **(A)** Brand love relationships; **(A1)** early stage; **(A2)** migration stage; **(A3)** stable stage. **(B)** Interpersonal romantic love relationships; **(B1)** early stage; **(B2)** migration stage; **(B3)** stable stage. All crosshairs = (0,0,0).

First, the brain regions revealed for each stage in each love relationship are shown in Figure 6 and Supplementary Tables 3A,B detailed as follows.

In the brand love relationships, calculated peak coordinates of brain regions in the early stage of brand love relationships were the caudate (head and body), parahippocampal gyrus (BA28, amygdala), anterior cingulate gyrus (BA32), medial frontal gyrus (BA6, BA8, BA10), thalamus, lingual gyrus (BA17, BA18), parietal regions (precentral gyrus < BA4 >, postcentral gyrus < BA2 >), insula (BA13), and cuneus (BA17). The calculated peak coordinates of the brain regions in the migration stage of brand love relationships were the medial frontal gyrus (BA6, BA9, BA10), anterior cingulate gyrus (BA24), cerebellum, temporal regions (superior temporal gyrus < BA13 >, middle temporal gyrus < BA21 >, and inferior temporal gyrus < BA21 >), posterior cingulate gyrus (BA29, BA30), middle frontal gyrus (BA10), parahippocampal gyrus (BA35), caudate (head and body), and inferior frontal gyrus. The calculated peak coordinates of the brain regions in the stable stage of brand love relationships were the basal ganglia (putamen, caudate head, and body), posterior cingulate gyrus (BA23), precuneus (BA31), and insula (BA13).

In the interpersonal romantic love relationships, the calculated peak coordinates of the brain regions in the early stage of interpersonal romantic love relationships were the precuneus (BA7), posterior cingulate gyrus (BA30), inferior frontal gyrus (BA10), anterior cingulate gyrus (BA24, BA32), cuneus (BA17), midbrain (mammillary body), and caudate (head and body). The calculated peak coordinates of the brain regions in the migration stage of interpersonal romantic love relationships were the insula (BA13), claustrum, fusiform gyrus (BA37), precuneus (BA7), anterior cingulate gyrus (BA24), cingulate gyrus (BA32), superior frontal gyrus (BA8), parietal regions (precuneus < BA7 >, inferior parietal lobule < BA40 >), putamen, and lingual gyrus (BA17). The calculated peak coordinates of the brain regions in the stable stage of interpersonal romantic love relationships were the midbrain (subthalamic nucleus, mammillary body), basal ganglia (medial globus pallidus, caudate tail, putamen), thalamus, hippocampus, middle frontal gyrus < BA46 >, cuneus (BA23), medial frontal gyrus (BA9, BA10), claustrum, and insula.

### Meta-analytical connectivity modeling

The MACM was conducted by setting the brain regions obtained from the first ALE as the seed regions according to the procedures described in the “Materials and Methods” section.

#### Curated results in the brand love relationships

For the early stage of brand love relationships, since the brain regions obtained by the first ALE were too large to curate the related coordinates *via* the Sleuth software, the brain regions needed for setting the ROI were divided into three groups (i.e., Group 1: Cluster 1, Group 2: Clusters 2 and 3, and Group 3: Clusters 4–16). The curated results in group 1 yielded 60 papers that included 70 experiments, 989 foci, and 962 subjects. The curated results in group 2 yielded 117 papers that included 145 experiments, 2,014 foci, and 2,300 subjects. The curated results in group 3 yielded 130 papers that included 131 experiments, 2,175 foci, and 2,135 subjects. The duplicated foci among these results were eliminated. Eventually, 4,799 foci, 330 experiments, and 5,780 subjects were used as input data for the MACM with the second ALE. For the migration stage of the brand love relationships, since the brain regions obtained by the first ALE were too large to curate the related coordinates *via* the Sleuth software, the brain regions needed for setting the ROI were divided into three groups (i.e., Group 1: Cluster 1, Group 2: Cluster 2, and Group 3: Clusters 3–21). The curated results in group 1 yielded 50 papers that included 57 experiments, 654 foci, and 884 subjects. The curated results in group 2 yielded 44 papers that included 55 experiments, 842 foci, and 1,052 subjects. The curated results in group 3 yielded 48 papers that included 59 experiments, 997 foci, and 855 subjects. The duplicated foci among these results were eliminated. Eventually, 2,296 foci, 157 experiments, and 2,934 subjects were used as input data for the MACM with the second ALE. For the stable stage of the brand love relationships, the curated results for setting the brain regions yielded 44 papers that included 47 experiments, 793 foci, and 759 subjects. The obtained data were used as input data for the MACM with the second ALE.

#### Results of the second activated likelihood estimation in the brand love relationships

**FIGURE 7:**
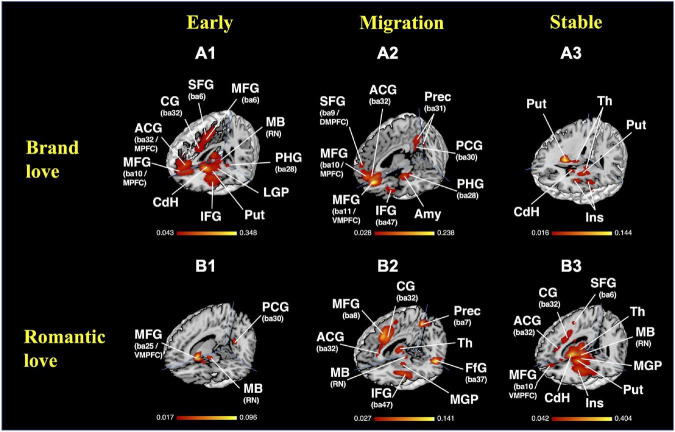
Results of the MACM. **(A)** Brand love relationships; **(A1)** early stage, crosshair = (0, −34, −6); **(A2)** migration stage, crosshair = (4, −64, −16); **(A3)** stable stage, crosshair = (28, −40, 2); **(B)** interpersonal romantic love relationships; **(B1)** early stage, crosshair = (10, −60, −14), **(B2)** migration stage, crosshair = (6, −62, −8); **(B3)** stable stage, crosshair = (6, −62, −8). MACM, meta-analytical connectivity modeling; BA, brodmann area; ACG, anterior cingulate gyrus; Amy, amygdala; CG, cingulate gyrus; CdH, caudate head; DMPFC, dorsomedial prefrontal cortex; FfG, fusiform gyrus; IFG, inferior frontal gyrus; Ins, insula; LGP, lateral globus pallidum; MB, midbrain; MFG, medial frontal gyrus; MGP, medial globus pallidum; MPFC, medial prefrontal cortex; PCG, posterior cingulate gyrus; PHG, parahippocampal gyrus; PreCG, precentral gyrus; Prec, precuneus; Put, putamen; RN, red nucleus; SFG, superior frontal gyrus; Th, thalamus; VMPFC, ventral medial prefrontal cortex.

The results of the MACM on each stage of brand love relationships are shown in Figures 7A1–A3 and Supplementary Table 4A. In the early stage of the brand love relationships, the parahippocampal gyrus (amygdala, BA28), basal ganglia (caudate head and body), anterior and mid insula (BA13), inferior frontal gyrus (BA9, BA45, BA47), middle frontal gyrus (BA46), precentral gyrus (BA44), anterior cingulate gyrus (BA32), medial frontal gyrus (BA6, BA8, BA9), superior frontal gyrus (BA6, BA9), cingulate gyrus (BA24, BA32), temporal regions (inferior temporal gyrus < BA32 >, and fusiform gyrus < BA32 >) were observed as the peak activated coordinates. In the migration stage of brand love relationships, the anterior cingulate gyrus (BA24, BA32), medial frontal gyrus (BA9, BA10), superior frontal gyrus (BA8, BA9), posterior cingulate gyrus (BA23, BA29), precuneus (BA31), temporal regions (superior temporal gyrus < BA13 >, middle temporal gyrus < BA39 >), parahippocampal gyrus (amygdala, BA28), anterior insula (BA13), inferior frontal gyrus (BA9, BA47), middle frontal gyrus (BA9, BA10), and precentral gyrus (BA6) were observed as the peak activated coordinates. In the stable stage of brand love relationships, the basal ganglia (caudate body, putamen), anterior insula (BA13), claustrum, thalamus, precentral gyrus (BA4, 6, 44), inferior frontal gyrus (BA44), and medial frontal gyrus (BA6) were observed as the peak activated coordinates.

#### Curated results in the interpersonal romantic love relationships

For the early stage of the interpersonal romantic love relationships, the curated results for setting the brain regions yielded 55 papers that included 63 experiments, 805 foci, and 965 subjects. The obtained data were used as input data for the MACM with the second ALE. For the migration stage of the interpersonal romantic love relationships, the curated results for setting the brain regions yielded 122 papers that included 136 experiments, 2,301 foci, and 2,133 subjects. The obtained data were used as input data for the MACM with the second ALE. For the stable stage of the interpersonal romantic love relationships, since the brain regions obtained by the first ALE were too large to curate the related coordinates *via* the Sleuth software, the brain regions needed for setting the ROI were divided into two groups (i.e., Group 1: Clusters 1 and 2, and Group 2: Clusters 3–11). The curated results in group 1 yielded 119 papers that included 144 experiments, 1,940 foci, and 2,260 subjects. The curated results in group 2 yielded 164 papers that included 188 experiments, 2,641 foci, and 2,948 subjects. The duplicated foci among these results were eliminated. Eventually, 4,581 foci, 332 experiments, and 5,575 subjects were used as input data for the MACM with the second ALE.

#### Results of the second activated likelihood estimation in the interpersonal romantic love relationships

The results of the MACM of each stage of the interpersonal romantic love relationships are shown in Figures 7B1–B3 and Supplementary Table 4B. In the early stage of the interpersonal romantic love relationships, the basal ganglia (putamen, caudate head), parahippocampal gyrus (amygdala), posterior cingulate gyrus (BA30), precuneus (BA7), inferior frontal gyrus (BA47), claustrum, midbrain (mammillary body), anterior insula (BA13), and extra-nuclear (BA13) were observed as the peak activated coordinates. In the migration stage of the interpersonal romantic love relationships, the claustrum, insula (BA13), thalamus, basal ganglia (putamen, medial globus pallidus, lateral globus pallidus, caudate head), midbrain (red nucleus), cingulate gyrus (BA24, BA32), anterior cingulate gyrus (BA24, BA32), inferior frontal gyrus (BA9), precentral gyrus (BA6), middle frontal gyrus (BA6, BA9, BA46), Sub-Gyral (BA6), parietal regions (superior parietal lobule < BA7 >, inferior parietal lobule < BA40 >, precuneus < BA7 >), fusiform gyrus (BA37), and cerebellum (culmen) were observed as the peak activated coordinates. In the stable stage of the interpersonal romantic love relationships, the basal ganglia (caudate head and body, putamen, lateral globus pallidus), midbrain (substania nigra), thalamus, temporal regions (Superior Temporal Gyrus < BA22 >, middle temporal Gyrus < BA37 >, fusiform gyrus < BA37 >), claustrum, parahippocampal gyrus (amygdala, BA36, hippocampus), medial frontal gyrus (BA6, BA9, BA10), inferior frontal gyrus (BA47), anterior cingulate gyrus (BA24, BA32), and cingulate gyrus (BA24, BA32) were observed as the peak activated coordinates.

### Conjunction and subtraction analysis

#### Results of the conjunction analysis

**FIGURE 8:**
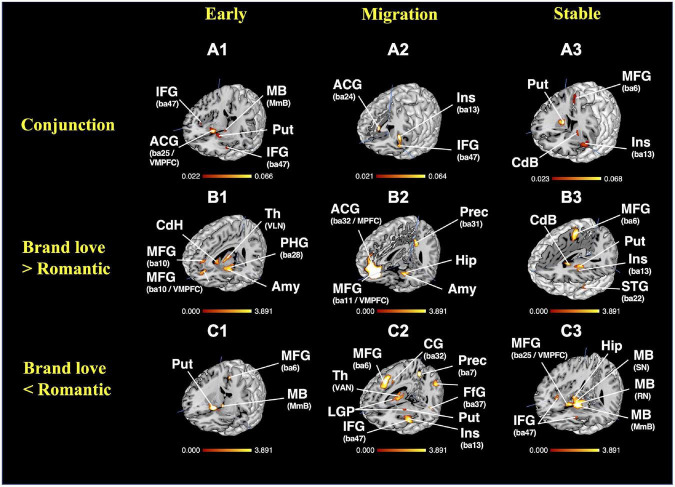
Results of the conjunction and subtraction analysis. **(A)** Results of the conjunction analysis; **(A1)** 3D view of overlapping brain regions in the early stage, crosshair = (44, −20, −9); **(A2)** 3D view of overlapping brain regions in the migration stage, crosshair = (2,17, −5); **(A3)** 3D view of overlapping brain regions in the stable stage, crosshair = (27,4, −4); **(B)** results of the subtraction analysis (Brand love > Interpersonal romantic love); **(B1)** 3D view of the distinctive brain regions activated in the early stage brand love relationships, crosshair = (−7, −39, −14); **(B2)** 3D view of the distinctive brain regions activated in the migration stage of brand love relationships, crosshair = (1, −47, −20); **(B3)** 3D view of the distinctive brain regions activated in the stable stage of brand love relationships, crosshair = (−1, −54,10); **(C)** results of the subtraction analysis (Brand love < Interpersonal romantic love); **(C1)** 3D view of the early stage of interpersonal romantic love relationships, crosshair = (27, −10, −13); **(C2)** 3D view of the migration stage of interpersonal romantic love relationships, crosshair = (11, −54, −8); **(C3)** 3D view of the stable stage of interpersonal romantic love relationships, crosshair = (37, −45, −13). BA, brodmann area; ACG, anterior cingulate gyrus; Amy, amygdala; CG, cingulate gyrus; CdB, caudate body; CdH, caudate head; DMPFC, dorsomedial prefrontal cortex; FfG, fusiform gyrus; Hip, hippocampus; IFG, inferior frontal gyrus; Ins, insula; LGP, lateral globus pallidum; MB, midbrain; MmB, Mammillary Body; MFG, medial frontal gyrus; MPFC, medial prefrontal cortex; PCG, posterior cingulate gyrus; PHG, parahippocampal gyrus; PreCG, precentral gyrus; Prec, precuneus; Put, putamen; RN, red nucleus; SFG, superior frontal gyrus; SN, Substania Nigra; Th, thalamus; VAN, ventral anterior nucleus; VMPFC, ventral medial prefrontal cortex.

To obtain the overlapping brain regions between the brand love relationships and interpersonal romantic love relationships, a conjunction analysis was conducted. Overlapping brain regions between the brand love relationships and interpersonal romantic love relationships in each stage are shown in Figures 8A1–A3 and Supplementary Table 5. In the phase of the early stage relationship, the overlapping brain regions were the basal ganglia (caudate head, putamen), amygdala, inferior frontal gyrus (BA47), anterior insula (BA13), midbrain (mammillary body), and thalamus. In the phase of the migration stage relationships, the overlapping brain regions were the anterior insula (BA13), inferior frontal gyrus (BA9, BA47), middle frontal gyrus (BA9, BA10), precentral gyrus (BA6), anterior cingulate gyrus (BA24), and lateral globus pallidus. In the phase of the stable stage relationships, the overlapping brain regions were the basal ganglia (caudate body, putamen), anterior insula, cingulate gyrus (BA24), precentral gyrus (BA44), medial frontal gyrus (BA6), and thalamus.

#### Results of the subtraction analysis

To obtain the distinctive brain regions in each love relationship, a subtraction analysis was conducted. The detailed results are shown in Figures 8 B1–B3,C1–C3 and Supplementary Tables 6–8.

During the early stage, the distinctive brain regions activated in the brand love relationships were the basal ganglia (medial globus pallidus, putamen, caudate body), thalamus, midbrain (substania nigra), parahippocampal gyrus (BA28), medial frontal gyrus (BA9), anterior cingulate gyrus (BA32), precentral gyrus (BA6), cerebellum (culmen), claustrum, and inferior occipital gyrus (BA19). The distinctive brain regions activated in the interpersonal romantic love relationships were the basal ganglia (caudate head, putamen), precuneus (BA7), posterior cingulate gyrus (BA30), midbrain (mammillary body), medial frontal gyrus (BA6), and anterior insula.

During the migration stage, the distinctive brain regions activated in the brand love relationships were the anterior cingulate gyrus (BA32), cingulate gyrus (BA31), precuneus (BA31), posterior cingulate gyrus (BA29), superior temporal gyrus (BA22), inferior parietal lobule (BA40), parahippocampal gyrus (amygdala, hippocampus, BA34), and inferior frontal gyrus (BA45, BA47). The distinctive brain regions activated in interpersonal romantic love relationships were the cingulate gyrus (BA32), medial frontal gyrus (BA6), anterior cingulate gyrus (BA24), insula (BA13), inferior frontal gyrus (BA13), fusiform gyrus (BA37), parietal regions (precuneus < BA7 >, inferior parietal lobule < BA40 >), thalamus, precentral gyrus (BA6), middle frontal gyrus (BA9, BA10), the basal ganglia (putamen, lateral globus pallidus), and sub-gyral (BA6).

During the stable stage, the distinctive brain regions activated in the brand love relationships were the basal ganglia (putamen), claustrum, precentral gyrus (BA4, 6, 44), medial frontal gyrus (BA6), thalamus, and anterior insula. In the distinctive brain regions activated in the interpersonal romantic love relationships, the brain regions in cluster 1 were the most broadly activated regions. Although only four brain regions were listed in Supplementary Table 8, multiple brain regions such as the thalamus, midbrain, hippocampus, and ventral striatum were included in cluster 1. The activated brain regions in cluster 2 were the inferior frontal gyrus (BA13) and extra-nuclear (BA13) regions. The activated brain regions in cluster 3 were the anterior insula (BA13).

### Decoding analysis

The decoded results, excluding the anatomical and uninterpretable terms, are shown in Tables 1–3. Terms listed in Tables 1–3 were ranked in the top 10 terms at each stage. Terms with the same similarity scores were ranked in the same position. The complete list of terms with similarity scores greater than 0.45 in each stage of each love relationship is presented in Supplementary Tables 9–11. “Dopamine” was a common decoded term regarding both overlapping and distinctive brain regions in this stage.

**TABLE 1:** Decoded results (early stage phase).

Brand love and interpersonal romantic love				Brand love > interpersonal romantic love				Brand love < interpersonal romantic love			
		
Rank	Term	Construct	SS	Rank	Term	Construct	SS	Rank	Term	Construct	SS
1	Outcome	D/Mt/**R**	0.96	1	Affect	**ES**/R	0.99	1	Self	D/R/**SR**	1
2	Reward	**R**	0.91	2	Avoid	**ES**/R	0.94	2	Positive	D**/ES**/R	0.78
3	Motivation	**R**/V	0.9	3	Emotional	**ES**	0.9	3	Theory	**D**/R/**SC**	0.73
4	Gain	Mt**/R**/V	0.89	3	Arousal	**ES**	0.9	4	Immediate	D/M/**R**	0.68
5	Substance	**R**	0.88	3	Negative	D/**ES**	0.9	5	Attribution	D**/R**/**SC**	0.66
6	Collection	Mt/**R**	0.83	4	Trust	ES/**SC**	0.89	6	Mentalizing	**D**/**SC**	0.65
7	Monetary	ES/**R**	0.82	5	Fear	**ES**/V	0.87	6	Outcome	D/**R**	0.65
8	Positive	D/**ES**/R	0.8	6	Avoidance	**ES**/R	0.83	6	Failure	**D**/R	0.65
9	Rewarding	**R**/V	0.79	7	Salient	**ES**/R/V	0.83	7	Sad	**ES**/**SC**	0.63
9	Reward processing	**R**	0.79	7	Self Report	ES/R/**SR**	0.81	8	Trial	Mt**/R**/V	0.62
10	Reinforcement	Mt**/R**/V	0.78	8	Emotional stimuli	**ES**/V	0.79	8	Positive negative	D**/ES**/R	0.62
10	Unexpected	**R**/V	0.78	9	Affective	**ES**	0.78	9	Mind	**D**/**SC**	0.61
10	Immediate	D/M/**R**	0.78	10	Negative affect	**ES**/R	0.77	9	Collection	**R**	0.61
-	-	-	-	-	-	-	-	10	Reward	**R**	0.6

The terms are sorted by order of the similarity score. Bold characters in the construct column represent the most influential mental process constructs. The terms are sorted by the order of the similarity score. SS, Similarity score; D, default mode network-related constructs; ES, emotional salience mental processes; M, memory-related mental processes; Mt, motor processing; R, reward processing; SC, social cognitive processing; SR, self-referential processing; V, visual processing.

**TABLE 2:** Decoded results (migration-stage phase).

Brand love and interpersonal romantic love				Brand love > interpersonal romantic love				Brand love < interpersonal romantic love			
		
Rank	Term	Construct	SS	Rank	Term	Construct	SS	Rank	Term	Construct	SS
1	Regulation	**CC**/**ES**	0.86	1	Network	**D**	0.88	1	Flanker	**CC**	0.74
2	Cognitive CONTROL	**CC**/D	0.85	2	Default	**D**	0.86	2	Feeling	**ES**/R/Ss	0.73
3	Norm	**SC**	0.73	3	Self REFERENTIAL	**D**/M/**SR**	0.82	3	Demand	**CC**/V	0.71
4	Economic	**R**	0.71	4	Referential	**D/SR**	0.8	3	Resource	**CC**/D/V	0.71
5	Emotion Regulation	**CC**/**ES**	0.66	5	Thought	**D**	0.75	4	Conflict	**CC**/ES/R	0.71
6	Social norm	M**/SC**	0.63	6	Conscious	**D**	0.74	5	Experience	**ES**/R	0.7
7	Wisconsin	**CC**/M	0.62	7	Network dmn	**D**	0.73	5	Difficult	**CC**/R/V	0.7
8	Impulse	**CC**	0.61	8	Midline	**D/SR**	0.72	6	Accuracy	**CC/Mt**/V	0.69
9	Sorting	**CC**	0.59	9	Dmn	**D**	0.71	6	Selection	**CC**/M/R/V	0.69
9	Strategy	**CC**	0.59	10	Default mode	**D**	0.7	6	Rejection	CC/ES/R/**SC**	0.69
9	Regulate	**CC**/D/ES	0.59	-	-	-	-	6	Reaction time	**CC**/Mt/R	0.69
9	Conflict	**CC**/ES/R	0.59	-	-	-	-	7	Interoceptive	**ES**/M	0.67
9	Regulate	**CC**/D	0.59	-	-	-	-	7	Autonomic	**ES**	0.67
10	Anxiety	**ES**	0.58	-	-	–	-	7	Norm	**SC**	0.67
-	-	-	-	-	-	-	-	8	Salience	D**/ES**/R	0.65
-	-	-	-	-	-	-	-	8	Bodily	**ES**/Ss	0.65
-	-	-	-	-	-	-	-	8	Partner	**SC**	0.64
-	-	-	-	-	-	-	-	9	Empathy	**SC**/Ss	0.64
-	-	-	-	-	-	-	-	10	Interference	**CC**	0.63

The terms are sorted by order of the similarity score. Bold characters in the construct column represent the most influential mental process constructs. The terms are sorted by the order of the similarity score. SS, Similarity score; D, default mode network-related constructs; CC, cognitive control processing; ES, emotional salience mental processes; M, memory-related mental processes; Mt, motor processing; R, reward processing; SC, social cognitive processing; SR, self-referential processing; Ss, somatosensory processing; V, visual processing.

**TABLE 3:** Decoded results (stable-stage phase).

Brand love and interpersonal romantic love				Brand love > interpersonal romantic love				Brand love < interpersonal romantic love			
		
Rank	Term	Construct	SS	Rank	Term	Construct	SS	Rank	Term	Construct	SS
1	Flexibility	**CC/ES**	0.77	1	Making	**DM**/**Mt**/R/V	0.64	1	Outcome	D/Mt/**R**	0.75
1	Making	**DM**/**Mt**/R/V	0.77	2	Flexibility	**CC/ES**	0.62	2	Reward	**R**	0.73
2	Dopamine	Mt/**R**	0.73	3	Experience	**ES**/R	0.55	2	Gain	Mt**/R**/V	0.72
3	Experience	**ES**/R	0.72	3	Inhibition	**Mt**/Ss	0.55	3	Motivation	**R**/V	0.72
4	Salience	D/**ES**/R	0.7	4	Feeling	**ES**/R/Ss	0.54	3	Collection	**R**	0.71
5	Gain	Mt/**R**/V	0.68	5	Motor	**Mt**	0.53	4	Substance	**R**	0.71
6	Motivation	**R**/V	0.67	6	Salience	D/**ES**/R	0.52	4	Monetary	**R**	0.67
6	Habit	**Mt**	0.67	7	Motor response	**Mt**	0.49	5	Loss	**R**	0.66
6	Feeling	**ES**/R/Ss	0.67	8	Inhibitory control	**CC**/Mt/R	0.48	5	Positive	D/**ES**/R	0.66
7	Uncertainty	**CC**/ES/R	0.66	8	Dopamine	Mt/**R**	0.48	5	Consumption	CC/D/**R**/V	0.65
7	Outcome	D/Mt/**R**	0.66	8	Partner	**SC**	0.48	6	Dopamine	Mt/**R**	0.65
7	Financial	CC/**R**	0.66	8	Interoceptive	**ES**/M	0.48	6	Self report	ES/R/**SR**	0.65
8	Desired	**R**/V/Ss	0.65	9	Effort	**CC**/Mt/**R**	0.47	6	Seeking	**R**	0.64
9	Decision	**DM**/M/**R**	0.64	10	Habit	**Mt**	0.46	7	Reward processing	**R**	0.64
9	Partner	**SC**	0.64	-	-	-	-	8	Anticipation	**ES**	0.64
9	Food	**R**/V	0.64	-	-	-	-	9	Unexpected	**R**/V	0.63
9	Love	**R**	0.64	-	-	-	-	9	Reinforcement	Mt/**R**/V	0.63
10	Effort	**CC**/Mt/**R**	0.63	-	-	-	-	9	Rewarding	**R**/V	0.63
10	Collection	**R**	0.63	-	-	-	-	10	Sharing	R/**SC**	0.63
-	-	-	-	-	-	-	-	10	Expected	**R**/V	0.62
-	-	-	-	-	-	-	-	10	Incentive	D/**R**	0.62

The terms are sorted by order of the similarity score. Bold characters in the construct column represent the most influential mental process constructs. The terms are sorted by the order of the similarity score. SS, Similarity score; D, default mode network-related constructs; DM, decision-making; CC, cognitive control processing; ES, emotional salience mental processes; M, memory-related mental processes; Mt, motor processing; R, reward processing; SC, social cognitive processing; SR, self-referential processing; Ss, somatosensory processing; V, visual processing.

Regarding the terms associated with the overlapping activated brain regions of the early stage of both love relationships, the reward-related terms such as “reward,” “motivation,” “outcome,” and “gain” were dominantly decoded. Remarkably, the reinforcement learning-related terms (“reinforcement learning” and “reinforcement”) were characteristically decoded. The other characteristically decoded term was “substance.” As for the distinctive decoded terms in the brand love relationships, the emotional salience-related terms such as “emotional,” “affect,” “arousal,” “negative,” and “fear” were dominantly decoded. Although the emotional salience-related terms were superiorly positioned, emotional salience- and reward-related terms (“relevant,” “adaptive,” “research”) were mixed in the top tiers of the decoded terms. The other characteristically decoded term was “trust.” In the interpersonal romantic love relationships, the self-referential related terms (“self”) were well matched with the distinctive activated brain regions. The other decoded terms were reward-related (“immediate,” “attribution,” “outcome,” and “reward”), default mode network (DMN)-related (“theory,” “mentalizing,” and “mind”), and emotional salience-related terms (“positive,” “positive negative,” and “sad”).

In the migration stage, the top tier of decoded terms regarding overlapping brain regions of both love relationships was dominated by the cognitive control processing-related terms (“regulation,” “cognitive control,” “emotion regulation,” “impulse,” “wisconsin,” “sorting,” “strategy,” “regulate,” and “conflict”). The other decoded terms were social norm-related terms such as “norm,” and “social norm.” The dominantly decoded terms regarding distinctive activated brain regions of the brand love relationships were the DMN-related terms such as “default,” “self-referential,” “thought,” “conscious,” “network dmn,” “midline,” “dmn,” and “default mode.” In the interpersonal romantic love relationships, although the social norm (“norm”) and reward-related (“learn”) terms were partly observed, the working memory (“flanker,” “demand,” “resource,” “conflict,” “difficult,” and “accuracy”) and emotional salience (“feeling,” “experience,” “interoceptive,” “autonomic,” and “salience”) related terms were dominantly decoded.

In the stable stage phase, the decoded terms regarding the overlapping brain regions of both love relationships were complexly mixed by multiple constructs such as reward (“gain,” “motivation,” “desired,” and “outcome”), cognitive control (“uncertainty,” “flexibility”), and emotional salience-related terms (“salience,” “feeling,” and “experience.”) The other characteristically decoded terms were “love” and “dopamine,” although these are related to reward processing. The decoded terms regarding the distinctive activated brain regions of the brand love relationships were emotional salience-related terms (“experience,” “salience,” “feeling,” and “interoceptive.”) The other characteristically decoded terms were “flexibility” and “habit.” These terms are also decoded in the overlapping brain regions. The term “flexibility” and “making” is best matched to the distinctive brain regions of the brand love relationships. Regarding the interpersonal romantic love relationships, the reward-related terms (i.e.; “outcome,” “reward,” “gain,” “collection,” “motivation,” “substance,” “monetary,” “consumption,” “seeking,” “reward processing,” “anticipation,” and “loss”) were dominantly decoded. The other characteristically decoded terms were “serotonin.”

## Discussion

### Anterior insula: Overlapping brain regions across all stages in both love relationships

Although both brand love and interpersonal romantic love relationships have resembled mental processes, innately distinctive mental processes might be underlaid in these love relationships. Given that the distinctive activated brain connectivity related to each love relationship was revealed, the terms with distinctively characteristic features in each love relationship were decoded.

The conjunction analysis across three stages identified that the anterior insula was a shared brain region between both brand love and interpersonal love relationships. Therefore, it is suggested that each love relationship might be a mental process that is mainly modulated by the anterior insula. The anterior insula is associated with interoceptive feelings (Craig, 2003, 2009), generating emotional salience (Taylor et al., 2003; Phan et al., 2004; Xia et al., 2017), impulsive feelings such as sexual emotion, addiction behaviors (Childress et al., 2008; Gola et al., 2015), and cognitive regulation (Seeley et al., 2007; Bressler and Menon, 2010; Menon, 2011). The anterior insula integrates this multiple processing into engaging in a wide variety of adaptive behaviors such as decision-making (Sridharan et al., 2008; Singer et al., 2009; Kurth et al., 2010; Menon and Uddin, 2010; Uddin et al., 2014, 2017; Jiang et al., 2015; Wang et al., 2019). Thus, considering the mental processes in which the anterior insula is engaged, each love relationship is presumed to be a mental process where the visceral-derived subjective feelings are underlaid. Given that the anterior insula is composed of two segregate regions, the ventral part and dorsal part, each region of the insula has different brain connectivity with other brain regions (Nelson et al., 2010; Deen et al., 2011; Touroutoglou et al., 2012). The ventral anterior insula has connections with the inferior frontal gyrus, superior frontal gyrus, pregenual part of the anterior cingulate gyrus, dorsal part of the anterior cingulate gyrus, temporal regions, posterior cingulate gyrus, basal forebrain, parahippocampal gyrus (amygdala, hippocampus, BA28, BA34), and the ventral striatum (Deen et al., 2011; Cerliani et al., 2012; Touroutoglou et al., 2012). While the dorsal anterior insula has connections with the dorsal part of the anterior cingulate gyrus, lateral sides of the prefrontal cortex, lateral sides of the orbitofrontal cortex, motor cortex, parietal regions, anterior part of the inferior frontal gyrus, temporal pole, and the dorsal striatum (Deen et al., 2011; Touroutoglou et al., 2012; Odriozola et al., 2016). The former brain network is engaged in emotional and affective mental processes, while the latter brain network is engaged in cognitive control and rational mental processes (Kurth et al., 2010; Touroutoglou et al., 2012; Chang et al., 2013; Uddin et al., 2014; Wager and Barrett, 2017).

### Early stage

The results of the conjunction analysis reveal that the ventral insula was co-activated with the ventral striatum and the parahippocampal gyrus including the amygdala. The ventral striatum is involved in a wide variety of reward processing activities (Elliott et al., 2000; Knutson et al., 2001; Haber and Knutson, 2010; Motoki et al., 2019), and engages in reinforcement learning (Haruno and Kawato, 2006). The ventral anterior insula, ventral striatum, amygdala, and hippocampus are composed of reward networks (Camara et al., 2009). The interconnection between the anterior insula and the ventral striatum is associated with addiction and substance dependence (Goldstein et al., 2009; Koob and Volkow, 2010; Everitt and Robbins, 2016; Peters et al., 2016; Voon et al., 2020). Moreover, the striatum, midbrain, amygdala, and thalamus are composed of a motivational network (Kelley et al., 2005). The decoded results also showed that the top tier of the decoded terms dominated the reward-related terms (“motivation” and “reward”), reinforcement learning-related terms, and addiction and dependence-related terms (“substance”). Thus, given that the common brain regions in each love relationship in the early stage phase mainly consist of components of the ventral insula pathway, these love relationships might be commonly underlaid by intensively motivated mental processing based on a reinforcement learning system.

Regarding the mental processes of the early stage brand love relationships, since the activations of the brain regions involving the ventral insula pathway were observed, the emotional mental processes might be involved in the mental processes of the brand love relationships. Besides these brain regions, the medial prefrontal cortex (MPFC), dorsal striatum, and parahippocampal gyrus (entorhinal cortex) were characteristically observed. The connection between the MPFC (especially the ventral MPFC) and the ventral striatum is a component of a neural currency network. The network is associated with reward-based subjective valuation (Kable and Glimcher, 2007; Tom et al., 2007; Bartra et al., 2013). Same as the ventral striatum, the dorsal striatum is also associated with reward processing (Balleine et al., 2007) and reinforcement learning (Packard and Knowlton, 2002; Samejima et al., 2005). Besides the reward processing, the dorsal striatum is associated with a positive effect (Phan et al., 2002). The ventral striatum is also associated with an emotion such as euphoria. Although the reward-related terms and emotional salience-related terms were decoded, the reinforcement learning-related terms were not decoded. Given that the amygdala has connections with the entorhinal cortex, these connections play a crucial role in emotional episodic memories (Kesner and Rogers, 2004; Phelps, 2004). This consideration is consistent with decoded results that emotional salience-related terms were dominated in the top tier positions. Interestingly, negative emotion terms such as “negative,” “avoid,” “avoidance,” and “fear” were decoded. The amygdala engages in fear-conditioned associative memories and is associated with unconscious negative emotional evaluations (LeDoux, 1992; Phelps et al., 2000; Izuma et al., 2019). Fear-conditioned memories are instantly acquired and persistently remain (Phelps, 2004; Poulos et al., 2009). Additionally, the amygdala engages in assessing trustworthiness (Bzdok et al., 2011; Santos et al., 2016). Untrustworthiness and avoidance behavior was correlated with the activation of the amygdala (Engell et al., 2007; Todorov, 2008). Since the terms such as “trust,” “avoid,” and “avoidance” was decoded, this result suggests that consumers evaluate whether a brand might deserve trustworthy or untrustworthy during early stage brand love relationships. These considerations suggest that it is important for brand managers to manage in a sensitive manner to prevent associating brands with negative emotional elements since it is more difficult to remove negative emotions attached to brands than they can imagine. Therefore, making decisions about brands is emotionally executed during the early stage. The distinctive brain networks of the brand love relationships in the early stage are speculated to be strongly involved in the reward and emotional processing.

Since activations of the ventral striatum and anterior insula were observed similar to those of the overlapping brain regions in the interpersonal romantic love relationships, both reward processing and intensive motivative mental processing might be involved in the mental processes of the relationships. Although the reward-related terms such as “immediate,” “attribution,” and “reward” were decoded at the top tiers, intensive motivative-related terms were not decoded at the higher scores (i.e.,: “substance” = 0.59, “motivation” = 0.56, and “substance use disorder” = 0.52). The decoded results of the emotional salience-related terms, such as “positive” and “sad,” can be speculated by activations of components of the ventral insula pathway. As described above, the ventral insula pathway is associated with emotional and affective processing (Kurth et al., 2010; Touroutoglou et al., 2012; Chang et al., 2013; Uddin et al., 2014; Wager and Barrett, 2017). Characteristically activated brain regions are the precuneus and medial part of the posterior cingulate cortex. These regions are a crucial component of the posterior part of the DMN (pDMN) (Damoiseaux et al., 2008). The DMN is one of the major intrinsic brain networks (Buckner et al., 2008). The DMN is associated with self-referential and social cognitive processes such as autobiographical memories, mentalizing, mind-wandering, theory of mind, and empathy (Buckner et al., 2008; Spreng et al., 2009; Andrews-Hanna, 2012; Mars et al., 2012; Xin and Lei, 2015; Davey et al., 2016; Kucyi et al., 2016). Principally, the pDMN is associated with social cognitive processes such as mentalizing and theory of mind (Mars et al., 2012; Esménio et al., 2020). Both mentalizing and theory of mind are mental processes involving the simulation of other people’s minds, and are associated with prosocial behaviors (Caputi et al., 2012; Majdandžiæ et al., 2016). The decoded terms such as “theory” (theory of mind-related term) and “mentalizing” might be associated with the pDMN. Moreover, the pDMN was also engaged in self-referential processing such as self-affirmation (Cascio et al., 2016), self-reflection (Johnson et al., 2006), and self-attribution. Regarding self-reflection, the pDMN is associated with duty and obligation (Johnson et al., 2006). The pDMN engages in external attribution during self-attribution (Jackson et al., 2006). Self-affirmation and self-esteem are associated with the satisfaction of romantic love relationships (Cramer, 2003). Thus, the pDMN is associated with taking the perspectives of others and focusing on an external environment while engaging in self-referential mental processes (Jackson et al., 2006; Leech and Sharp, 2014). Therefore, it can be postulated that the decoded term “self” has prosocial aspects in spite of the terms concerning the self-referential processing. Accordingly, the mental processes of the interpersonal romantic love relationships might be underlaid by social cognitive processing including the reward processing.

### Migration stage

The common brain regions composed of the salience network such as the dorsal part of the anterior cingulate gyrus and both the dorsal and ventral part of the anterior insula were observed. These brain regions are the main components of the salience network and are engaged in the mental processes related to attention, control, regulation, interoceptive, and autonomic processes (Seeley et al., 2007; Menon and Uddin, 2010; Menon, 2015). The other characteristically revealed brain region is the lateral side of the frontal cortex. These regions are associated with cognitive control processing and are activated during tasks where the cognitive load is demanded (Seeley et al., 2007; Bressler and Menon, 2010; Menon, 2011). Since the regulation and cognitive control-related terms were dominantly decoded, the mental processes of each love relationship might underlie the regulation and control mental processing interplayed by both the salience and cognitive control networks. The decoded results are shown in Table 2 and Supplementary Table 10. Additionally, given that the social norm-related terms such as “social norm” and “norm” were decoded, the activated brain regions might engage in social norm-related mental processes. Social norms are among the social decision-making and socially acceptable courtesy and manners (Sherif and Sherif, 1953) and they require rationally deliberative mental processes and thinking of the mental states of others (Berthoz et al., 2002; Bas-Hoogendam et al., 2017; Zinchenko and Arsalidou, 2018). The brain regions composing both the salience and cognitive control are associated with the social norms (Berthoz et al., 2002; Bas-Hoogendam et al., 2017; Zinchenko and Arsalidou, 2018). Thus, the interconnection of overlapping brain regions might engage in deliberative mental processing.

Regarding the distinctive brain regions and mental processes of the brand love relationships, core brain regions of the DMN such as the MPFC, medial part of the posterior cingulate gyrus, and parahippocampal gyrus were observed (Buckner et al., 2008; Andrews-Hanna, 2012). According to Andrews-Hanna (2012), the DMN is classified into three sub-systems composed of the core [the anterior MPFC (AMPFC) and medial part of the PCC], medial temporal lobe (< MTL >, the ventral MPFC < VMPFC >, the hippocampal formation and retrosplenial cortex < BA29, BA30 >), and dorsal MPFC sub-system (the DMPFC, temporal parietal function < TPJ >, lateral temporal cortex, and temporal pole). Both the core DMN and MTL subsystem were observed. The DMPFC and TPJ, which are components of the DMPFC subsystem, were observed. The core DMN is associated with self-referential processing, the MTL subsystem is associated with memory-based mental processes, and the DMPFC subsystem is associated with social cognition (Andrews-Hanna, 2012). The AMPFC, VMPFC, DMPFC, and the medial part of the PCC are referred as to the cortical midline structure (CMS) and are engaged in self-referential processing (Northoff and Bermpohl, 2004). Since the DMN and CMS-related terms were decoded, it could be speculated that these brain networks might engage in the mental processes of the brand love relationships in this stage. Although the social cognition-related terms (“social cognitive”) were decoded at a relatively higher rank and a similarity score of 0.65, self-referential processing-related terms (“self-referential”) were decoded in the top tiers at a higher score of 0.82 (see Supplementary Table 10 and Table 2). Interestingly, the mind-wandering related term “wandering” was decoded at a relatively higher rank with a similarity score of 0.61. Mind-wandering is one of the DMN-related mental, task-unrelated, and spontaneous thought processes (Buckner et al., 2008; Christoff et al., 2009, 2016; Andrews-Hanna, 2012; Fox et al., 2015). Since the interconnected activity between the salience network, cognitive control network, and the core DMN engages in mind-wandering (Christoff et al., 2009; Christoff, 2012), and the term “wandering” was decoded at the top tiers, the mental processes of the brand love relationships in this stage might be associated with mind-wandering. Given that, this internal mentation processing might be associated with frequent thinking (“very often have thoughts about it,” “coming to mind seemingly on their own”), which is one of the crucial constructs of brand love measurement (Park et al., 2010; Lastovicka and Sirianni, 2011; Batra et al., 2012; Bagozzi et al., 2017) and is a construct derived from self-brand integration (Batra et al., 2012). Thus, this consideration suggests that the mental processes of brand love relationships with weak social cognitive aspects might be strongly weighted by inward self-referential processing.

In the interpersonal romantic love relationships, the brain regions belonging to the salience network (i.e., the anterior insula and the dorsal part of the anterior cingulate gyrus) and to the cognitive control network (i.e., DMPFC, lateral sides of the frontal lobe, and the posterior part of the parietal regions) were mainly observed (Seeley et al., 2007; Menon and Uddin, 2010; Menon, 2011). Given that the brain regions related to these two networks were activated and the salience and cognitive control -related terms were decoded at the top tiers (see Table 2 and Supplementary Table 10), the activated brain regions might engage in regulation, control, and interoceptive processing (Seeley et al., 2007; Menon and Uddin, 2010; Menon, 2011). Since working memory- and emotional salience-related terms were well decoded. The working memory is the process which holds information for short periods of time to execute tasks (Baddeley, 2000). The working memory network includes the dorsal-lateral prefrontal cortex and posterior parietal regions, and is involved in working memory-related cognitive control processing such as planning, allocating attention, and inhibition (D’Esposito and Postle, 2015). Brain regions involved in both working memory and cognitive control networks overlap, and mental processes engaged in these networks are similar in their rational and deliberative nature (Menon, 2011). Both cognitive control and salience networks cooperatively function when executing emotional salience-relate tasks requiring cognitive resources to solve problems (Luo et al., 2014; Sheffield et al., 2021). Moreover, a connection between the salience and cognitive control network is enhanced when the task difficulties become high (Gong et al., 2016). “Flanker,” the most matched decoded term, is associated with the flanker task. The flanker task is helpful for assessing the mental processes involved in conflict resolution (Berron et al., 2015; Siemann et al., 2016). The cognitive control system is engaged in conflict resolution (Egner, 2008). According to Simpson et al. (2006), individuals with anxiety tend to distinguish relationship conflicts with beloved partners in daily life. In case these conflicts are severe and bring negative consequences for the stability of their future relationships, they try to implement conflict resolution strategies to preserve their relationships with their beloved partners (Pistole, 1989). The distinctive brain regions might engage in social decision-making since the decoded term “norm” is a social norm-related term, as previously described. Based on these considerations, the interplaying between the distinctive brain regions during the migration stage of the interpersonal romantic love relationships might strongly be engaged in social deliberative mental processing such as conflict resolution between partners.

### Stable stage

Regarding the common brain regions in the stable stage phase, both the ventral and dorsal parts of the anterior insula, striatum (from the ventral to dorsal regions), midbrain, and anterior thalamus were observed. The crucial function of the salience network anchored in the anterior insula is saliency detection (Seeley et al., 2007; Singer et al., 2009; Menon and Uddin, 2010; Uddin et al., 2014), which is required to integrate multiple processes such as emotion, reward, social cognition, somatosensation, interoception, and exteroception (Seeley et al., 2007; Craig, 2009; Singer et al., 2009; Menon and Uddin, 2010; Uddin et al., 2014). Since the results showed that control and regulation-related terms were not decoded while emotional salience-related terms were decoded, the major common mental processes of each love relationship might be visceral derived subjective feelings. The detailed decoded results are shown in Table 3 and Supplementary Table 11. Moreover, the dopamine-related term “dopamine” was commonly decoded at the top tiers across all the results of the conjunction and subtraction analysis concerning the stable stage phase. The activation of the anterior insula and dopamine activity is associated with saliency detection (Oei et al., 2012; Kutlu et al., 2013). Brain regions in the dopaminergic pathway were observed in the conjunction and subtraction analysis results. Brain regions in the mesolimbic dopamine pathway were overlappingly activated in both relationships, and distinctive brain regions involved in interpersonal romantic love relationships were also activated. The mesolimbic dopamine pathway is engaged in motivational and hedonic processes such as enjoyment and desire (Kelley et al., 2005; Alcaro et al., 2007; Blaess et al., 2020). The dopamine synthesis and release functions of the mesolimbic dopamine system are associated with the connectivity of nodes within the salience network (McCutcheon et al., 2019). The connectivity of brain network systems is involved in detecting incentive salience driven by motivation (Kesserwani, 2021). In brand love relationships, the dorsal striatum, which is a component of the nigrostriatal dopamine pathway, was activated. The nigrostriatal dopamine pathway plays a crucial role in regulating voluntary movement (de Oliveira-Souza, 2012; Jang and Cho, 2022) and reward (Wise, 2009). Additionally, the term “flexibility” was decoded as one of the most matched terms. This decoded result might demonstrate that “flexibility” is a construct derived from “cognitive flexibility” because the term “flexibility” was mainly composed of publications regarding cognitive flexibility studies (the publication list, which is queried through the NeuroQuery platform, is sited in the Supplementary Table 12. Cognitive flexibility is the ability to adapt to a changing environment and regulate the mental state to adapt to it (Scott, 1962). This ability is required to drive the dorsal frontal-striatal network (Kehagia et al., 2010; Langley et al., 2021; Uddin, 2021). Cognitive flexibility involves dopamine-related behaviors and the ability to execute goal-directed behavior (van der Meulen et al., 2007; Kehagia et al., 2010). Since the other most matched term was “making,” which is the decision-making related term, this suggests that both love relationships are a kind of goal-directed behavior. Thus, the common mental processes of both love relationships might lead to visceral-derived incentive saliency detection and attention processing when making decisions.

The stable stage-brand love is a phase in which relationships between brands and consumers are established. The putamen covered broader activated brain areas in the stable stage-brand love relationships than those in the other stage-brand love relationships. Given that the putamen is one of the components of the salience network (Seeley et al., 2007), connections between the anterior insula and putamen are involved in the interoceptive processing (Ainley et al., 2014; de la Fuente et al., 2019; Paik et al., 2019). Interoceptive awareness, a key element of interoceptive processing, is linked to automatic behavior (Ainley et al., 2014). Paik et al. (2019) demonstrated that interoceptive processing plays a role in uncontrollable behavior. The putamen is associated with habitual behaviors (Tricomi et al., 2009; Morris et al., 2016). The caudate, which is the shared brain region between brand love and interpersonal romantic love relationships, is associated with goal-directed behaviors (Grahn et al., 2008; Dong et al., 2020). Successive and gradual goal-directed behavior brings out a transformation from the dominance of the goal-directed system to the dominance of the habit system in a neural mechanism (Tricomi et al., 2009; Lee et al., 2014). When the habit system becomes dominant, the goal-directed system strengthens the habit system (Jackson et al., 2019). Given that both the putamen and caudate are composed of the habit network, this interplay between these systems leads to automatic behavior (Zilverstand et al., 2018). Since “salience,” “interoceptive,” and “habit” were also decoded, these cognitive functions may be involved in the mental processes of brand love relationships during this stage. Moreover, the term “flexibility” was decoded in the top tiers. Especially, the basal ganglia plays a crucial role in cognitive flexibility (Erickson et al., 2010; Verstynen et al., 2012; Becker et al., 2016; Jackson et al., 2019). According to Jackson et al. (2019), when the influences of the putamen in reversal learning are superior to those of the caudate, cognitive flexibility is reduced. This suggests that the putamen inhibits goal-directed behavior and leads to automatic behavior. Thus, distinctive mental processes of brand love relationships might be habit-based automatic processing. Most consumer behaviors during this stage might be executed unconsciously and without cognitive effort. This state based on the mental processes between the consumers and the brands can be interpreted as a close construct of action loyalty among the customer loyalty phases (Oliver, 1999). The action loyalty is the ultimate phase of customer loyalty, in which the consumers repurchase specific brands without cognitive efforts like seeking information about the brand (Oliver, 1999). It leads to inertial rebuying behavior and prevents consumers from switching brands. The dominant activation of the putamen in the stable stage-brand love might be associated with the prevention of switching from a current beloved brand to other competitors since the functions of the putamen modulate cognitive flexibility. Thus, the putamen might be the central brain region for customer loyalty. Since the decoded terms did not match well with the distinctive brain regions of the brand love relationships because of the lower similarity scores than the other stages, better-matched constructs than the currently interpreted constructs based on “cognitive flexibility” and “habit” might be associated with mental processing in brand love relationships during the stable stage.

The distinctive activated brain regions in the interpersonal romantic love relationships consists of two broad regions such as the ventral parts of the subcortical regions (the thalamus, ventral striatum, parahippocampal gyrus, midbrain) and BA13 including the ventral part of the anterior insula. The connected network between the ventral part of the anterior insula, sometimes along with the dorsal part of the anterior and the posterior part of the insula, and these sub-cortical regions is associated with reward processing such as reward-seeking and addiction (Naqvi and Bechara, 2009; Naqvi et al., 2014; Droutman et al., 2015; Zhao et al., 2022). These sub-cortical regions are components of the motivational network (Kelley et al., 2005). Given that the reward-related terms (“outcome,” and “reward”) and intensive motivation-related terms (“motivation” and “substance”) were decoded at the top tiers, the mental processes during the stable stage might be underlying the mental processes driven by the intensively motivated mental processing. While the mental processes of the brand love relationships are dominated by habit-based automatic mental processing without including the motivation-related mental processes, those of the interpersonal romantic love relationships are driven by the “wanting” system, in which each partner is seeking each other, despite the long-term relationship. This relationship state during the stable stage in interpersonal romantic love relationships is close to the results in the early stage. The mental processes in the early stage were also driven by the intensively motivational system such as reinforcement learning. This consideration is in line with the previous studies concerning long-term romantic love relationships (Acevedo and Aron, 2009; O’Leary et al., 2012). Acevedo and Aron (2009) demonstrated that romantic love did not decay in long-term relationships similar to the pattern observed in the relationships during the early stage. They also revealed that the long-term romantic love relationships might lead to the wellbeing of the individual (Acevedo and Aron, 2009). This differentiation of mental processes might be caused by the involvement of different dopamine systems and mental processes in each love relationship (interpersonal romantic love relationships: mesolimbic dopamine pathway; brand love relationships: nigrostriatal dopamine pathway). Moreover, the term “serotonin” was only decoded in interpersonal romantic love relationships, and not in brand love relationships. The density of serotonin transporters has an influence on the mental processes of infatuated couples such as obsessions with partners (Marazziti et al., 1999; Langeslag et al., 2012). Moreover, the serotonin transporter gene (5-HTTLPR) is associated with wellbeing (De Neve, 2011; Matsunaga et al., 2013). Parameters of relationship quality such as wellbeing during the stable stage could be associated with the function of 5-HTTLPR. This suggests that the brand love and interpersonal romantic love relationships during the stable stage might be different mental processes in terms of both relationship quality and motivation for lasting relationships. Since the brand love relationships might be a weaker bonded relationship than the interpersonal romantic love relationships in spite of existing in the stable stage, brand love relationships might have a vulnerable tie in comparison with interpersonal romantic love relationships. In other words, unlike the interpersonal romantic love relationships, consumers might not already consider a brand as a long-term partner with strong bonding at the stable stage because of a lack or weak intensive motivation to maintain lasting relationships.

### Evaluation of hypotheses

The assessment of each hypothesis mentioned in the Introduction section is described succeeding each hypothesis.

Hypothesis 1: If the neural mechanisms of brand love relationship dynamics are the same as those of interpersonal romantic love relationship dynamics, activation of the same brain regions should be observed, and the same constructs of the mental processes should be decoded across all stages.

Regarding hypothesis 1, although overlapping brain regions such as the anterior insula were activated across all stages, broadly different activated brain regions were observed across all stages. Different terms concerning each distinct brain region across all stages were also decoded. Additionally, many social cognitive constructs including prosocial aspects were decoded across all stages in the interpersonal romantic love relationships. However, only two decoded terms concerning social cognitive constructs were observed in brand love relationships but with no prosocial aspects (i.e., the term “trust” was decoded in the early stage, while “partner” was decoded in the stable stage.) Since it is difficult to support hypothesis 1, the brand love relationship dynamics and interpersonal romantic love relationship dynamics do not have identical neural mechanisms and have different mental processes.

Hypothesis 2: If relationships between consumers and brands are reinforced during term from the early to migration like interpersonal romantic love relationships, reinforcement learning-related brain regions should be observed as shared brain regions between brand love and interpersonal romantic love relationships in the early stage, and reinforcement learning-related constructs should be distinctively decoded in the early stage.

Regarding hypothesis 2, activation of the striatum (the putamen and caudate) was observed as common brain regions between the brand love and interpersonal romantic love relationships in the early stage. These regions are associated with reinforcement learning (Packard and Knowlton, 2002; Tanaka et al., 2007; Kunimatsu et al., 2019). Furthermore, reinforcement learning-related terms were decoded. Thus, the hypothesis 2 was confirmed. This suggests that both love relationships might have the same underlying mechanism in terms of both neural and mental processes systems during the early stage.

Hypothesis 3: If the function of motivation-related constructs, which is driven by intensive passionate emotion, in brand love relationship dynamics is weaker than that in interpersonal romantic love relationship dynamics, motivation-related brain regions should be weakly observed, and motivation-related constructs should be weakly decoded across all stages.

Regarding hypothesis 3, the connection between the anterior insula and the ventral striatum was observed in the early stage of both love relationships. This connection is associated with motivation-related constructs such as carving drugs (Goldstein et al., 2009; Koob and Volkow, 2010; Everitt and Robbins, 2016; Peters et al., 2016; Voon et al., 2020) as previously above. The connection was also observed in the interpersonal romantic love relationships but not in the brand love relationships at the stable stage. Moreover, in the stable stage of the interpersonal romantic love relationships, the brain regions composing the motivational network were activated. Regarding the shared brain regions in the early stage and distinctive brain regions in the stable stage of interpersonal romantic love relationships, motivation-related constructs were decoded. Regarding the distinctive brain regions in all stages of brand love relationships, motivation-related constructs were not decoded. This could interpret the magnitude of involving motivation-related constructs in brand love relationships during the early stage that might be weaker than that in interpersonal romantic love relationships. However, the one in the brand love relationships might not function during the stable stage. From the view of dynamics, the motivation-related constructs might be strongly involved in the mental processes of interpersonal romantic love relationships rather than that of brand love relationships. This suggests that the trajectories of both love relationship dynamics might be different. Thus, the hypothesis 3 was supported.

### General discussion

**FIGURE 9:**
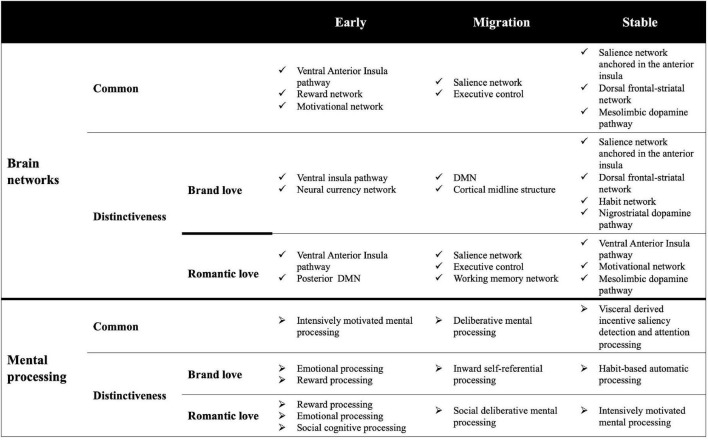
Brain networks and mental processing at each love relationship stage. DMN; default mode network.

Taken together, the many common mental processes, such as the intensive motivation, regulation, and saliency detection, derived from the overlapping brain regions with which the anterior insula has connections were observed across all three stages. Moreover, the present study revealed that the mental processes of both love relationships are driven by the early stage reinforcement learning system. However, different brain regions and brain networks identified in a wide range and distinct terms in each love relationship were decoded across all three stages. The major brain networks and mental processing at each stage of each love relationship are organized in Figure 9. Moreover, social cognitive aspects including prosocial aspects were strongly involved in the distinctive mental processes of interpersonal romantic love relationships across all stages. In contrast, no prosocial aspects were observed in the mental processes of brand love relationships in any stage; however, a few social cognitive aspects were observed. This is suggested to be the key difference between the brand love relationships and interpersonal romantic love relationships. It is also suggested to be the key factor in constructing strong bonded relationships in interpersonal romantic love relationships but not in the brand love relationships. The interplay between the common and distinct brain networks in both love relationships might develop different trajectories of mental processing in each love relationship.

### Limitations and future research

Although the present study provides useful findings to practitioners and academicians, there are several limitations in our study. Since we conducted an ALE analysis without considering various product categories, the present results are integrated brain activation areas regarding brand love relationships, which do not include differentiations of consumer behavior depending on product categories. This indicates that our results have universalities because of depending on comprehensive publications regarding brand love studies with a brain activation method. However, no distinctiveness and characteristics depending on variations in product categories were assessed in this study. Since these differences are not considered in the present results, the present results have limitations for application to marketing strategies. Moreover, the first ALE was conducted by small experiments. The experiments in most studies conducting the first ALE, as recommended by Eickhoff et al. (2016), did not meet the appropriate experiment size (more than 17 experiments). While, according to Bossier et al. (2018), an experiment size of approximately 10 is appropriate in terms of balancing of statistical error types (Type 1 and 2). Considering both criteria Eickhoff et al. (2016) and Bossier et al. (2018), the sample size for the first ALE may have been insufficient. Regarding the classification of relationship stages, several studies may be classified under another stage. For example, the experimental stimulus used in Deppe et al. (2005) was a well-known magazine logo. In the present study, that study was classified into the early stage based on weighting of criteria such as the strength of the relationship between brands and consumers. However, when weighting criteria such as relationship length, that study might be classified as being in the stable stage. Thus, depending on the perspective of the classifying studies, the allocation of studies to stages might be altered. One possible approach for addressing this limitation is to conduct an analysis using all possible combinations of the studies, from which stages can be designated. Results obtained using these procedures may have sufficient robustness. The present study offers a perspective for addressing this issue. There are limitations to interpreting decoded results though we adopted the NeuroQuery platform. While the NeuroQuery approach has advantages in terms of the number of preserved terms for decoding analysis in comparison with the other decoding methods such as the Neurosynth and BrainMap platforms, it has critical weakness in terms of statistical robustness. Unlike the decoding method with the BrainMap platform, decoded results using the NeuroQuery platform are not the results thresholded by statistical methods. In this study, although we adopted the NeuroQuery approach with the emphasis on the number of terms for decoding functions of brain regions, in order to obtain more robust results, assessments using both NeuroQuery and BrainMap may be required in the next study. Moreover, due to the insufficient samples in the studies, the decline stage in both love relationships could not be assessed in this study. However, given that this is the first triggered study for meta-analytical connectivity of brain regions regarding brand love relationships, it may provide useful indications for subsequent brand love studies using neuroscientific approaches. For example, researchers can refer to the present results as the regions of interest for brain regions related to brand love relationships when conducting an experimental study with brain imaging techniques such as fMRI. Although the present study provides useful information, there are still many limitations to address. Further studies are needed to address these issues and further research is required for precisely characterizing the neural basis of the dynamics of brand love relationships.

## Conclusion

To our knowledge, this is the first study revealing the neural mechanisms of the brand love relationship dynamics. The present study concluded that the developmental trajectories between brand love and interpersonal romantic love relationships are underlaid in different neural mechanisms and innately different mental processes generated by interconnecting both common and distinctive brain networks at each stage. This finding is generally consistent with those of previous brand love studies (Langner et al., 2015, 2016; Bagozzi et al., 2017). Brand love relationships may be more vulnerable due to underlying mental processes associated with weak social aspects, without the prosocial aspects of interpersonal romantic love relationships. For researchers, these findings suggest that crude applications of interpersonal romantic love theories to brand love relationship studies should be avoided. Practitioners need to realize that the brand love relationships are vulnerable on the premise when managing brand love relationships. Even though strong brand love relationships have been established and reached the stable stage, brand managers must carefully monitor signs of changing relationships between brands and customers by investigating indices of the relationship strength such as the brand love measurement (Batra et al., 2012) and methods of measuring intensive love feelings (Langner et al., 2016; Gumparthi et al., 2021), Therefore, nurturing brand love requires considerable attention and commitment.

## Data availability statement

The original contributions presented in this study are included in the article/Supplementary material, further inquiries can be directed to the corresponding author.

## Author contributions

SW: conceptualization, data curation, formal analysis, funding acquisition, investigation, methodology, project administration, resources, software, supervision, validation, visualization, writing—original draft, and writing—review and editing.

## Footnotes

1
http://www.brainmap.org/

2
http://ric.uthscsa.edu/mango/

3
https://www.brainmap.org/sleuth/

4
https://neuroquery.org/

5
https://github.com/neuroquery/neuroquery

6
https://neurosynth.org/
