# Supplementary material for: Neural mechanisms of brand love relationship dynamics: Is the development of brand love relationships the same as that of interpersonal romantic love relationships?
Source: Front Neurosci. 2022 Nov 10;16:984647. doi: 10.3389/fnins.2022.984647 (PMC9686448; doi:10.3389/fnins.2022.984647)
Supplement: Supplementary file 1 [file Data_Sheet_1.docx]

Supplementary Material

# Supplementary Tables

# Table S1(A) Early stage of brand love relationship studies included in the meta- analysis.

| **Studies** | **Subject** | **Foci** | **Contrast / Correlation** | **Experiment stimuli** | **Detailed information** |
| --- | --- | --- | --- | --- | --- |
| Erk et al.(2002) | 12 | 7 | Sports cars > Small cars | Package images | Cultural objects (cars; sports car/ limousine /small cars)  -Major car category |
|  |  | 2 | Sports cars > Limousines |  |  |
|  |  | 2 | Limousines > Sports cars |  |  |
|  |  | 2 | Limousines > Small cars |  |  |
| McClure et al.(2004) | 16 | 7 | Coke > Blinded drink | Brand logos/Carbonated drinks(Coke/Pepsi) | Culturally Familiar Drinks |
| Deppe et al.(2005a) | 21 | 5 | Correlations with credibility | Brand logos | Magazines |
| Schaefer et al.(2006) | 13 | 1 | Culturally familiar brand logos > Culturally unfamiliar brand logo | Brand logos  - Ferrarii, Volkswageni, Opeli, Mercedes-Benzi, Rolls-Roycei, BMWi, Porschei/ Buicki, Saturni, Holdeni, Pontiaci, Lincolni, Oldsmobilei, Acura | Culturally familiar brands（Cars) |
| Schaefer & Rotte(2007a) | 14 | 5 | Familiar brands > Unfamiliar brands | Brand logos | Cars |
|  |  | 1 | Sport/luxury brands > Unfamiliar brands |  |  |
|  |  | 5 | Value brands > Unfamiliar brands |  |  |
|  |  | 2 | Sports and luxury brands > Value brands |  |  |
| Klucharev et al. (2008) | 18 | 10 | Hits > Misses | Package images without brand logos | Clothes, cosmetics, packaged food, etc. |
|  | 18 | 7 | Experts > Non-experts |  |  |
|  | 16 | 4 | Celebrity expertise > Subsequent attitude effect |  |  |
|  | 18 | 4 | Celebrity expertise > Subsequent memory effect |  |  |
| Kato et al.(2009) | 40 | 18 | Unchanged > Changed  (After negative message) | Advertising with brand logos | Coke Ad/Pepsi Ad |
|  |  | 8 | Changed > Unchanged  (After negative message) |  |  |
|  |  | 3 | Unchanged > Changed  (After positive message) |  |  |
|  |  | 26 | Changed > Unchanged  (After positive message) |  |  |
| Casarotto et al.(2012) | 15 | 4 | Brand > IAPS | Brand logos | 13 differeint product categories(clothing, transport, food, gas, cigarettes, etc.) |
|  |  | 19 | Correlation with brand logo |  |  |
| Grabenhorst et al.(2013) | 13 | 2 | Health label > Taste label  (Health cost correlation) | Fooods with Taste label/Health label |  |
|  |  | 4 | Taste label > Health label  (Taste pleasantness correlation) |  |  |
|  |  | 2 | Health label > Taste label  (Correlation with absolute differences of health costs) |  |  |
|  |  | 2 | Taste label > Health label(Correlation with absolute differences of taste pleasantness) |  |  |
|  |  | 1 | Correlation with Confidence |  |  |
|  |  | 3 | Correlation with Cost-benefit |  |  |
|  |  | 4 | Health label > Taste label  (Psychophysiological interaction (PPI): seed area= amygdala) |  |  |
| Bruce et al.(2014) | 17 | 7 | Food logos > Baseline | Brand logos | foods(60):pizzahut,kfc,etc./non foods(60):lego,spongebob,windows,etc. |
|  |  | 5 | Non-food logos > Baseline |  |  |
|  |  | 4 | Food logos > Non-food logos |  |  |
| Burger and Stice(2014) | 9 | 10 | Coke intake > Milkshake intake | Product with logo | Coke Ad/Non food Ad (Not coke loyal customers) |
|  | 25 | 12 | Coke intake > tasteless solution intake | Product & logo ad |  |
|  |  | 37 | Anticipated Coke intake > Tasteless solution intake |  |  |
|  |  | 8 | Coke ads > Non-food ads |  |  |
|  |  | 11 | Coke product ads > Non-food ads |  |  |
| Chen et al(2015) | 17 | 23 | Correlation with brand personality | Brand logo | 44 brands listed by the intetrand brand ranking |
| Enax et al.(2015) | 40 | 9 | Fair Trade emblem > Conventional products | Food images with FT certified marks | Various food category (chocolate, coffee, rice, etc..) |
|  |  | 5 | Parametric modulation with Willing to pay |  |  |
|  |  | 7 | Categorical modulation with label |  |  |
|  |  | 10 | Correlating with the increment value (the “Willing to pay” difference between Fair and conventional products) |  |  |
|  |  | 4 | Correlation with Fair Trade pictures (PPI analysis: seed area = ventral striatum) |  |  |
|  |  | 4 | Correlation with Fair Trade pictures (PPI analysis: seed area = superior frontal gyrus) |  |  |
|  |  | 6 | Correlation with Fair Trade pictures (PPI analysis: seed area = anterior cingulate cortex) |  |  |
|  |  | 3 | Correlation with Fair Trade pictures (PPI analysis: seed area = posterior cingulate cortex) |  |  |
|  |  | 2 | Correlation with willing to pay |  |  |
| Jung et al.(2018) | 34 | 21 | Correlation with product decision | Food images with logo (social/conventional enterprises) | Confectionery(i.e., cookies, chocolate, bread, and Korean traditional rice cake) |
|  |  | 15 | Social > Non-social products |  |  |
|  |  | 9 | Non-social > Social products |  |  |
|  |  | 8 | Correlation with the behavioral index of ethical consumption |  |  |
|  |  | 11 | Social > Non-social  (PPI analysis: seed area= anterior cingulate cortex) |  |  |
|  |  | 4 | Social > Non-social  (PPI analysis: seed area= Dorsal medial prefrontal cortex) |  |  |
|  |  | 4 | Correlation with the model estimation error |  |  |
|  |  | 25 | Social > Non-social (price/decision events) |  |  |
|  |  | 1 | (Social vs. Non-social) X 2 (Observation > Control) Interaction |  |  |
|  |  | 21 | Social > Non-social (logo/item events, both groups combined) |  |  |

Foci section: The number of observed foci in each experiment.

**Reference**

Bruce, A. S., Bruce, J. M., Black, W. R., Lepping, R. J., Henry, J. M., Cherry, J. B. C., et al. (2014). Branding and a child’s brain: an fMRI study of neural responses to logos. *Social cognitive and affective neuroscience* 9, 118–122. doi: https://doi.org/10.1093/scan/nss109

Burger, K. S., and Stice, E. (2014). Neural responsivity during soft drink intake, anticipation, and advertisement exposure in habitually consuming youth. *Obesity* 22, 441–450.

Casarotto, S., Ricciardi, E., Romani, S., Dalli, D., and Pietrini, P. (2012). Covert brand recognition engages emotion-specific brain networks. *Archives italiennes de biol*ogie 150, 259–273. doi: 10.4449/aib.v150i4.1478

Chen, Y.-P., Nelson, L. D., and Hsu, M. (2015). From “where” to “what”: distributed representations of brand associations in the human brain. *Journal of Marketing Research* 52, 453–466. doi: http://dx.doi.org/10.1509/jmr.14.0606

Deppe, M., Schwindt, W., Kraemer, J., Kugel, H., Plassmann, H., Kenning, P., et al. (2005). Evidence for a neural correlate of a framing effect: Bias-specific activity in the ventromedial prefrontal cortex during credibility judgments. *Brain research bulletin* 67, 413–421. doi: 10.1016/j.brainresbull.2005.06.017

Enax, L., Krapp, V., Piehl, A., and Weber, B. (2015). Effects of social sustainability signaling on neural valuation signals and taste-experience of food products. *Frontiers in Behavioral Neuroscience* 9, 247. doi: https://doi.org/10.3389/fnbeh.2015.00247

Erk, S., Spitzer, M., Wunderlich, A. P., Galley, L., and Walter, H. (2002). Cultural objects modulate reward circuitry. *Neuroreport* 13, 2499–2503. doi: 10.1097/00001756-200212200-00024

Grabenhorst, F., Schulte, F. P., Maderwald, S., and Brand, M. (2013). Food labels promote healthy choices by a decision bias in the amygdala. *Neuroimage* 74, 152–163. doi: https://doi.org/10.1016/j.neuroimage.2013.02.012

Jung, D., Sul, S., Lee, M., and Kim, H. (2018). Social observation increases functional segregation between MPFC subregions predicting prosocial consumer decisions. *Scientific reports* 8, 1–13. doi: https://doi.org/10.1038/s41598-018-21449-z

Kato, J., Ide, H., Kabashima, I., Kadota, H., Takano, K., and Kansaku, K. (2009). Neural correlates of attitude change following positive and negative advertisements. *Frontiers in Behavioral Neuroscience* 3, 6. doi: https://doi.org/10.3389/neuro.08.006.2009

Klucharev, V., Smidts, A., and Fernández, G. (2008). Brain mechanisms of persuasion: how ‘expert power’modulates memory and attitudes. *Social cognitive and affective neuroscience* 3, 353–366. doi: https://doi.org/10.1093/scan/nsn022

McClure, S. M., Li, J., Tomlin, D., Cypert, K. S., Montague, L. M., and Montague, P. R. (2004). Neural correlates of behavioral preference for culturally familiar drinks. *Neuron* 44, 379–387. doi: https://doi.org/10.1016/j.neuron.2004.09.019

Schaefer, M., Berens, H., Heinze, H.-J., and Rotte, M. (2006). Neural correlates of culturally familiar brands of car manufacturers. *Neuroimage* 31, 861–865. doi: 10.1016/j.neuroimage.2005.12.047

Schaefer, M., and Rotte, M. (2007). Thinking on luxury or pragmatic brand products: Brain responses to different categories of culturally based brands. *Brain research* 1165, 98–104. doi: https://doi.org/10.1016/j.brainres.2007.06.038

**Table S1(B)**. **Migration stage of brand love relationship** **studies included in the meta- analysis.**

| **Studies** | **Subject** | **Foci** | **Contrast / Correlation** | **Experiment stimuli** | **Detailed information** |
| --- | --- | --- | --- | --- | --- |
| Deppe et al.(2005b) | 22 | 16 | Target > Diverse  (Male:First choice brand) | Package images with brand logos | Beer/Coffee |
|  |  | 2 | Target > Diverse  (Male: Non first choice brand) |  |  |
|  |  | 8 | Target > Diverse  (Female:First choice brand) |  |  |
|  |  | 11 | First choice brands > Non first choice brands |  |  |
| Koeneke et al.(2008) | 19 | 28 | Correlations with preference | Real products | Chochorate bars |
| Plassmann et al.(2008) | 20 | 8 | High > Low price  (Wine1:during sampling) | Wine | Intake |
|  |  | 10 | High > Low price  (Wine1:during swallowing) |  |  |
|  |  | 12 | High > Low price  (Wine2:during sampling) |  |  |
|  |  | 6 | High > Low price  (Wine2:during swallowing) |  |  |
|  |  | 2 | Wines 1 and 2 comparing activity at high vs. low price  (during sampling) |  |  |
|  |  | 5 | Wines 1 and 2 comparing activity at high vs. low price  (during swallowing) |  |  |
|  |  | 8 | interaction analysis for [$45-$5] -[$90-$10] |  |  |
|  |  | 1 | Interaction analysis for [$90-$10]-[$45-$5] |  |  |
|  |  | 2 | Correlation with liking ratings |  |  |
| Casarotto et al.(2012) | 15 | 1 | Correlation with brand favorability | Brand logos | 13 differeint product categories(clothing, transport, food, gas, cigarettes, etc.) |
| Esch et al.(2012) | 15 | 2 | Unfamiliar brands > Strong brands | Brand names and logo | 8 strong brands(BMW, Coca Cola, etc) and 8 weak brands(Kia, Yahoo, etc.)and 8 unfamiliar brands |
|  |  | 2 | Weak > Strong |  |  |
|  |  | 4 | Strong > Unfamiliar |  |  |
|  |  | 2 | Weak > Unfamiliar |  |  |
|  |  | 4 | Strong > Weak |  |  |
| Murawski et al.(2012) | 13 | 11 | Correlations with subjective value | Brand logos | Cups with Apple logo |
|  |  | 5 | Correlations with Apple logo |  |  |
|  |  | 2 | Apple > Neutral cup |  |  |
|  | 9 | 1 | Now > Later  (Decision outcome time delay) |  |  |
|  | 13 | 1 | Small serchlight analysis |  |  |
|  | 11 | 4 | Easy > Hard  (Decision difficulty) |  |  |
|  | 13 | 5 | Easy > Hard  (All time delay) |  |  |
| Audrin et al.(2017) | 38 | 20 | Most liked > Least liked * Luxurious > Non-luxurious | Items and brand logo images | Luxuarious and Non-luxuarious(scarves, handbags, belts and purses) |
|  |  | 26 | Least liked > Most liked * Luxurious > Non-luxurious |  |  |
|  |  | 2 | Region of interest analysis = caudate nucleus (Most liked > Least liked * Luxurious > Non-luxurious |  |  |
|  |  | 2 | Chosen > Not-chosen * Luxurious > Non-luxurious |  |  |

Foci section: The number of observed foci in each experiment.

**Reference**

Audrin, C., Ceravolo, L., Chanal, J., Brosch, T., and Sander, D. (2017). Associating a product with a luxury brand label modulates neural reward processing and favors choices in materialistic individuals. *Scientific reports* 7, 16176. doi: 10.1038/s41598-017-16544-6

Casarotto, S., Ricciardi, E., Romani, S., Dalli, D., and Pietrini, P. (2012). Covert brand recognition engages emotion-specific brain networks. *Archives italiennes de biologie* 150, 259–273. doi: 10.4449/aib.v150i4.1478

Deppe, M., Schwindt, W., Kugel, H., Plassmann, H., and Kenning, P. (2005). Nonlinear responses within the medial prefrontal cortex reveal when specific implicit information influences economic decision making. *Journal of Neuroimaging* 15, 171–182. doi: https://doi.org/10.1111/j.1552-6569.2005.tb00303.x

Esch, F. R., Möll, T., Schmitt, B., Elger, C. E., Neuhaus, C., and Weber, B. (2012). Brands on the brain: What happens neurophysiologically when consumers process and evaluate brands. *Journal of Consumer Psychology* 22, 75–85. doi: https://doi.org/10.1016/j.jcps.2010.08.004

Koeneke, S., Pedroni, A. F., Dieckmann, A., Bosch, V., and Jäncke, L. (2008). Individual preferences modulate incentive values: Evidence from functional MRI. *Behavioral and Brain Functions* 4, 55. doi: https://doi.org/10.1186/1744-9081-4-55

Murawski, C., Harris, P. G., Bode, S., and Egan, G. F. (2012). Led into temptation? Rewarding brand logos bias the neural encoding of incidental economic decisions. *PloS one* 7, e34155. doi: 10.1371/journal.pone.0034155

Plassmann, H., O’doherty, J., Shiv, B., and Rangel, A. (2008). Marketing actions can modulate neural representations of experienced pleasantness. *Proceedings of the National Academy of Sciences* 105, 1050–1054. doi: http://dx.doi.org/10.1073/pnas.0706929105

**Table S1(C)**. **Stable stage of brand love relationship** **studies included in the meta- analysis.**

| **Studies** | **Subject** | **Foci** |  | **Experiment stimuli** | **Detailed information** |
| --- | --- | --- | --- | --- | --- |
| Yoon et al.(2006) | 19 | 1 | Self-relevant Brand > Person | Brand names/Adjectivess | Various types of product category |
| Plassmann et al.(2007) | 22 | 1 | Brand with customer loyalty > Diverse brand | Brand logos(clothing images) | Departmant store |
| Schaefer & Rotte(2007b) | 13 | 4 | The most beloved/bonded brand > Non favorite brand | Brand logos | Cars |
| Schaefer et al.(2011) | 12 | 2 | The most favorite/attractive brand > Non favorite brand | Package images with brand logos | Chochorates |
| Reimann et al.(2011)  (Weber et al.(2007)) | 16 | 2 | Selling price > Buying price  (beloved musician & title condition) | Musician and title | Listen before experiments |
| Reimann et al.(2012) | 16 | 6 | Brand with close relationship > Neutral brand | Brand names and logo | Disney, Google, Apple and Starbucks Coffee. etc. |
| Burger and Stice(2014) | 25 | 4 | Habitual coke consumers > Non-consumers | Product & logo ad | Coke Ad/Non food Ad |

Foci section: The number of observed foci in each experiment.

**Reference**

Burger, K. S., and Stice, E. (2014). Neural responsivity during soft drink intake, anticipation, and advertisement exposure in habitually consuming youth. *Obesity* 22, 441–450

Plassmann, H., Kenning, P., and Ahlert, D. (2007). Why companies should make their customers happy: The neural correlates of customer loyalty. *Advances in Consumer Research* 34, 735–739

Reimann, M., Castaño, R., Zaichkowsky, J., and Bechara, A. (2012). How we relate to brands: Psychological and neurophysiological insights into consumer–brand relationships. *Journal of Consumer Psychology* 22, 128–142. doi: 10.1016/j.jcps.2011.11.003

Reimann, M., Schilke, O., Weber, B., Neuhaus, C., and Zaichkowsky, J. (2011). Functional magnetic resonance imaging in consumer research: A review and application. *Psychology & Marketing* 28, 608–637. doi: 10.1002/mar.20403

Schaefer, M., Knuth, M., and Rumpel, F. (2011). Striatal response to favorite brands as a function of neuroticism and extraversion. *Brain research* 1425, 83–89. doi: 10.1016/j.brainres.2011.09.055

Schaefer, M., and Rotte, M. (2007). Favorite brands as cultural objects modulate reward circuit. *Neuroreport* 18, 141–145. doi: https://doi.org/10.1097/wnr.0b013e328010ac84

Weber, B., Aholt, A., Neuhaus, C., Trautner, P., Elger, C. E., and Teichert, T. (2007). Neural evidence for reference-dependence in real-market-transactions. *Neuroimage* 35, 441–447. doi: https://doi.org/10.1016/j.neuroimage.2006.11.034

Yoon, C., Gutchess, A. H., Feinberg, F., and Polk, T. A. (2006). A functional magnetic resonance imaging study of neural dissociations between brand and person judgments. *Journal of Consumer Research* 33, 31–40. doi: http://dx.doi.org/10.1086/504132

**Table S2(A) Early stage of interpersonal romantic love relationship studies included in the meta- analysis.**

| **Studies** | **Subject** | **Foci** | **Contrast / Correlation** | **Experiment stimuli** | **Duration (participant condition/mean, month)** |
| --- | --- | --- | --- | --- | --- |
| Aron et al. (2005) | 17 | 8 | Beloved > familiar, neutral acquaintance | Pictures (face) / Passive viewing task | 7.4 |
|  |  | 9 | Correlation with the length of the relationships |  |  |
| Yin et al. (2018) | 32 | 14 | Romance level High > Low | Pictures  (romantic events) / Passive viewing task | 8.76 |
|  |  | 6 | Romance level Low > High |  |  |
|  |  | 13 | Male > Female |  |  |
|  |  | 24 | Male > Female (ROI analysis) |  |  |
| Xu et al. (2011) | 18 | 9 | Beloved > familiar, neutral acquaintance | Pictures (face) / Passive viewing task | 6.54 |
|  |  | 7 | Positive > Neutral (correlation with self-reported traditionality and modernity) |  |  |
| Langeslag et al. (2014) | 15 | 8 | Target > Distractor | Pictures (face) / Passive viewing task | 5.1 |
| Kim et al. (2009) | 10 | 13 | Beloved > Friend | Pictures (face) / Passive viewing task | < about 3(100days) |
|  |  | 16 | Beloved > Friend |  | 6 |
|  |  | 22 | Beloved(180days)>Beloved(100days) |  | < 6 |
| Stoessel et al. (2011) | 21 | 40 | Happy love > Unhappy love | Pictures (face) / Passive viewing task | < 6 |

Foci section: The number of observed foci in each experiment.

**Reference**

Aron, A., Fisher, H., Mashek, D. J., Strong, G., Li, H., and Brown, L. L. (2005). Reward, motivation, and emotion systems associated with early-stage intense romantic love. *Journal of neurophysiology* 94, 327–337. doi: https://doi.org/10.1152/jn.00838.2004

Kim, W., Kim, S., Jeong, J., Lee, K.-U., Ahn, K.-J., Chung, Y.-A., et al. (2009). Temporal changes in functional magnetic resonance imaging activation of heterosexual couples for visual stimuli of loved partners. *Psychiatry Investigation* 6, 19. doi: https://doi.org/10.4306%2Fpi.2009.6.1.19

Langeslag, S. J., van der Veen, F. M., and Röder, C. H. (2014). Attention modulates the dorsal striatum response to love stimuli. *Human Brain Mapping* 35, 503–512. doi: https://doi.org/10.1002/hbm.22197

Stoessel, C., Stiller, J., Bleich, S., Boensch, D., Doerfler, A., Garcia, M., et al. (2011). Differences and similarities on neuronal activities of people being happily and unhappily in love: a functional magnetic resonance imaging study. *Neuropsychobiology* 64, 52–60

Xu, X., Aron, A., Brown, L., Cao, G., Feng, T., and Weng, X. (2011). Reward and motivation systems: A brain mapping study of early-stage intense romantic love in Chinese participants. *Human brain mapping* 32, 249–257. doi: https://doi.org/10.1002/hbm.21017

Yin, J., Zou, Z., Song, H., Zhang, Z., Yang, B., and Huang, X. (2018). Cognition, emotion and reward networks associated with sex differences for romantic appraisals. *Scientific Reports* 8, 1–11. doi: https://doi.org/10.1038/s41598-018-21079-5

**Table S2(B) MIgration stage of interpersonal romantic love relationship studies included in the meta- analysis.**

| **Studies** | **Subject** | **Foci** | **Contrast / Correlation** | **Experiment stimuli** | **Duration (participant condition/mean, month)** |
| --- | --- | --- | --- | --- | --- |
| Bartels & Zeki (2000) | 17 | 13 | Beloved > Friend | Pictures (face) / Passive viewing task | 28.8 |
| Xu et al. (2012) | 18 | 2 | Beloved & Cigarette > Beloved & Pen | Pictures (face/cigarette/Pen) / Passive viewing task | 14.2 |
|  |  | 10 | - Beloved >Acquaintance |  |  |
| Song et al. (2015) | 66 | 45 | In love group > Single group | Resting state | 12.2 |
|  | 68 | 15 | In love group > Ended love group |  |  |
| Xu et al. (2011) | 18 | 5 | Beloved > familiar, neutral acquaintance | Pictures (face) / Passive viewing task | 18 |
| Scheele et al. (2013) | 20 | 15 | Beloved > Unfamiliar control  (Discovery study) | Pictures (face) / Passive viewing task | 28.75 |
|  |  | 10 | Beloved > Unfamiliar control  (Replication study) |  | 36.35 |

Foci section: The number of observed foci in each experiment.

**Reference**

Bartels, A., and Zeki, S. (2000). The neural basis of romantic love. *Neuroreport* 11, 3829–3834. doi: https://doi.org/10.1097/00001756-200011270-00046

Scheele, D., Wille, A., Kendrick, K. M., Stoffel-Wagner, B., Becker, B., Güntürkün, O., et al. (2013). Oxytocin enhances brain reward system responses in men viewing the face of their female partner. *Proceedings of the National Academy of Sciences* 110, 20308–20313

Song, H., Zou, Z., Kou, J., Liu, Y., Yang, L., Zilverstand, A., et al. (2015). Love-related changes in the brain: a resting-state functional magnetic resonance imaging study. *Frontiers in human neuroscience* 9, 71. doi: https://doi.org/10.3389/fnhum.2015.00071

Xu, X., Aron, A., Brown, L., Cao, G., Feng, T., and Weng, X. (2011). Reward and motivation systems: A brain mapping study of early-stage intense romantic love in Chinese participants. *Human brain mapping* 32, 249–257. doi: https://doi.org/10.1002/hbm.21017

Xu, X., Wang, J., Aron, A., Lei, W., Westmaas, J. L., and Weng, X. (2012). Intense passionate love attenuates cigarette cue-reactivity in nicotine-deprived smokers: An fMRI study. *PloS one* 7, e42235

**Table S2(C) MIgration stage of interpersonal romantic love relationship studies included in the meta- analysis.**

| **Studies** | **Subject** | **Foci** | **Contrast / Correlation** | **Experiment stimuli/task** | **Duration (participant condition/mean)** |
| --- | --- | --- | --- | --- | --- |
| Acevedo et al. (2012) | 17 | 30 | Partner > Highly Familiar acquaintance | Pictures (face) / Passive viewing task | 290.16  Months  (21.4 years) |
|  |  | 26 | Partner > Close friend |  |  |
|  |  | 9 | Partner and Close Friend > Familiar neutral |  |  |
|  |  | 24 | Correlation with relationship indices |  |  |
| Acevedo et al. (2020) | 19 | 2 | Correlations with romantic love maintenance | Pictures (face) / Passive viewing task | 49.32  Months |
|  |  | 5 | Interactions with AVPR1a rs3 (long alleles) and romantic love maintenance |  |  |
|  |  | 3 | Interactions with OXTR rs53576 (G alleles) and romantic love maintenance |  |  |
|  |  | 2 | Interactions with DRD4 7R alleles and romantic love maintenance |  |  |
| Xu et al. (2012) | 30 | 11 | Still beloved > Ended love | Pictures (face) / Passive viewing task | 40 Months |
|  |  | 3 | Correlation with relationship happiness after 40 months |  |  |
| Duarte et al. (2018) | 4 | 7 | Appraisal > Choice | Sentences about 38 activities/hobbies  (Battle of the sexes dilemma task) | 109.2 Months  (9.1 years) |
|  |  | 5 | Higher > Lower cooperation  (Appraisal condition) |  |  |
|  |  | 1 | Higher > Lower cooperation  (Choice condition) |  |  |
| Hamilton et al. (2017) | 20 | 8 | Romantic > Neutral | Pictures (neutral landscapes/ neutral with people/ romantic/ sexual) / Passive viewing task | No relationships duration described. Included long-term relationship partners |
|  |  | 11 | Romantic > Sexual |  |  |
| Brown et al. (2013) | 17 | 13 | Positive Partner > Highly-Familiar Neutral (Correlations with the Fisher Temperament Inventory Scores / long-term relationship group) | Pictures (face) | 256.8 |
|  | 18 | 13 | Positive Partner > Highly-Familiar Neutral (Correlations with the Fisher Temperament Inventory Scores / newlywed group) |  | 256.8 |
|  | 17 | 10 | Positive Partner > Highly-Familiar Neutral (Correlations with personality dimension scores / long-term relationship group) |  | 256.8 |
|  |  | 4 | Positive Partner > Highly-Familiar Neutral (Correlations with personality dimension scores / newlywed group) |  | 256.8 |

Foci section: The number of observed foci in each experiment.

**Reference**

Acevedo, B. P., Aron, A., Fisher, H. E., and Brown, L. L. (2012). Neural correlates of long-term intense romantic love. *Social cognitive and affective neuroscience* 7, 145–159. doi: https://doi.org/10.1093/scan/nsq092

Acevedo, B. P., Poulin, M. J., Collins, N. L., and Brown, L. L. (2020). After the honeymoon: neural and genetic correlates of romantic love in newlywed marriages. *Frontiers in psychology* 11, 634. doi: https://doi.org/10.3389/fpsyg.2020.00634

Brown, L. L., Acevedo, B., and Fisher, H. E. (2013). Neural correlates of four broad temperament dimensions: testing predictions for a novel construct of personality. *PloS one* 8, e78734

Duarte, I. C., Brito-Costa, S., Cayolla, R., and Castelo-Branco, M. (2018). The role of Prefrontal Cortex in a Battle of the Sexes Dilemma involving a Conflict between Tribal and Romantic love. *Scientific reports* 8, 1–8. doi: https://doi.org/10.1038/s41598-018-30611-6

Hamilton, L. D., and Meston, C. M. (2017). Differences in neural response to romantic stimuli in monogamous and non-monogamous men. *Archives of Sexual Behavior* 46, 2289–2299. doi: https://doi.org/10.1007/s10508-017-1071-9

Xu, X., Brown, L., Aron, A., Cao, G., Feng, T., Acevedo, B., et al. (2012). Regional brain activity during early-stage intense romantic love predicted relationship outcomes after 40 months: An fMRI assessment. *Neuroscience Letters* 526, 33–38. doi: 10.1016/j.neulet.2012.08.004

**Table S3(A). Results of the first ALE (Brand love relationships)**

| **Cluster #** | **Side** | **Brain region** | **BA** | **Peak voxel coordinates (MNI)** | | | **ALE**  **values** | **Cluster Size (mm3)** |
| --- | --- | --- | --- | --- | --- | --- | --- | --- |
|  |  |  |  | **x** | **y** | **z** |  |  |
| **(a) Early stage-brand love relationships** | | | | | | | | |
| 1 | L | Caudate Head (Ventral Striatum) | --- | -6 | 14 | -6 | 0.0262 | 1736 |
| 2 | R | Parahippocampal Gyrus | BA28 | 18 | -4 | -16 | 0.0323 | 1440 |
| 3 | L | Anterior Cingulate (ventral part / VMPFC) | BA32 | -4 | 40 | -14 | 0.0329 | 1160 |
| 4 | L | Anterior Cingulate (dorsal part / MPFC) | BA32 | -2 | 46 | 8 | 0.0236 | 1064 |
|  | L | Medial Frontal Gyrus (MPFC) | BA10 | 0 | 60 | 6 | 0.0172 |  |
|  | L | Medial Frontal Gyrus (MPFC) | BA10 | -10 | 56 | 10 | 0.0156 |  |
| 5 | R | Thalamus | --- | 14 | -16 | 6 | 0.0224 | 832 |
|  | R | Thalamus (Ventral Lateral Nucleus) | --- | 14 | -6 | 4 | 0.0174 |  |
| 6 | L | Lingual Gyrus | BA18 | -16 | -74 | -4 | 0.0274 | 824 |
| 7 | R | Lingual Gyrus | BA18 | 12 | -80 | 0 | 0.0220 | 680 |
|  | R | Lingual Gyrus | BA17 | 12 | -92 | 10 | 0.0187 |  |
| 8 | L | Lingual Gyrus | BA18 | -4 | -90 | 0 | 0.0221 | 512 |
| 9 | R | Postcentral Gyrus | BA2 | 40 | -24 | 42 | 0.0215 | 488 |
|  | R | Precentral Gyrus | BA4 | 48 | -16 | 36 | 0.0155 |  |
| 10 | L | Medial Frontal Gyrus (DMPFC) | BA8 | -4 | 38 | 38 | 0.0180 | 336 |
|  | L | Medial Frontal Gyrus (DMPFC) | BA8 | -8 | 46 | 36 | 0.0169 |  |
| 11 | R | Caudate Body | --- | 10 | 12 | 8 | 0.0209 | 296 |
| 12 | L | Medial Frontal Gyrus | BA6 | -8 | 12 | 56 | 0.0204 | 256 |
| 13 | L | Anterior Insula (dorsal part) | BA13 | -42 | 10 | 8 | 0.0189 | 208 |
| 14 | L | Cuneus | BA17 | -16 | -98 | 12 | 0.0187 | 192 |
| 15 | R | Cuneus | BA17 | 22 | -86 | 16 | 0.0189 | 192 |
| 16 | L | Amygdala | --- | -20 | -4 | -18 | 0.0175 | 136 |
| **(b) Migration stage-brand love relationships** | | | | | | | | |
| 1 | R | Medial Frontal Gyrus (MPFC) | BA10 | 10 | 54 | 0 | 0.0220 | 2032 |
|  | R | Anterior Cingulate (ventral part) | BA24 | 6 | 38 | -10 | 0.0167 |  |
| 2 | L | Medial Frontal Gyrus | BA10 | -4 | 46 | -18 | 0.0316 | 1288 |
| 3 | L | Cerebellum (Anterior Lobe) | --- | 2 | -40 | -34 | 0.0196 | 504 |
| 4 | R | Superior Temporal Gyrus | BA13 | 56 | -42 | 22 | 0.0145 | 376 |
| 5 | R | Posterior Cingulate | BA29 | 8 | -50 | 14 | 0.0148 | 368 |
| 6 | R | Middle Frontal Gyrus | BA10 | 36 | 52 | 18 | 0.0153 | 296 |
| 7 | L | Anterior Cingulate (ventral part / VMPFC) | BA24 | -10 | 36 | -10 | 0.0168 | 264 |
| 8 | R | Inferior Temporal Gyrus | BA21 | 62 | -8 | -22 | 0.0158 | 232 |
| 9 | R | Parahippocampal Gyrus | BA35 | 26 | -30 | -16 | 0.0142 | 208 |
| 10 | L | Caudate Head | --- | -8 | 8 | -2 | 0.0155 | 200 |
| 11 | R | Caudate Body | --- | 8 | 12 | 2 | 0.0154 | 192 |
| 12 | L | Medial Frontal Gyrus (DMPFC) | BA6 | -6 | 50 | 28 | 0.0148 | 184 |
| 13 | L | Posterior Cingulate | BA30 | -6 | -52 | 16 | 0.0145 | 176 |
| 14 | R | Caudate Body | --- | 16 | 0 | 24 | 0.0146 | 176 |
| 15 | R | Middle Frontal Gyrus | BA9 | 32 | 30 | 22 | 0.0145 | 168 |
| 16 | R | Cerebellum (Cerebellar Tonsil) | --- | 26 | -38 | -38 | 0.0134 | 160 |
| 17 | L | Medial Frontal Gyrus (MPFC) | BA9 | -10 | 48 | 10 | 0.0121 | 160 |
| 18 | L | Middle Temporal Gyrus | BA21 | -52 | 2 | -26 | 0.0142 | 152 |
| 19 |  | No Gray Matter found | --- | 4 | -22 | -22 | 0.0134 | 152 |
| 20 | R | Inferior Frontal Gyrus | BA45 | 50 | 24 | 10 | 0.0128 | 136 |
| 21 | L | Posterior Cingulate | BA29 | -6 | -42 | 6 | 0.0127 | 104 |
| **(c) Stable stage-brand love relationships** | | | | | | | | |
| 1 | R | Putamen | --- | 28 | 0 | 0 | 0.0079 | 312 |
|  | R | Putamen | --- | 24 | 6 | 6 | 0.0077 |  |
| 2 | L | Caudate Body | --- | -8 | 14 | 4 | 0.0083 | 160 |
| 3 | R | Posterior Cingulate | BA23 | 12 | -61 | 16 | 0.0086 | 160 |
| 4 | R | Posterior Cingulate | BA23 | 6 | -55 | 19 | 0.0083 | 160 |
| 5 | R | Precuneus | BA31 | 15 | -61 | 34 | 0.0083 | 160 |
| 6 | R | Caudate Head | --- | 22 | 30 | -2 | 0.0077 | 104 |
| 7 | L | Anterior Insula (dorsal part) | BA13 | -34 | 8 | 16 | 0.0077 | 104 |

BA, Brodmann Area; MNI, Montreal Neurological Institute; ALE, activation likelihood estimation; L, Left; R, Right; DMPFC, dorsal medial prefrontal cortex; MPFC, medial prefrontal cortex; VMPFC, ventral medial prefrontal cortex.

**Table S3(B). Results of the first ALE (Interpersonal romantic love relationships)**

| **Cluster #** | **Side** | **Brain region** | **BA** | **Peak voxel coordinates (MNI)** | | | **ALE**  **values** | **Cluster Size (mm3)** |
| --- | --- | --- | --- | --- | --- | --- | --- | --- |
|  |  |  |  | **x** | **y** | **z** |  |  |
| **(a) Early stage-interpersonal romantic love relationships** | | | | | | | | |
| 1 | L | Precuneus | BA7 | -8 | -76 | 44 | 0.0161 | 536 |
| 2 | L | Posterior Cingulate | BA30 | -14 | -54 | 18 | 0.0158 | 528 |
| 3 | L | Inferior Frontal Gyrus | BA10 | -42 | 54 | -10 | 0.0135 | 368 |
| 4 | L | Anterior Cingulate (dorsal part) | BA24 | 2 | 28 | 22 | 0.0141 | 368 |
| 5 | L | Cuneus | BA17 | -6 | -92 | 16 | 0.0126 | 336 |
| 6 | L | Superior Frontal Gyrus | BA8 | -20 | 44 | 40 | 0.0144 | 240 |
| 7 | R | Caudate Head (Ventral Striatum) | --- | 8 | 10 | -12 | 0.0134 | 232 |
| 8 | L | Midbrain (Mammillary Body) | --- | 2 | -14 | -12 | 0.0132 | 176 |
| 9 | L | Caudate Body | --- | -12 | 12 | 18 | 0.0120 | 176 |
| 10 | R | Anterior Cingulate (ventral part / VMPFC) | BA32 | 4 | 50 | -10 | 0.0109 | 112 |
| **(b) Migration stage-interpersonal romantic love relationships** | | | | | | | | |
| 1 | L | Anterior Insula (dorsal part) | BA13 | -46 | 6 | 2 | 0.0180 | 744 |
|  | L | Claustrum | --- | -38 | 4 | 0 | 0.0133 |  |
| 2 | L | Fusiform Gyrus | BA37 | -44 | -62 | -8 | 0.0151 | 416 |
| 3 | R | Precuneus | BA7 | 6 | -60 | 50 | 0.0185 | 360 |
| 4 | L | Anterior Cingulate (dorsal part / MPFC) | BA24 | 0 | 36 | 8 | 0.0128 | 312 |
| 5 | R | Cingulate Gyrus | BA32 | 8 | 30 | 40 | 0.0133 | 256 |
| 6 | R | Superior Frontal Gyrus | BA8 | 6 | 28 | 50 | 0.0139 | 232 |
| 7 | R | Precuneus | BA7 | 30 | -48 | 54 | 0.0114 | 192 |
| 7 | R | Inferior Parietal Lobule | BA40 | 36 | -48 | 48 | 0.0110 |  |
| 8 | R | Anterior Insula (ventral part) | BA13 | 48 | 10 | -8 | 0.0126 | 184 |
| 9 | L | Putamen | --- | -24 | 2 | 20 | 0.0120 | 136 |
| 10 | L | Lingual Gyrus | BA17 | -21 | -90 | 2 | 0.0111 | 128 |
| 11 |  | No Gray Matter found | --- | 38 | -72 | 24 | 0.0111 | 128 |
| **(c) Stable stage-interpersonal romantic love relationships** | | | | | | | | |
| 1 | R | Midbrain (Subthalamic Nucleus) | --- | 16 | -18 | -10 | 0.0252 | 4784 |
|  | R | Midbrain (Mammillary Body) | --- | 4 | -16 | -16 | 0.0240 |  |
|  | R | Medial Globus Pallidus | --- | 10 | 4 | -6 | 0.0234 |  |
|  | L | Thalamus | --- | 2 | -6 | -6 | 0.0220 |  |
|  |  | No Gray Matter found | --- | 2 | -32 | -22 | 0.0218 |  |
|  |  | No Gray Matter found | --- | 2 | -28 | -20 | 0.0211 |  |
|  | L | Thalamus | --- | 2 | -18 | 0 | 0.0196 |  |
| 2 | R | Caudate Tail | --- | 36 | -34 | 0 | 0.0229 | 640 |
|  | R | Hippocampus | --- | 38 | -30 | -8 | 0.0151 |  |
| 3 | R | Middle Frontal Gyrus | BA46 | 42 | 48 | -2 | 0.0211 | 496 |
| 4 | L | Cuneus | BA23 | -14 | -72 | 16 | 0.0210 | 376 |
| 5 | R | Medial Frontal Gyrus (VMPFC) | BA10 | 2 | 58 | -10 | 0.0238 | 336 |
| 6 | L | Hippocampus | --- | -34 | -32 | -4 | 0.0222 | 328 |
| 7 | R | Claustrum | --- | 40 | 12 | -4 | 0.0196 | 280 |
| 8 | L | Mid Insula | BA13 | -44 | 4 | 0 | 0.0175 | 168 |
| 9 | L | Putamen | --- | -22 | 2 | 6 | 0.0167 | 136 |
| 10 | L | Putamen | --- | -20 | 2 | 16 | 0.0167 | 136 |
| 11 | R | Medial Frontal Gyrus (DMPFC) | BA9 | 26 | 46 | 18 | 0.0154 | 136 |

BA, Brodmann Area; MNI, Montreal Neurological Institute; ALE, activation likelihood estimation; L, Left; R, Right; DMPFC, dorsal medial prefrontal cortex; MPFC, medial prefrontal cortex; VMPFC, ventral medial prefrontal cortex.

**Table S4(A). Results of MACM (Brand love relationships)**

| **(a) Early stage-brand love relationships** | | | | | | | | |
| --- | --- | --- | --- | --- | --- | --- | --- | --- |
| **Cluster #** | **Side** | **Brain region** | **BA** | **Peak voxel coordinates (MNI)** | | | **ALE**  **values** | **Cluster Size (mm3)** |
|  |  |  |  | **x** | **y** | **z** |  |  |
| 1 | R | Amygdala | --- | 20 | -4 | -16 | 0.3481 | 94840 |
|  | R | Thalamus | --- | 12 | -14 | 4 | 0.2682 |  |
|  | L | Caudate Head | --- | -10 | 14 | -4 | 0.2463 |  |
|  | L | Amygdala | --- | -20 | -4 | -16 | 0.1934 |  |
|  | L | Thalamus | --- | -10 | -14 | 4 | 0.1612 |  |
|  | R | Caudate Head | --- | 12 | 14 | -4 | 0.1467 |  |
|  | R | Anterior Insula (ventral part) | BA13 | 36 | 24 | -6 | 0.1288 |  |
|  | L | Anterior Insula | BA13 | -34 | 20 | 0 | 0.1088 |  |
|  | R | Inferior Frontal Gyrus | BA9 | 48 | 8 | 26 | 0.0845 |  |
|  | L | Inferior Frontal Gyrus | BA47 | -42 | 22 | -12 | 0.0830 |  |
|  | L | Midbrain (Red Nucleus) | --- | -6 | -20 | -10 | 0.0728 |  |
|  | L | Middle Frontal Gyrus | BA46 | -42 | 34 | 12 | 0.0702 |  |
|  | L | Parahippocampal Gyrus | BA28 | -22 | -20 | -12 | 0.0689 |  |
|  | L | Anterior Insula | BA13 | 50 | 16 | 0 | 0.0685 |  |
|  | L | Inferior Frontal Gyrus | BA47 | -34 | 30 | -14 | 0.0685 |  |
|  | R | Inferior Frontal Gyrus | BA45 | 52 | 30 | 6 | 0.0533 |  |
|  | R | Middle Frontal Gyrus | BA46 | 52 | 28 | 18 | 0.0511 |  |
|  | L | Mid Insula | BA13 | -38 | 4 | 6 | 0.0466 |  |
|  | R | Caudate Body | --- | 16 | 2 | 16 | 0.0457 |  |
|  | L | Precentral Gyrus | BA44 | -54 | 6 | 8 | 0.0438 |  |
| 2 | L | Anterior Cingulate (ventral part / VMPFC) | BA32 | -4 | 42 | -14 | 0.2001 | 17320 |
|  | L | Anterior Cingulate (dorsal part) | BA32 | -2 | 46 | 8 | 0.1350 |  |
|  | L | Medial Frontal Gyrus (DMPFC) | BA9 | -4 | 54 | 16 | 0.0699 |  |
|  | R | Medial Frontal Gyrus (DMPFC) | BA9 | 6 | 54 | 24 | 0.0522 |  |
|  | L | Superior Frontal Gyrus (DMPFC) | BA9 | -8 | 54 | 30 | 0.0488 |  |
| 3 | L | Cingulate Gyrus | BA24 | 2 | 6 | 46 | 0.0858 | 11384 |
|  | R | Medial Frontal Gyrus | BA6 | 4 | 2 | 56 | 0.0782 |  |
|  | R | Cingulate Gyrus | BA32 | 6 | 22 | 30 | 0.0718 |  |
|  | L | Superior Frontal Gyrus | BA6 | -4 | 14 | 52 | 0.0677 |  |
|  | L | Cingulate Gyrus | BA32 | -6 | 26 | 26 | 0.0492 |  |
|  | R | Medial Frontal Gyrus | BA8 | -6 | 24 | 44 | 0.0443 |  |
| 4 | R | Inferior Temporal Gyrus | BA32 | 50 | -70 | -2 | 0.0789 | 4424 |
|  | R | Fusiform Gyrus | BA32 | 42 | -52 | -18 | 0.0709 |  |
| **(b) Migration stage-brand love relationships** | | | | | | | | |
| 1 | L | Anterior Cingulate (ventral part) | BA32 | -2 | 46 | -16 | 0.2359 | 22904 |
|  | R | Anterior Cingulate | BA32 | 8 | 52 | -2 | 0.1332 |  |
|  | R | Anterior Cingulate (ventral part) | BA24 | 6 | 38 | -10 | 0.1033 |  |
|  | L | Anterior Cingulate (dorsal part) | BA32 | 0 | 40 | 6 | 0.0576 |  |
|  | L | Medial Frontal Gyrus (MPFC) | BA10 | -6 | 56 | 0 | 0.0490 |  |
|  | L | Superior Frontal Gyrus (DMPFC) | BA9 | -2 | 56 | 26 | 0.0441 |  |
|  | L | Medial Frontal Gyrus (DMPFC) | BA9 | 2 | 54 | 26 | 0.0436 |  |
|  | L | Superior Frontal Gyrus (DMPFC) | BA8 | -8 | 54 | 32 | 0.0432 |  |
|  | L | Medial Frontal Gyrus (MPFC) | BA9 | -4 | 50 | 12 | 0.0384 |  |
| 2 | L | Posterior Cingulate | BA23 | -6 | -48 | 30 | 0.0704 | 6096 |
|  | L | Precuneus | BA31 | 0 | -54 | 36 | 0.0637 |  |
|  | R | Posterior Cingulate | BA29 | 8 | -48 | 14 | 0.0451 |  |
|  | L | Posterior Cingulate | BA23 | -4 | -62 | 22 | 0.0397 |  |
| 3 | R | Superior Temporal Gyrus | BA13 | 56 | -44 | 22 | 0.1139 | 5072 |
|  | R | Middle Temporal Gyrus | BA39 | 58 | -60 | 26 | 0.0404 |  |
| 4 | L | Amygdala | --- | -20 | -8 | -18 | 0.0840 | 4376 |
|  | L | Amygdala | --- | -28 | -2 | -22 | 0.0461 |  |
| 5 | R | Amygdala | --- | 22 | -6 | -18 | 0.0724 | 3224 |
|  | R | Parahippocampal Gyrus | BA28 | 26 | -22 | -14 | 0.0358 |  |
| 6 | R | Anterior Insula (dorsal part) | BA13 | -34 | 18 | 4 | 0.0457 | 3184 |
|  | L | Inferior Frontal Gyrus | BA47 | -30 | 30 | -18 | 0.0414 |  |
|  | L | Inferior Frontal Gyrus | BA47 | -46 | 28 | -8 | 0.0409 |  |
|  | L | Inferior Frontal Gyrus | BA47 | -40 | 22 | -14 | 0.0408 |  |
|  | L | Inferior Frontal Gyrus | BA47 | -40 | 28 | -16 | 0.0388 |  |
| 7 | R | Middle Frontal Gyrus | BA10 | 36 | 52 | 18 | 0.0756 | 2184 |
| 8 | R | Middle Frontal Gyrus | BA9 | 46 | 20 | 24 | 0.0492 | 2184 |
|  | R | Inferior Frontal Gyrus | BA9 | 46 | 10 | 30 | 0.0346 |  |
|  | R | Precentral Gyrus | BA6 | 42 | 4 | 36 | 0.0292 |  |
|  | R | Precentral Gyrus | BA6 | 42 | 2 | 40 | 0.0285 |  |
| **(c) Stable stage-brand love relationships** | | | | | | | | |
| 1 | L | Caudate Body |  | -6 | 14 | 4 | 0.0478 | 8256 |
|  | L | Putamen |  | -24 | -2 | 6 | 0.0419 |  |
|  | L | Thalamus |  | -12 | -16 | 2 | 0.0309 |  |
|  | L | Anterior Insula (dorsal part) | BA13 | -34 | 8 | 12 | 0.0241 |  |
| 2 | R | Putamen |  | 26 | 2 | 2 | 0.1445 | 6808 |
|  | R | Thalamus (Ventral Lateral Nucleus) |  | 14 | -14 | 2 | 0.0289 |  |
| 3 | L | Medial Frontal Gyrus | BA6 | -2 | 0 | 62 | 0.0407 | 4168 |
|  | L | Cingulate Gyrus | BA24 | -6 | 2 | 44 | 0.0207 |  |
| 4 | L | Precentral Gyrus | BA4 | -52 | -8 | 44 | 0.0335 | 3312 |
|  | L | Precentral Gyrus | BA6 | -46 | 4 | 30 | 0.0263 |  |
| 5 | L | Anterior Insula (ventral part) | BA13 | -44 | 10 | -4 | 0.0377 | 2816 |
| 6 | R | Precentral Gyrus | BA44 | 50 | 14 | 2 | 0.0331 | 2736 |
|  | R | Inferior Frontal Gyrus | BA44 | 58 | 12 | 10 | 0.0286 |  |
| 7 | R | Precentral Gyrus | BA6 | 54 | -6 | 32 | 0.0291 | 2312 |
|  | R | Precentral Gyrus | BA6 | 46 | -8 | 36 | 0.0251 |  |
|  | R | Precentral Gyrus | BA4 | 54 | -2 | 46 | 0.0249 |  |
| 8 | L | Anterior Insula (dorsal part) | BA13 | -34 | 24 | 4 | 0.0370 | 1272 |

MACM, Meta-Analytical Connectivity Modeling; BA, Brodmann Area; MNI, Montreal Neurological Institute; ALE, activation likelihood estimation; L, Left; R, Right; DMPFC, dorsal medial prefrontal cortex; MPFC, medial prefrontal cortex; VMPFC, ventral medial prefrontal cortex.

**Table S4(B). Results of MACM (Romantic love relationships)**

| **(a) Early stage-romantic love relationships** | | | | | | | | |
| --- | --- | --- | --- | --- | --- | --- | --- | --- |
| **Cluster #** | **Side** | **Brain region** | **BA** | **Peak voxel coordinates (MNI)** | | | **ALE**  **values** | **Cluster Size (mm3)** |
|  |  |  |  | **x** | **y** | **z** |  |  |
| 1 | R | Caudate Head (Ventral Striatum) | --- | 8 | 10 | -10 | 0.0964 | 8184 |
|  | L | Sub-lobar | --- | -6 | 6 | -12 | 0.0324 |  |
|  | L | Putamen (Ventral Striatum) | --- | -12 | 12 | -8 | 0.0293 |  |
|  | R | Amygdala | --- | 22 | -4 | -22 | 0.0262 |  |
|  | L | Caudate Head (Ventral Striatum) | --- | -4 | 16 | -6 | 0.0231 |  |
| 2 | L | Posterior Cingulate | BA30 | -14 | -54 | 16 | 0.0702 | 2280 |
| 3 | L | Precuneus | BA7 | -8 | -76 | 44 | 0.0634 | 2032 |
| 4 | L | Inferior Frontal Gyrus | BA47 | -46 | 20 | -8 | 0.0271 | 1448 |
|  | L | Claustrum | --- | -30 | 22 | 2 | 0.0192 |  |
| 5 | L | Midbrain(Mammillary Body) | --- | 2 | -12 | -12 | 0.0403 | 1296 |
| 6 | R | Anterior Insula (ventral part) | BA13 | 44 | 16 | -8 | 0.0279 | 1192 |
|  | R | Anterior Insula (ventral part) | BA47 | 38 | 20 | -8 | 0.0234 |  |
|  | R | Extra-Nuclear | BA13 | 36 | 18 | -18 | 0.0191 |  |
| **(b) Migration stage- romantic love relationships** | | | | | | | | |
| 1 | L | Claustrum | --- | 34 | 22 | -2 | 0.0894 | 23528 |
|  | R | Anterior Insula (ventral part) | BA13 | 48 | 10 | -6 | 0.0874 |  |
|  | R | Thalamus | --- | 12 | -12 | 4 | 0.0656 |  |
|  | R | Lentiform Nucleus | --- | 16 | 2 | 2 | 0.0478 |  |
|  | R | Caudate Head | --- | 12 | 10 | -2 | 0.0445 |  |
|  | R | Medial Globus Pallidus | --- | 14 | -4 | -6 | 0.0422 |  |
|  | R | Putamen | --- | 28 | 2 | -6 | 0.0417 |  |
|  | L | Thalamus | --- | -10 | -14 | 4 | 0.0400 |  |
|  | R | Claustrum | --- | 32 | 12 | -12 | 0.0368 |  |
|  | R | Thalamus | --- | 8 | -22 | -4 | 0.0331 |  |
|  | L | Midbrain (Red Nucleus) | --- | -6 | -22 | -10 | 0.0322 |  |
|  | L | Thalamus | --- | -4 | -24 | 4 | 0.0321 |  |
|  | L | Midbrain (Red Nucleus) | --- | -4 | -24 | -4 | 0.0305 |  |
| 2 | R | Cingulate Gyrus | BA32 | 6 | 30 | 40 | 0.1049 | 19840 |
|  | R | Cingulate Gyrus | BA32 | 6 | 20 | 36 | 0.0731 |  |
|  | L | Anterior Cingulate (dorsal part / MPFC) | BA24 | 0 | 36 | 8 | 0.0685 |  |
|  | L | Cingulate Gyrus | BA24 | -2 | 10 | 48 | 0.0572 |  |
|  | R | Medial Frontal Gyrus | BA6 | 0 | 4 | 62 | 0.0468 |  |
|  | R | Anterior Cingulate (dorsal part) | BA32 | 8 | 32 | 22 | 0.0349 |  |
| 3 | L | Anterior Insula (dorsal part) | BA13 | -46 | 6 | 2 | 0.1325 | 14160 |
|  | L | Anterior Insula | BA13 | -34 | 22 | 0 | 0.0715 |  |
|  | L | Lateral Globus Pallidus | --- | -20 | -2 | -4 | 0.0473 |  |
| 4 | R | Inferior Frontal Gyrus | BA9 | 48 | 8 | 28 | 0.0753 | 10592 |
|  | R | Precentral Gyrus | BA6 | 44 | 2 | 34 | 0.0585 |  |
|  | R | Middle Frontal Gyrus | BA9 | 38 | 44 | 22 | 0.0462 |  |
|  | R | Middle Frontal Gyrus | BA9 | 42 | 34 | 34 | 0.0431 |  |
|  | R | Inferior Frontal Gyrus | BA9 | 50 | 18 | 22 | 0.0410 |  |
|  | R | Middle Frontal Gyrus | BA9 | 48 | 32 | 24 | 0.0409 |  |
|  | R | Middle Frontal Gyrus | BA46 | 48 | 36 | 10 | 0.0377 |  |
| 5 | L | Precentral Gyrus | BA6 | -42 | 0 | 34 | 0.0528 | 7928 |
|  | L | Precentral Gyrus | BA6 | -42 | 4 | 42 | 0.0487 |  |
|  | L | Inferior Frontal Gyrus | BA9 | -44 | 8 | 26 | 0.0482 |  |
|  | L | Sub-Gyral | BA6 | -28 | 4 | 60 | 0.0333 |  |
|  | L | Middle Frontal Gyrus | BA6 | -34 | 4 | 56 | 0.0329 |  |
|  | L | Precentral Gyrus | BA6 | -42 | 0 | 34 | 0.0528 |  |
| 6 | R | Superior Parietal Lobule | BA7 | 34 | -48 | 50 | 0.0943 | 6776 |
|  | R | Inferior Parietal Lobule | BA40 | 46 | -50 | 38 | 0.0439 |  |
|  | R | Precuneus | BA7 | 24 | -66 | 50 | 0.0342 |  |
| 7 | L | Fusiform Gyrus | BA37 | -44 | -62 | -8 | 0.1413 | 5232 |
| 8 | R | Fusiform Gyrus | BA37 | 48 | -64 | -6 | 0.0626 | 3960 |
|  | R | Fusiform Gyrus | BA37 | 42 | -50 | -16 | 0.0375 |  |
|  | R | Cerebellum (Culmen) | --- | 30 | -62 | -22 | 0.0373 |  |
| 9 | R | Precuneus | BA7 | 6 | -60 | 50 | 0.1123 | 3560 |
|  | L | Precuneus | BA7 | -6 | -64 | 54 | 0.0409 |  |
| 10 | L | Inferior Parietal Lobule | BA40 | -40 | -52 | 48 | 0.0606 | 3152 |
| **(c) Stable stage- romantic love relationships** | | | | | | | | |
| 1 | R | Caudate Head (Ventral Striatum) | --- | 10 | 6 | -6 | 0.4043 | 101128 |
|  | R | Midbrain (Mammillary Body) | --- | 4 | -14 | -12 | 0.2277 |  |
|  | L | Lateral Globus Pallidus | --- | -12 | 8 | -4 | 0.2177 |  |
|  |  | No Gray Matter found | --- | 4 | -26 | -20 | 0.2147 |  |
|  | R | Midbrain (Substania Nigra) | --- | 16 | -18 | -12 | 0.2071 |  |
|  | L | Thalamus | --- | 2 | -18 | 0 | 0.1925 |  |
|  | L | Claustrum | --- | -30 | 22 | -2 | 0.1477 |  |
|  | R | Claustrum | --- | 40 | 10 | -8 | 0.1368 |  |
|  | R | Claustrum | --- | 34 | 22 | -2 | 0.1290 |  |
|  | L | Amygdala | --- | -22 | -4 | -18 | 0.0916 |  |
|  | L | Medial Frontal Gyrus | BA10 | 0 | 58 | -10 | 0.0799 |  |
|  | L | Inferior Frontal Gyrus | BA47 | -26 | 30 | -14 | 0.0777 |  |
|  | R | Anterior Cingulate (ventral part) | BA24 | 4 | 32 | -10 | 0.0755 |  |
|  | R | Anterior Insula (ventral part) | BA47 | 28 | 26 | -14 | 0.0711 |  |
|  | R | Middle Frontal Gyrus | BA46 | 50 | 38 | 14 | 0.0647 |  |
|  | R | Parahippocampal Gyrus | BA36 | 34 | -18 | -28 | 0.0638 |  |
|  | R | Hippocampus | --- | 32 | -36 | 0 | 0.0633 |  |
|  | R | Hippocampus | --- | 30 | -22 | -18 | 0.0550 |  |
|  | L | Putamen | --- | -28 | -4 | 0 | 0.0534 |  |
|  | L | Thalamus (Pulvinar) | --- | -12 | -28 | 8 | 0.0507 |  |
|  | R | Superior Temporal Gyrus | BA22 | 56 | 8 | -4 | 0.0505 |  |
|  | L | Thalamus | --- | -14 | -30 | 0 | 0.0503 |  |
| 2 | L | Anterior Cingulate (dorsal part) | BA24 | 0 | 38 | 6 | 0.1070 | 19360 |
|  | R | Cingulate Gyrus | BA32 | 6 | 16 | 40 | 0.0991 |  |
|  | R | Cingulate Gyrus | BA32 | 6 | 24 | 30 | 0.0858 |  |
|  | R | Anterior Cingulate (dorsal part) | BA32 | 4 | 38 | 16 | 0.0802 |  |
|  | L | Cingulate Gyrus | BA24 | -2 | 18 | 30 | 0.0778 |  |
|  | L | Anterior Cingulate (dorsal part) | BA24 | 2 | 30 | 20 | 0.0685 |  |
|  | L | Medial Frontal Gyrus | BA6 | 2 | 4 | 62 | 0.0645 |  |
|  | L | Medial Frontal Gyrus | BA9 | -2 | 38 | 30 | 0.0626 |  |
|  | R | Medial Frontal Gyrus | BA6 | 6 | 8 | 60 | 0.0620 |  |
|  | R | Caudate Body | --- | 14 | 2 | 24 | 0.0603 |  |
| 3 | L | Fusiform Gyrus | BA37 | -42 | -62 | -12 | 0.0768 | 4168 |
|  | L | Fusiform Gyrus | BA37 | -44 | -52 | -18 | 0.0737 |  |
|  | L | Middle Temporal Gyrus | BA37 | -52 | -58 | 6 | 0.0645 |  |

MACM, Meta-Analytical Connectivity Modeling; BA, Brodmann Area; MNI, Montreal Neurological Institute; ALE, activation likelihood estimation; L, Left; R, Right; MPFC, medial prefrontal cortex.

**Table S5. Results of the conjunction analysis (Brand love and Romantic love)**

| **Cluster #** | **Side** | **Brain region** | **BA** | **Peak voxel coordinates (MNI)** | | | **ALE**  **values** | **Cluster Size (mm3)** |
| --- | --- | --- | --- | --- | --- | --- | --- | --- |
|  |  |  |  | **x** | **y** | **z** |  |  |
| **Early-stage phase** | | | | | | | | |
| 1 | R | Caudate Head (Ventral Striatum) | --- | 8 | 12 | -10 | 0.0879 | 6248 |
|  | L | Sub-lobar (Ventral Striatum) | --- | -6 | 6 | -12 | 0.0324 |  |
|  | L | Putamen (Ventral Striatum) | --- | -12 | 12 | -8 | 0.0293 |  |
|  | R | Amygdala | --- | 22 | -4 | -22 | 0.0262 |  |
|  | R | Caudate Head (Ventral Striatum) | --- | -4 | 16 | -6 | 0.0231 |  |
| 2 | L | Inferior Frontal Gyrus | BA47 | -46 | 20 | -8 | 0.0271 | 1440 |
|  | L | Claustrum | --- | -30 | 22 | 2 | 0.0192 |  |
| 3 | R | Anterior Insula (ventral part) | BA13 | 44 | 16 | -8 | 0.0279 | 864 |
|  | R | Anterior Insula (ventral part) | B47 | 38 | 20 | -8 | 0.0234 |  |
| 4 | L | Midbrain (Mammillary Body) | --- | 0 | -14 | -12 | 0.0389 | 624 |
| 5 | R | Midbrain (Mammillary Body) | --- | 6 | -12 | -8 | 0.0217 | 24 |
| 6 | R | Thalamus | --- | 4 | -14 | -6 | 0.0183 | 8 |
| **Migration-stage phase** | | | | | | | | |
| 1 | L | Anterior Insula (dorsal part) | BA13 | -34 | 18 | 4 | 0.0457 | 1344 |
|  | L | Inferior Frontal Gyrus | BA47 | -38 | 20 | -12 | 0.0358 |  |
|  | L | Anterior Insula (ventral part) | BA13 | -40 | 24 | -4 | 0.0314 |  |
| 2 | R | Middle Frontal Gyrus | BA9 | 48 | 18 | 22 | 0.0404 | 1320 |
|  | R | Middle Frontal Gyrus | BA9 | 46 | 16 | 28 | 0.0397 |  |
|  | R | Inferior Frontal Gyrus | BA9 | 46 | 10 | 30 | 0.0346 |  |
|  | R | Middle Frontal Gyrus | BA9 | 48 | 24 | 28 | 0.0305 |  |
|  | R | Precentral Gyrus | BA6 | 42 | 4 | 36 | 0.0292 |  |
|  | R | Precentral Gyrus | BA6 | 42 | 2 | 40 | 0.0285 |  |
| 3 | R | Middle Frontal Gyrus | BA10 | 36 | 46 | 20 | 0.0403 | 888 |
| 4 | L | Anterior Cingulate (dorsal part / MPFC) | BA24 | 0 | 38 | 6 | 0.0514 | 432 |
| 5 | L | Lateral Globus Pallidus | --- | -18 | -2 | -12 | 0.0369 | 320 |
| **Stable-stage phase** | | | | | | | | |
| 1 | L | Caudate Body | --- | -6 | 14 | 4 | 0.0478 | 4496 |
|  | L | Putamen | --- | -22 | 2 | 4 | 0.0414 |  |
|  | L | Thalamus | --- | -12 | -16 | 2 | 0.0309 |  |
| 2 | R | Putamen | --- | 22 | 6 | 2 | 0.0912 | 4296 |
|  | R | Thalamus (Ventral Lateral Nucleus) | --- | 14 | -14 | 2 | 0.0289 |  |
| 3 | L | Anterior Insula (ventral part) | BA13 | -44 | 10 | -4 | 0.0377 | 2344 |
| 4 | L | Medial Frontal Gyrus | BA6 | -2 | 0 | 62 | 0.0407 | 1968 |
|  | L | Cingulate Gyrus | BA24 | -6 | 2 | 44 | 0.0207 |  |
| 5 | L | Anterior Insula (dorsal part) | BA13 | -34 | 24 | 4 | 0.0370 | 1248 |
| 6 | R | Precentral Gyrus | BA44 | 46 | 8 | 2 | 0.0249 | 592 |
|  | R | Anterior Insula (ventral part) | BA13 | 48 | 14 | -2 | 0.0245 |  |
| 7 | L | Anterior Insula (dorsal part) | BA13 | -34 | 10 | 10 | 0.0218 | 128 |
| 8 | L | Thalamus | --- | -26 | 12 | 6 | 0.0168 | 8 |

BA, Brodmann Area; MNI, Montreal Neurological Institute; ALE, activation likelihood estimation; L, Left; R, Right; MPFC, medial prefrontal cortex.

**Table S6. Results of the subtraction analysis (Early-stage phase)**

| **Cluster #** | **Side** | **Brain region** | **BA** | **Peak voxel coordinates (MNI)** | | | **Z values** | **Cluster Size (mm3)** |
| --- | --- | --- | --- | --- | --- | --- | --- | --- |
|  |  |  |  | **x** | **y** | **z** |  |  |
| **Brand love > Romantic love** | | | | | | | | |
| 1 | L | Medial Globus Pallidus (Ventral Striatum) | --- | -16 | -7 | -8 | 3.891 | 4408 |
|  | L | Putamen (Ventral Striatum) | --- | -25 | -7 | -13 | 3.291 |  |
|  | L | Thalamus (Medial Dorsal Nucleus) | --- | -4 | -12 | 4 | 3.291 |  |
|  | L | Midbrain (Substania Nigra) | --- | -14 | -24 | -14 | 2.848 |  |
| 2 | R | Parahippocampal Gyrus  (Entorhinal Cortex) | BA28 | 18 | -5 | -16 | 3.891 | 3184 |
| 3 | R | Thalamus (Ventral Lateral Nucleus) | --- | 13 | -14 | 1 | 3.891 | 2736 |
|  | R | Thalamus | --- | 10 | -6 | 4 | 3.353 |  |
| 4 | L | Caudate Body | --- | -10 | 17 | 1 | 3.353 | 2056 |
|  | L | Caudate Body | --- | -12 | 14 | 8 | 3.121 |  |
| 5 | L | Medial Frontal Gyrus (MPFC) | BA9 | 2 | 56 | 8 | 3.891 | 1496 |
|  | L | Medial Frontal Gyrus (MPFC) | BA9 | -7 | 45 | 14 | 2.894 |  |
| 6 | L | Anterior Cingulate (VMPFC) | BA32 | -6 | 42 | -10 | 3.891 | 1088 |
|  | L | Anterior Cingulate (VMPFC) | BA32 | -6 | 48 | -12 | 3.156 |  |
| 7 | R | Precentral Gyrus | BA6 | 40 | 8 | 28 | 3.353 | 360 |
| 8 | R | Cerebellum (Culmen) | --- | 42 | -48 | -22 | 2.782 | 296 |
| 9 | R | Claustrum | --- | 28 | 22 | 0 | 3.036 | 240 |
| 10 | R | Inferior Occipital Gyrus | BA19 | 46 | -72 | -2 | 2.506 | 144 |
| 11 | R | Putamen | --- | 22 | 18 | -2 | 2.989 | 120 |
| **Brand love < Romantic love** | | | | | | | | |
| 1 | R | Caudate Head (Ventral Striatum) | --- | 7 | 10 | -13 | 3.891 | 2728 |
|  | R | Putamen (Ventral Striatum) | --- | 17 | 12 | -16 | 3.540 |  |
| 2 | L | Precuneus | BA7 | -8 | -75 | 46 | 3.891 | 2024 |
| 3 | L | Posterior Cingulate | BA30 | -15 | -55 | 17 | 3.891 | 1808 |
| 4 | R | Midbrain (Mammillary Body) | --- | 3 | -12 | -10 | 3.891 | 608 |
| 5 | L | Medial Frontal Gyrus | BA6 | -4 | -12 | 52 | 3.291 | 128 |
| 6 | R | Anterior Insula (ventral part) | BA13 | 44 | 14 | -10 | 2.697 | 120 |

BA, Brodmann Area; MNI, Montreal Neurological Institute; ALE, activation likelihood estimation; L, Left; R, Right; MPFC, medial prefrontal cortex; VMPFC, ventral medial prefrontal cortex.

**Table S7. Results of the subtraction analysis (Migration-stage phase)**

| **Cluster #** | **Side** | **Brain region** | **BA** | **Peak voxel coordinates (MNI)** | | | **Z values** | **Cluster Size (mm3)** |
| --- | --- | --- | --- | --- | --- | --- | --- | --- |
|  |  |  |  | **x** | **y** | **z** |  |  |
| **Brand love > Romantic love** | | | | | | | | |
| 1 | R | Anterior Cingulate (VMPFC) | BA32 | 2 | 46 | -9 | 3.891 | 19776 |
| 2 | L | Cingulate Gyrus | BA31 | 0 | -54 | 33 | 3.891 | 5176 |
|  | L | Precuneus | B31 | -11 | -45 | 30 | 3.719 |  |
|  | R | Posterior Cingulate | BA29 | 10 | -50 | 8 | 2.834 |  |
|  | R | Posterior Cingulate | BA29 | 8 | -46 | 16 | 2.820 |  |
|  | R | Posterior Cingulate | BA29 | 10 | -46 | 10 | 2.807 |  |
| 3 | R | Superior Temporal Gyrus | BA22 | 54 | -54 | 22 | 3.891 | 3216 |
|  | R | Inferior Parietal Lobule | B40 | 56 | -43 | 26 | 3.719 |  |
| 4 | L | Amygdala | --- | -26 | -7 | -20 | 3.891 | 1520 |
|  | L | Hippocampus | --- | -28 | -18 | -22 | 3.353 |  |
| 5 | R | Hippocampus | --- | 28 | -18 | -16 | 3.540 | 400 |
| 6 | R | Inferior Frontal Gyrus | BA45 | 54 | 32 | -2 | 2.661 | 248 |
|  | R | Inferior Frontal Gyrus | BA45 | 50 | 34 | -4 | 2.652 |  |
| 7 | R | Parahippocampal Gyrus | BA34 | 22 | 0 | -18 | 2.597 | 160 |
| 8 | L | Inferior Frontal Gyrus | BA47 | -46 | 30 | -10 | 2.727 | 128 |
| **Brand love < Romantic love** | | | | | | | | |
| 1 | L | Cingulate Gyrus | BA32 | 3 | 24 | 45 | 3.891 | 11296 |
|  | R | Medial Frontal Gyrus | BA6 | 7 | 21 | 45 | 3.891 |  |
|  | L | Medial Frontal Gyrus | BA6 | 2 | 2 | 64 | 3.540 |  |
|  | L | Anterior Cingulate (dorsal part) | BA24 | 2 | 26 | 22 | 2.968 |  |
|  | L | Cingulate Gyrus | BA32 | -4 | 28 | 30 | 2.727 |  |
| 2 | R | Anterior Insula (ventral part) | BA13 | 48 | 12 | -7 | 3.891 | 6936 |
|  | R | Anterior Insula (dorsal part) | BA13 | 39 | 15 | 6 | 3.036 |  |
|  | R | Anterior Insula (ventral part) | BA13 | 34 | 24 | -2 | 3.239 |  |
|  | R | Inferior Frontal Gyrus | BA13 | 42 | 24 | 4 | 3.156 |  |
| 3 | L | Anterior Insula (dorsal part) | BA13 | -42 | 6 | 1 | 3.891 | 6712 |
|  | L | Inferior Frontal Gyrus | BA13 | -38 | 24 | 5 | 3.011 |  |
|  | L | Anterior Insula (ventral part) | BA13 | -32 | 26 | -2 | 2.878 |  |
| 4 | L | Fusiform Gyrus | BA37 | -44 | -62 | -8 | 3.891 | 4488 |
| 5 | R | Precuneus | BA7 | 34 | -46 | 50 | 3.891 | 3528 |
|  | R | Inferior Parietal Lobule | BA40 | 48 | -50 | 38 | 3.011 |  |
| 6 | R | Thalamus (Ventral Anterior Nucleus) | --- | 13 | -6 | 2 | 3.891 | 3400 |
|  | R | Thalamus (Ventral Anterior Nucleus) | --- | 15 | -19 | 7 | 3.540 |  |
|  | R | Thalamus (Ventral Anterior Nucleus) | --- | 13 | -13 | 3 | 3.432 |  |
|  | R | Thalamus | --- | 14 | -11 | 13 | 3.291 |  |
| 7 | L | Precentral Gyrus | BA6 | -46 | 3 | 47 | 3.891 | 3000 |
|  | L | Precentral Gyrus | BA6 | -43 | 2 | 43 | 3.719 |  |
| 8 | R | Precuneus | BA7 | 5 | -60 | 50 | 3.891 | 2528 |
| 9 | R | Fusiform Gyrus | BA37 | 52 | -63 | -8 | 3.891 | 1648 |
|  | R | Fusiform Gyrus | BA37 | 44 | -64 | -8 | 3.719 |  |
| 10 | R | Middle Frontal Gyrus | BA10 | 42 | 42 | 16 | 3.891 | 1552 |
|  | R | Middle Frontal Gyrus | BA9 | 44 | 39 | 23 | 2.452 |  |
|  | R | Middle Frontal Gyrus | BA9 | 50 | 36 | 22 | 2.948 |  |
|  | R | Middle Frontal Gyrus | BA9 | 50 | 34 | 28 | 2.929 |  |
| 11 | L | Inferior Parietal Lobule | BA40 | -40 | -52 | 44 | 3.432 | 1304 |
|  | L | Inferior Parietal Lobule | BA40 | -44 | -48 | 44 | 3.432 |  |
| 12 | R | Precentral Gyrus | BA6 | 48 | 2 | 27 | 3.891 | 1176 |
|  | R | Precentral Gyrus | BA6 | 52 | 2 | 28 | 3.719 |  |
| 13 | L | Lateral Globus Pallidus | --- | -20 | -4 | -4 | 3.432 | 688 |
| 14 | L | Sub-Gyral | BA6 | -27 | 8 | 58 | 3.719 | 496 |
| 15 | R | Putamen | --- | 30 | 0 | -2 | 2.968 | 216 |
| 16 | L | Thalamus | --- | -12 | -10 | 0 | 3.011 | 216 |
| 17 | L | Anterior Cingulate (dorsal part) | BA24 | 2 | 32 | 8 | 2.644 | 168 |
|  | L | Anterior Cingulate (dorsal part) | BA24 | 0 | 30 | 14 | 2.562 |  |
| 18 | R | Precuneus | BA7 | 22 | -64 | 54 | 2.748 | 112 |

BA, Brodmann Area; MNI, Montreal Neurological Institute; ALE, activation likelihood estimation; L, Left; R, Right; VMPFC, ventral medial prefrontal cortex.

**Table S8. Results of the subtraction analysis (Stable-stage phase)**

| **Cluster #** | **Side** | **Brain region** | **BA** | **Peak voxel coordinates (MNI)** | | | **Z values** | **Cluster Size (mm3)** |
| --- | --- | --- | --- | --- | --- | --- | --- | --- |
|  |  |  |  | **x** | **y** | **z** |  |  |
| **Brand love > Romantic love** | | | | | | | | |
| 1 | L | Putamen | --- | -21 | 2 | 7 | 3.540 | 4856 |
|  | L | Claustrum | --- | -30 | 6 | 12 | 3.432 |  |
| 2 | R | Putamen | --- | 26 | 2 | 4 | 3.891 | 4368 |
|  | R | Thalamus | --- | 16 | -12 | 0 | 2.807 |  |
|  | R | Thalamus (Ventral Posterior Lateral Nucleus) | --- | 18 | -17 | 2 | 2.782 |  |
| 3 | L | Medial Frontal Gyrus | BA6 | -1 | -2 | 63 | 3.891 | 2696 |
|  | R | Medial Frontal Gyrus | BA6 | 10 | 0 | 60 | 3.540 |  |
|  | R | Medial Frontal Gyrus | BA6 | 4 | 0 | 53 | 3.432 |  |
| 4 | L | Precentral Gyrus | BA4 | -52 | -8 | 40 | 3.891 | 2664 |
| 5 | R | Precentral Gyrus | BA6 | 52 | -6 | 34 | 3.891 | 2224 |
|  | R | Precentral Gyrus | BA4 | 58 | -3 | 42 | 3.719 |  |
| 6 | R | Precentral Gyrus | BA44 | 52 | 14 | 6 | 3.719 | 2160 |
|  | R | Precentral Gyrus | BA44 | 49 | 12 | 2 | 3.432 |  |
|  | R | Anterior Insula (dorsal part) | BA13 | 42 | 18 | 8 | 3.121 |  |
|  | R | Precentral Gyrus | BA6 | 60 | 6 | 8 | 2.989 |  |
| 7 | L | Anterior Insula (ventral part) | BA13 | -47 | 10 | -4 | 3.353 | 1032 |
|  | L | Anterior Insula (dorsal part) | BA13 | -48 | 8 | 2 | 3.062 |  |
| **Brand love < Romantic love** | | | | | | | | |
| 1 | R | Thalamus | --- | 3 | -7 | -7 | 3.891 | 19352 |
|  | R | Midbrain (Red Nucleus) | --- | 5 | -18 | -17 | 3.891 |  |
|  | R | Parahippocampal Gyrus | BA35 | 25 | -21 | -14 | 3.540 |  |
|  | R | Hippocaumpus | --- | 30 | -20 | -16 | 3.432 |  |
| 2 | R | Inferior Frontal Gyrus | BA47 | 30 | 18 | -20 | 3.719 | 1152 |
|  | R | Inferior Frontal Gyrus | BA47 | 36 | 22 | -20 | 3.353 |  |
|  | R | Extra-Nuclear | BA13 | 34 | 16 | -14 | 3.239 |  |
| 3 | L | Anterior Insula (ventral part) | BA13 | -30 | 21 | -12 | 2.894 | 240 |

BA, Brodmann Area; MNI, Montreal Neurological Institute; ALE, activation likelihood estimation; L, Left; R, Right.

**Table S9. Decoded results in early-stage phase (Including anatomical and uninterpretable terms)**

| **Brand love**  **and**  **Interpersonal romantic love** | | **Brand love**  **>**  **Interpersonal romantic love** | | **Brand love**  **<**  **Interpersonal romantic love** | |
| --- | --- | --- | --- | --- | --- |
| Term | Similarity | Term | Similarity | Term | Similarity |
| ventral striatum | 1 | relevant | 1 | self | 1 |
| outcome | 0.96 | affect | 0.99 | precuneus | 0.94 |
| ventral | 0.95 | avoid | 0.94 | positive | 0.78 |
| reward | 0.91 | amygdala | 0.93 | theory | 0.73 |
| motivation | 0.9 | emotional | 0.9 | immediate | 0.68 |
| striatum | 0.9 | arousal | 0.9 | attribution | 0.66 |
| gain | 0.89 | negative | 0.9 | mentalizing | 0.65 |
| substance | 0.88 | limbic | 0.89 | outcome | 0.65 |
| collection | 0.83 | trust | 0.89 | failure | 0.65 |
| monetary | 0.82 | adaptive | 0.87 | ventral striatum | 0.64 |
| positive | 0.8 | fear | 0.87 | sad | 0.63 |
| rewarding | 0.79 | response | 0.84 | trial | 0.62 |
| reward processing | 0.79 | avoidance | 0.83 | positive negative | 0.62 |
| signaling | 0.79 | salient | 0.83 | mind | 0.61 |
| accumbens | 0.79 | research | 0.82 | collection | 0.61 |
| nucleus accumbens | 0.78 | self report | 0.81 | reward | 0.61 |
| reinforcement | 0.78 | nucleus | 0.81 | thinking | 0.6 |
| unexpected | 0.78 | psychiatric | 0.8 | choose | 0.59 |
| immediate | 0.78 | reactivity | 0.8 | unexpected | 0.59 |
| loss | 0.77 | healthy | 0.8 | substance | 0.59 |
| seeking | 0.76 | work | 0.8 | personality traits | 0.58 |
| money | 0.76 | emotional stimuli | 0.79 | success | 0.58 |
| incentive | 0.76 | receptor | 0.79 | bulimia | 0.58 |
| punishment | 0.75 | affective | 0.78 | moral | 0.57 |
| losses | 0.74 | robust | 0.78 | goal | 0.57 |
| consumption | 0.73 | connectivity | 0.78 | gain | 0.57 |
| prediction error | 0.71 | subcortical structures | 0.78 | motivation | 0.56 |
| personality traits | 0.71 | negative affect | 0.77 | ventral | 0.56 |
| alcohol abuse | 0.71 | withdrawal | 0.77 | parent | 0.56 |
| behavior | 0.71 | anxiety | 0.76 | eating disorder | 0.55 |
| dorsal striatum | 0.71 | never | 0.76 | bulimia nervosa | 0.55 |
| tegmental | 0.7 | signaling | 0.76 | monetary | 0.54 |
| monetary reward | 0.7 | aversive | 0.76 | goal directed | 0.54 |
| substance abuse | 0.69 | anxiety disorder | 0.75 | alzheimer disease | 0.54 |
| expected | 0.69 | hypothalamus | 0.75 | depressive symptom | 0.53 |
| striatal | 0.69 | habituation | 0.73 | alzheimer | 0.53 |
| gambling | 0.69 | amygdala response | 0.73 | value | 0.53 |
| mesolimbic | 0.69 | x | 0.71 | anorexia | 0.53 |
| ventral tegmental area | 0.69 | persistent | 0.71 | nervosa | 0.52 |
| positive negative | 0.69 | neutral stimuli | 0.71 | substance use disorder | 0.52 |
| nucleus | 0.68 | stress | 0.71 | reward processing | 0.52 |
| withdrawal | 0.68 | engagement | 0.71 | rewarding | 0.52 |
| reception | 0.68 | fearful | 0.71 | midline | 0.52 |
| self report | 0.68 | vein | 0.7 | seeking | 0.52 |
| da | 0.67 | hyper | 0.7 | accumbens | 0.51 |
| desired | 0.67 | thalamus | 0.7 | esteem | 0.51 |
| trial | 0.67 | serotonin | 0.7 | self control | 0.51 |
| reward anticipation | 0.67 | limbic system | 0.7 | neurobiological | 0.51 |
| pleasure | 0.66 | phenotype | 0.69 | individual difference | 0.51 |
| value | 0.66 | entering | 0.69 | self esteem | 0.51 |
| schedule | 0.66 | aggressive | 0.69 | nucleus accumbens | 0.51 |
| reinforcement learning | 0.65 | ac pc line | 0.69 | self evaluation | 0.5 |
| parent | 0.65 | vulnerability | 0.68 | reinforcement | 0.5 |
| anhedonia | 0.65 | dropout | 0.68 | criticism | 0.5 |
| card | 0.65 | fisher | 0.67 | prospective | 0.5 |
| zero | 0.65 | behavior | 0.67 | zero | 0.5 |
| virtue | 0.65 | negative emotions | 0.67 | incentive | 0.5 |
| dopamine | 0.64 | diagnosis | 0.67 | self concept | 0.5 |
| investigator | 0.64 | positive | 0.67 | anorexia nervosa | 0.5 |
| contingency | 0.64 | cs | 0.67 | impact | 0.5 |
| neurobiological | 0.64 | negative positive | 0.67 | royal | 0.49 |
| decision making | 0.63 | ever | 0.66 | punishment | 0.49 |
| addictive | 0.63 | neutral pictures | 0.66 | worth | 0.49 |
| adaptive | 0.62 | european | 0.66 | eating | 0.49 |
| drink | 0.62 | ventral | 0.66 | investigator | 0.49 |
| probability | 0.62 | amygdala anterior | 0.65 | theory mind | 0.49 |
| reward learning | 0.61 | amygdala hippocampus | 0.65 | impulsivity | 0.48 |
| monetary incentive | 0.61 | nonspecific | 0.65 | losses | 0.48 |
| liking | 0.61 | double | 0.65 | fc | 0.48 |
| substance use disorder | 0.6 | pulvinar | 0.65 | borderline | 0.48 |
| marginal | 0.6 | harm | 0.65 | mental state | 0.48 |
| effort | 0.59 | caudate nucleus | 0.65 | engagement | 0.47 |
| pg | 0.58 | fearful faces | 0.64 | consumption | 0.47 |
| cognitive effort | 0.58 | medicine | 0.64 | ideation | 0.47 |
| sensitive | 0.58 | outcome | 0.64 | self referential | 0.47 |
| token | 0.58 | positive negative | 0.64 | striatum | 0.47 |
| habit | 0.57 | subregion | 0.64 | referential | 0.47 |
| paracingulate gyrus | 0.57 | negative neutral | 0.64 | thought | 0.47 |
| impulsivity | 0.57 | financial | 0.64 | tegmental | 0.47 |
| choose | 0.57 | venous | 0.63 | schedule | 0.47 |
| decision | 0.57 | mixed | 0.63 | social anxiety disorder | 0.47 |
| feedback | 0.57 | pavlovian | 0.63 | alcohol abuse | 0.47 |
| availability | 0.57 | sample | 0.63 | choice | 0.47 |
| sharing | 0.57 | animal | 0.62 | virtue | 0.47 |
| relevant | 0.56 | small | 0.62 | nucleus | 0.46 |
| food | 0.56 | basal forebrain | 0.62 | ventral tegmental area | 0.46 |
| depressive symptom | 0.56 | caudate putamen | 0.62 | prediction error | 0.46 |
| personality disorder | 0.56 | noradrenergic | 0.62 | divergent | 0.46 |
| abuse | 0.56 | basal | 0.62 | monetary reward | 0.46 |
| addictive behavior | 0.55 | reduced | 0.62 | decision making | 0.46 |
| willingness | 0.55 | nucleus accumbens | 0.62 | marginal | 0.46 |
| discounting | 0.55 | blood | 0.62 | substance abuse | 0.46 |
| excitement | 0.55 | excessive | 0.62 | da | 0.46 |
| choice behavior | 0.54 | finding | 0.61 | money | 0.46 |
| substance dependence | 0.54 | accumbens | 0.61 | gambling | 0.45 |
| lesser | 0.54 | psychotic | 0.61 | probability | 0.45 |
| prediction | 0.54 | substantia | 0.61 | mesolimbic | 0.45 |
| gratification | 0.54 | hoc | 0.61 | deliberate | 0.45 |
| prospective | 0.54 | neurobiological | 0.61 | dopamine | 0.45 |
| approach behavior | 0.54 | motivation | 0.61 |  |  |
| driven | 0.54 | threat | 0.61 |  |  |
| eating | 0.54 | history | 0.6 |  |  |
| worth | 0.53 | nigra | 0.6 |  |  |
| cash | 0.53 | substantia nigra | 0.6 |  |  |
| substantia nigra | 0.53 | rsfc | 0.6 |  |  |
| research | 0.53 | superficial | 0.6 |  |  |
| learning | 0.53 | presynaptic | 0.6 |  |  |
| social context | 0.53 | striatum | 0.6 |  |  |
| circadian | 0.53 | parent | 0.59 |  |  |
| a2 | 0.52 | pg | 0.59 |  |  |
| regions orbitofrontal | 0.52 | valence | 0.59 |  |  |
| erotic | 0.52 | neuroticism | 0.59 |  |  |
| addiction | 0.52 | placebo controlled | 0.59 |  |  |
| entering | 0.51 | stria | 0.59 |  |  |
| learn | 0.51 | increased | 0.59 |  |  |
| individual difference | 0.51 | substance | 0.59 |  |  |
| drug addiction | 0.51 | adrenal | 0.59 |  |  |
| avoid | 0.51 | emotional valence | 0.59 |  |  |
| complete | 0.51 | prescription | 0.59 |  |  |
| questionnaire | 0.5 | gain | 0.58 |  |  |
| financial | 0.5 | reward | 0.58 |  |  |
| response | 0.5 | potential | 0.58 |  |  |
| substantia | 0.5 | satiety | 0.58 |  |  |
| insensitivity | 0.5 | la | 0.58 |  |  |
| choice | 0.49 | startle | 0.58 |  |  |
| positive reinforcement | 0.49 | psychiatric diagnosis | 0.58 |  |  |
| pe | 0.49 | trauma | 0.58 |  |  |
| indifference | 0.48 | disorder | 0.58 |  |  |
| medial orbitofrontal cortex | 0.48 | caudate | 0.58 |  |  |
| midbrain | 0.48 | self assessment | 0.58 |  |  |
| negative reinforcement | 0.48 | loss | 0.57 |  |  |
| nigra | 0.48 | basolateral | 0.57 |  |  |
| reward valuation | 0.48 | temperament | 0.57 |  |  |
| desirability | 0.48 | drug | 0.57 |  |  |
| mid | 0.48 | forebrain | 0.57 |  |  |
| ventral pallidum | 0.48 | mediated | 0.56 |  |  |
| mimicry | 0.47 | meta | 0.56 |  |  |
| c5 | 0.47 | effect | 0.56 |  |  |
| c6 | 0.47 | variant | 0.56 |  |  |
| success | 0.46 | skew | 0.56 |  |  |
| making | 0.46 | traumatic | 0.56 |  |  |
| subconscious | 0.46 | dopamine | 0.55 |  |  |
| behavioral inhibition | 0.46 | complete | 0.55 |  |  |
| love | 0.45 | probability | 0.55 |  |  |
| avoidance | 0.45 | approach | 0.55 |  |  |
| operant | 0.45 | total | 0.55 |  |  |
| withdrawal symptoms | 0.45 | dorsal striatum | 0.55 |  |  |
| inhibitory control | 0.45 | collection | 0.55 |  |  |
| engagement | 0.45 | bed nucleus stria terminalis | 0.55 |  |  |
|  |  | sadness | 0.55 |  |  |
|  |  | frustration | 0.55 |  |  |
|  |  | future | 0.55 |  |  |
|  |  | ganglia | 0.54 |  |  |
|  |  | reactive | 0.54 |  |  |
|  |  | basal ganglia | 0.54 |  |  |
|  |  | questionnaire | 0.54 |  |  |
|  |  | williams | 0.54 |  |  |
|  |  | consumption | 0.54 |  |  |
|  |  | escape | 0.54 |  |  |
|  |  | striatal | 0.53 |  |  |
|  |  | reported | 0.53 |  |  |
|  |  | monetary | 0.53 |  |  |
|  |  | explicit | 0.53 |  |  |
|  |  | operant | 0.53 |  |  |
|  |  | pallidus | 0.53 |  |  |
|  |  | substance abuse | 0.53 |  |  |
|  |  | basolateral amygdala | 0.53 |  |  |
|  |  | seeking | 0.53 |  |  |
|  |  | hypervigilance | 0.53 |  |  |
|  |  | approach behavior | 0.52 |  |  |
|  |  | learning | 0.52 |  |  |
|  |  | reinforcement | 0.52 |  |  |
|  |  | immediate | 0.52 |  |  |
|  |  | abuse | 0.52 |  |  |
|  |  | personality disorder | 0.52 |  |  |
|  |  | emotion regulation | 0.52 |  |  |
|  |  | rewarding | 0.51 |  |  |
|  |  | unpleasant | 0.51 |  |  |
|  |  | life | 0.51 |  |  |
|  |  | pavlovian conditioning | 0.51 |  |  |
|  |  | transmission | 0.51 |  |  |
|  |  | reward processing | 0.51 |  |  |
|  |  | cortex amygdala | 0.51 |  |  |
|  |  | vigilance | 0.51 |  |  |
|  |  | emotional faces | 0.51 |  |  |
|  |  | symptom severity | 0.51 |  |  |
|  |  | psychosocial | 0.51 |  |  |
|  |  | social context | 0.51 |  |  |
|  |  | behavioral inhibition | 0.51 |  |  |
|  |  | ventral striatum | 0.51 |  |  |
|  |  | amygdaloid | 0.51 |  |  |
|  |  | enlargement | 0.51 |  |  |
|  |  | lesser | 0.5 |  |  |
|  |  | success | 0.5 |  |  |
|  |  | pad | 0.5 |  |  |
|  |  | breath | 0.5 |  |  |
|  |  | sleep | 0.5 |  |  |
|  |  | bias | 0.5 |  |  |
|  |  | amygdala insula | 0.5 |  |  |
|  |  | pleasure | 0.5 |  |  |
|  |  | globus pallidus | 0.5 |  |  |
|  |  | region 5 | 0.5 |  |  |
|  |  | depression | 0.5 |  |  |
|  |  | habit | 0.5 |  |  |
|  |  | regulation | 0.5 |  |  |
|  |  | manic | 0.5 |  |  |
|  |  | free | 0.5 |  |  |
|  |  | incentive | 0.49 |  |  |
|  |  | desired | 0.49 |  |  |
|  |  | novelty | 0.49 |  |  |
|  |  | mania | 0.49 |  |  |
|  |  | populations | 0.49 |  |  |
|  |  | dysregulation | 0.49 |  |  |
|  |  | availability | 0.49 |  |  |
|  |  | subcortical | 0.49 |  |  |
|  |  | unexpected | 0.49 |  |  |
|  |  | losses | 0.49 |  |  |
|  |  | extended amygdala | 0.49 |  |  |
|  |  | money | 0.49 |  |  |
|  |  | personality traits | 0.49 |  |  |
|  |  | value | 0.49 |  |  |
|  |  | post | 0.48 |  |  |
|  |  | critical | 0.48 |  |  |
|  |  | punishment | 0.48 |  |  |
|  |  | avoidance learning | 0.48 |  |  |
|  |  | limitation | 0.48 |  |  |
|  |  | sexual | 0.48 |  |  |
|  |  | life stress | 0.48 |  |  |
|  |  | loss aversion | 0.48 |  |  |
|  |  | mammalian | 0.48 |  |  |
|  |  | wake | 0.47 |  |  |
|  |  | explicit knowledge | 0.47 |  |  |
|  |  | reinforcement schedule | 0.47 |  |  |
|  |  | tegmental | 0.47 |  |  |
|  |  | affective disorder | 0.47 |  |  |
|  |  | central nucleus amygdala | 0.47 |  |  |
|  |  | gambling | 0.47 |  |  |
|  |  | may | 0.47 |  |  |
|  |  | contingency | 0.47 |  |  |
|  |  | prediction error | 0.47 |  |  |
|  |  | feeding | 0.47 |  |  |
|  |  | ventral tegmental area | 0.47 |  |  |
|  |  | schedule | 0.47 |  |  |
|  |  | trait anxiety | 0.47 |  |  |
|  |  | neutral faces | 0.46 |  |  |
|  |  | shin | 0.46 |  |  |
|  |  | drink | 0.46 |  |  |
|  |  | da | 0.46 |  |  |
|  |  | aversion | 0.46 |  |  |
|  |  | substance dependence | 0.46 |  |  |
|  |  | amygdala cortical | 0.46 |  |  |
|  |  | hyperactivity | 0.46 |  |  |
|  |  | linking | 0.46 |  |  |
|  |  | sharing | 0.46 |  |  |
|  |  | alcohol abuse | 0.46 |  |  |
|  |  | inconsistent | 0.46 |  |  |
|  |  | functional connectivity | 0.46 |  |  |
|  |  | volition | 0.46 |  |  |
|  |  | mesolimbic | 0.46 |  |  |
|  |  | neurofeedback | 0.46 |  |  |
|  |  | monetary reward | 0.46 |  |  |
|  |  | anhedonia | 0.45 |  |  |
|  |  | worry | 0.45 |  |  |
|  |  | female | 0.45 |  |  |
|  |  | thalamic | 0.45 |  |  |
|  |  | startle reflex | 0.45 |  |  |
|  |  | midbrain | 0.45 |  |  |
|  |  | social behavior | 0.45 |  |  |
|  |  | marker | 0.45 |  |  |
|  |  | card | 0.45 |  |  |
|  |  | depressive symptom | 0.45 |  |  |
|  |  | addiction | 0.45 |  |  |

The terms are sorted by the order of similarity score. The terms with a similarity score > 0.45 were listed.

**Table S10. Decoded results in migration stage phase (Including anatomical and uninterpretable terms)**

| **Brand love**  **and**  **Interpersonal romantic love** | | **Brand love**  **>**  **Interpersonal romantic love** | | **Brand love**  **<**  **Interpersonal romantic love** | |
| --- | --- | --- | --- | --- | --- |
| Term | Similarity | Term | Similarity | Term | Similarity |
| dorsolateral | 1 | vmpfc | 1 | anterior insula | 1 |
| dorsolateral prefrontal cortex | 0.97 | medial prefrontal cortex | 0.96 | modified | 0.9 |
| prefrontal | 0.9 | ventromedial | 0.88 | insula | 0.84 |
| regulation | 0.86 | network | 0.88 | supporting | 0.79 |
| cognitive control | 0.85 | default | 0.86 | frontal operculum | 0.78 |
| prefrontal cortex | 0.84 | ventromedial prefrontal cortex | 0.83 | dorsal | 0.78 |
| pfc | 0.83 | self referential | 0.82 | death | 0.75 |
| dlpfc | 0.82 | trait | 0.8 | flanker | 0.74 |
| reappraisal | 0.76 | referential | 0.8 | reaction | 0.74 |
| dorsolateral pfc | 0.76 | mpfc | 0.77 | awareness | 0.73 |
| dorsolateral prefrontal | 0.75 | posterior cingulate cortex | 0.77 | feeling | 0.73 |
| ventrolateral | 0.74 | thought | 0.75 | leaving | 0.72 |
| vlpfc | 0.74 | conscious | 0.74 | demand | 0.71 |
| norm | 0.73 | pcc | 0.74 | resource | 0.71 |
| dorsomedial | 0.72 | network dmn | 0.73 | conflict | 0.71 |
| lateral prefrontal cortex | 0.72 | midline | 0.72 | activation | 0.71 |
| economic | 0.71 | dmn | 0.71 | experience | 0.7 |
| ventrolateral prefrontal cortex | 0.69 | default mode | 0.7 | involved | 0.7 |
| anterior insula | 0.68 | failure | 0.68 | difficult | 0.7 |
| emotion regulation | 0.66 | ventral medial prefrontal cortex | 0.68 | accuracy | 0.69 |
| dorsomedial prefrontal | 0.64 | default network | 0.66 | selection | 0.69 |
| social norm | 0.63 | ventromedial prefrontal | 0.66 | rejection | 0.69 |
| wisconsin | 0.62 | induction | 0.66 | reaction time | 0.69 |
| frontal | 0.62 | precuneus posterior | 0.66 | distractor | 0.68 |
| inappropriate | 0.62 | social cognitive | 0.65 | instruction | 0.68 |
| prefrontal cortical | 0.62 | rest | 0.64 | meta | 0.68 |
| psychological | 0.62 | medial pfc | 0.64 | interoceptive | 0.67 |
| broad | 0.62 | gift | 0.64 | autonomic | 0.67 |
| setting | 0.61 | pc | 0.63 | norm | 0.67 |
| impulse | 0.61 | borne | 0.63 | prone | 0.67 |
| medial prefrontal | 0.61 | anti | 0.63 | taking | 0.66 |
| frontal cortex | 0.6 | focused | 0.62 | decision | 0.66 |
| modified | 0.6 | posterior cingulate | 0.62 | salience | 0.65 |
| sorting | 0.59 | wandering | 0.61 | bodily | 0.65 |
| strategy | 0.59 | mother | 0.61 | learn | 0.65 |
| ba46 | 0.59 | rumination | 0.61 | bed | 0.65 |
| regulate | 0.59 | mood | 0.61 | insula inferior | 0.65 |
| conflict | 0.59 | choice | 0.61 | mediating | 0.65 |
| cortex dmpfc | 0.58 | mind | 0.6 | cortex posterior | 0.64 |
| anxiety | 0.58 | psychotic disorder | 0.6 | children | 0.64 |
| postsynaptic | 0.57 | youth | 0.59 | partner | 0.64 |
| executive | 0.57 | emotional responses | 0.58 | empathy | 0.64 |
| rule | 0.56 | task positive | 0.58 | pervasive | 0.64 |
| anterior prefrontal | 0.55 | negative | 0.58 | interference | 0.63 |
| game | 0.55 | inadequate | 0.57 | insular cortex | 0.63 |
| negative emotion | 0.54 | evaluation | 0.56 | posterior insula | 0.63 |
| trait anxiety | 0.54 | psychotic | 0.56 | response inhibition | 0.62 |
| dorsolateral prefrontal cortices | 0.54 | positive | 0.56 | transition | 0.62 |
| dmpfc | 0.53 | impact | 0.55 | common | 0.62 |
| miller | 0.53 | positive negative | 0.55 | task | 0.62 |
| major depression | 0.53 | public | 0.55 | triangular | 0.62 |
| emotional responses | 0.53 | infancy | 0.55 | back | 0.62 |
| inferior frontal | 0.52 | dorsal medial | 0.55 | executive | 0.62 |
| inferior prefrontal | 0.52 | medial prefrontal | 0.55 | nature | 0.61 |
| behavior | 0.52 | fisher | 0.55 | hard | 0.61 |
| brodmann area 9 | 0.52 | anterior medial | 0.54 | acceptance | 0.61 |
| lateral medial | 0.52 | introspection | 0.53 | inferior | 0.61 |
| response execution | 0.52 | dmpfc | 0.53 | broad | 0.61 |
| lateral frontal | 0.51 | cingulate | 0.52 | generation | 0.61 |
| lateral prefrontal | 0.51 | cognitive load | 0.52 | anterior | 0.61 |
| handling | 0.51 | oriented | 0.52 | cortex anterior | 0.6 |
| insula | 0.5 | jones | 0.51 | psychological | 0.6 |
| ventromedial | 0.5 | component | 0.51 | working | 0.59 |
| punish | 0.5 | orbitofrontal | 0.51 | mfg | 0.59 |
| orbital | 0.49 | drug | 0.5 | ex | 0.59 |
| ifg | 0.49 | dorsal attention | 0.5 | operculum | 0.59 |
| prefrontal cortices | 0.49 | mental processes | 0.5 | understanding | 0.59 |
| frontal pole | 0.49 | cortex pcc | 0.49 | response selection | 0.59 |
| choice | 0.49 | goal directed | 0.49 | executive control | 0.58 |
| reactive | 0.48 | resting | 0.49 | rule | 0.58 |
| cyst | 0.48 | cancer | 0.49 | mid | 0.58 |
| adrenergic | 0.48 | awakening | 0.49 | cognitive control | 0.58 |
| frontal lobe | 0.48 | subclinical | 0.48 | lack | 0.58 |
| war | 0.48 | ventral anterior cingulate | 0.48 | response latency | 0.58 |
| cognitive | 0.48 | anterior cingulate cortex | 0.48 | inferior frontal | 0.58 |
| control processes | 0.47 | paracingulate | 0.48 | pre | 0.57 |
| bipolar | 0.47 | self knowledge | 0.47 | classical | 0.57 |
| salivary | 0.47 | subgenual | 0.47 | maintenance | 0.57 |
| bipolar disorder | 0.47 | loss consciousness | 0.47 | frontal | 0.57 |
| acceptance | 0.47 | member | 0.47 | frontal gyrus | 0.57 |
| primitive | 0.47 | attachment | 0.47 | disgust | 0.57 |
| triangular | 0.46 | ofc | 0.47 | insula anterior | 0.57 |
| working | 0.46 | external | 0.47 | regulation | 0.56 |
| prefrontal region | 0.46 | social desirability | 0.46 | relay | 0.56 |
| leaving | 0.46 | appraisal | 0.46 | stroop | 0.56 |
| maintenance | 0.46 | cingulate cortex | 0.46 | sma | 0.56 |
| sibling | 0.46 | anterior cingulate | 0.46 | set | 0.56 |
| consensus | 0.45 | kennedy | 0.46 | middle frontal gyrus | 0.56 |
| ventromedial prefrontal cortex | 0.45 | prefrontal | 0.46 | efficiency | 0.56 |
| executive function | 0.45 | price | 0.46 | cortex insula | 0.55 |
| double | 0.45 | sedentary | 0.45 | behavioral performance | 0.55 |
| resource | 0.45 | regions posterior cingulate | 0.45 | opercularis | 0.55 |
| rostrolateral | 0.45 | thinking | 0.45 | ventral anterior | 0.55 |
| opiate | 0.45 | currently | 0.45 | splitting | 0.55 |
| cortex | 0.45 | abuse | 0.45 | adjustment | 0.55 |
| frontopolar cortex | 0.45 |  |  | ifg | 0.54 |
| inhibitory control | 0.45 |  |  | sets | 0.54 |
| medial pfc | 0.45 |  |  | line | 0.54 |
| flanker | 0.45 |  |  | executive function | 0.54 |
| working memory | 0.45 |  |  | stroop task | 0.54 |
| brodmann area 10 | 0.45 |  |  | sense | 0.54 |
|  |  |  |  | response time | 0.54 |
|  |  |  |  | load | 0.53 |
|  |  |  |  | salience network | 0.53 |
|  |  |  |  | middle frontal | 0.53 |
|  |  |  |  | psychophysiological | 0.53 |
|  |  |  |  | sympathetic | 0.53 |
|  |  |  |  | breathing | 0.53 |
|  |  |  |  | uncertainty | 0.53 |
|  |  |  |  | ba | 0.53 |
|  |  |  |  | frontal cortex | 0.53 |
|  |  |  |  | working memory | 0.53 |
|  |  |  |  | strategies | 0.53 |
|  |  |  |  | insular region | 0.53 |
|  |  |  |  | mental effort | 0.53 |
|  |  |  |  | cortex bilaterally | 0.53 |
|  |  |  |  | subdivision | 0.52 |
|  |  |  |  | interpersonal | 0.52 |
|  |  |  |  | setting | 0.51 |
|  |  |  |  | respiratory | 0.51 |
|  |  |  |  | dorsal anterior | 0.51 |
|  |  |  |  | management | 0.51 |
|  |  |  |  | cardiac | 0.51 |
|  |  |  |  | sub | 0.51 |
|  |  |  |  | chosen | 0.51 |
|  |  |  |  | inhibitory control | 0.51 |
|  |  |  |  | insular cortices | 0.51 |
|  |  |  |  | computer | 0.51 |
|  |  |  |  | frontal gyri | 0.5 |
|  |  |  |  | behavioral measures | 0.5 |
|  |  |  |  | broca area | 0.5 |
|  |  |  |  | incongruent | 0.5 |
|  |  |  |  | middle | 0.5 |
|  |  |  |  | inferior frontal gyrus | 0.5 |
|  |  |  |  | attention | 0.5 |
|  |  |  |  | making | 0.5 |
|  |  |  |  | empathic | 0.5 |
|  |  |  |  | medial frontal | 0.5 |
|  |  |  |  | parasympathetic | 0.5 |
|  |  |  |  | cortex dorsal | 0.49 |
|  |  |  |  | cue | 0.49 |
|  |  |  |  | problem | 0.49 |
|  |  |  |  | gustatory | 0.49 |
|  |  |  |  | real | 0.49 |
|  |  |  |  | poor | 0.49 |
|  |  |  |  | cingulate cortices | 0.49 |
|  |  |  |  | dual | 0.49 |
|  |  |  |  | game | 0.49 |
|  |  |  |  | skin conductance | 0.49 |
|  |  |  |  | claustrum | 0.49 |
|  |  |  |  | cortex inferior | 0.49 |
|  |  |  |  | manipulation | 0.48 |
|  |  |  |  | self regulation | 0.48 |
|  |  |  |  | intense | 0.48 |
|  |  |  |  | proactive | 0.48 |
|  |  |  |  | task difficulty | 0.48 |
|  |  |  |  | ascending | 0.48 |
|  |  |  |  | capacity | 0.48 |
|  |  |  |  | style | 0.48 |
|  |  |  |  | inferior middle | 0.47 |
|  |  |  |  | sector | 0.47 |
|  |  |  |  | choice | 0.47 |
|  |  |  |  | pre supplementary | 0.47 |
|  |  |  |  | human development | 0.47 |
|  |  |  |  | stop | 0.47 |
|  |  |  |  | starts | 0.47 |
|  |  |  |  | otherwise | 0.47 |
|  |  |  |  | reversed | 0.47 |
|  |  |  |  | rating | 0.46 |
|  |  |  |  | button | 0.46 |
|  |  |  |  | visceral | 0.46 |
|  |  |  |  | acc | 0.46 |
|  |  |  |  | critchley | 0.46 |
|  |  |  |  | third | 0.46 |
|  |  |  |  | altruism | 0.46 |
|  |  |  |  | phobia | 0.46 |
|  |  |  |  | medial superior | 0.46 |
|  |  |  |  | opercular cortex | 0.46 |
|  |  |  |  | regulate | 0.46 |
|  |  |  |  | decision making | 0.46 |
|  |  |  |  | negativity | 0.46 |
|  |  |  |  | pre sma | 0.45 |
|  |  |  |  | embarrassment | 0.45 |
|  |  |  |  | safe | 0.45 |
|  |  |  |  | letter | 0.45 |
|  |  |  |  | pro | 0.45 |
|  |  |  |  | task relevant | 0.45 |
|  |  |  |  | frontal cortices | 0.45 |
|  |  |  |  | cytoarchitecture | 0.45 |
|  |  |  |  | excess | 0.45 |
|  |  |  |  | neural | 0.45 |

The terms are sorted by the order of similarity score. The terms with a similarity score > 0.45 were listed.

**Table S11. Decoded results in stable stage phase (Including anatomical and uninterpretable terms)**

| **Brand love**  **and**  **Interpersonal romantic love** | | **Brand love**  **>**  **Interpersonal romantic love** | | **Brand love**  **<**  **Interpersonal romantic love** | |
| --- | --- | --- | --- | --- | --- |
| Term | Similarity | Term | Similarity | Term | Similarity |
| putamen | 1 | putamen | 1 | midbrain | 1 |
| basal | 0.99 | basal | 0.85 | nucleus | 0.83 |
| ganglia | 0.98 | ganglia | 0.84 | outcome | 0.75 |
| basal ganglia | 0.98 | basal ganglia | 0.84 | reward | 0.73 |
| meta | 0.91 | pre | 0.79 | marginal | 0.73 |
| modified | 0.89 | left putamen | 0.77 | ventral striatum | 0.72 |
| right putamen | 0.87 | pallidus | 0.76 | gain | 0.72 |
| pallidus | 0.86 | globus | 0.72 | motivation | 0.72 |
| globus pallidus | 0.82 | right putamen | 0.72 | collection | 0.71 |
| behavior | 0.81 | globus pallidus | 0.71 | substance | 0.71 |
| caudate putamen | 0.79 | meta | 0.69 | transmission | 0.7 |
| left putamen | 0.79 | supplementary | 0.69 | ventral | 0.69 |
| flexibility | 0.77 | subcortical | 0.69 | accumbens | 0.68 |
| striatal | 0.77 | sma | 0.67 | thalamus | 0.67 |
| making | 0.77 | modified | 0.65 | nucleus accumbens | 0.67 |
| learn | 0.76 | making | 0.64 | monetary | 0.67 |
| striatum | 0.76 | influence | 0.64 | striatum | 0.67 |
| globus | 0.74 | flexibility | 0.62 | loss | 0.66 |
| insula | 0.74 | supplementary motor area | 0.61 | positive | 0.66 |
| anterior insula | 0.74 | pre sma | 0.61 | blood | 0.66 |
| nigra | 0.73 | insula | 0.61 | consumption | 0.65 |
| dopamine | 0.73 | related | 0.61 | dopamine | 0.65 |
| experience | 0.72 | parkinson disease | 0.6 | self report | 0.65 |
| substantia | 0.71 | behavior | 0.6 | questionnaire | 0.64 |
| dorsal striatum | 0.7 | oral | 0.6 | seeking | 0.64 |
| salience | 0.7 | precentral | 0.6 | neurobiological | 0.64 |
| neurobiological | 0.7 | likelihood | 0.6 | reward processing | 0.64 |
| caudate nucleus | 0.7 | pre supplementary | 0.6 | anticipation | 0.64 |
| nucleus | 0.69 | proper | 0.59 | unexpected | 0.63 |
| ventral | 0.69 | system | 0.59 | reinforcement | 0.63 |
| availability | 0.68 | supporting | 0.58 | rewarding | 0.63 |
| gain | 0.68 | parkinson | 0.57 | sharing | 0.63 |
| motivation | 0.67 | daily life | 0.56 | receptor | 0.63 |
| habit | 0.67 | striatal | 0.56 | signaling | 0.63 |
| substantia nigra | 0.67 | experience | 0.55 | expected | 0.62 |
| feeling | 0.67 | delusions | 0.55 | withdrawal | 0.62 |
| uncertainty | 0.66 | caudate putamen | 0.55 | incentive | 0.62 |
| lentiform | 0.66 | stop | 0.55 | substantia | 0.61 |
| outcome | 0.66 | inhibition | 0.55 | substantia nigra | 0.61 |
| lentiform nucleus | 0.66 | participate | 0.54 | immediate | 0.61 |
| financial | 0.66 | feeling | 0.54 | nigra | 0.61 |
| likelihood | 0.66 | transition | 0.54 | sleep | 0.61 |
| desired | 0.65 | stopping | 0.54 | money | 0.6 |
| supporting | 0.64 | learn | 0.54 | punishment | 0.6 |
| decision | 0.64 | premotor | 0.53 | losses | 0.6 |
| partner | 0.64 | overactivity | 0.53 | probability | 0.59 |
| food | 0.64 | motor | 0.53 | zero | 0.59 |
| love | 0.64 | nigra | 0.53 | parent | 0.59 |
| mid | 0.64 | sequence | 0.53 | tegmental | 0.59 |
| pre | 0.63 | salience | 0.52 | trial | 0.59 |
| effort | 0.63 | magnetic | 0.51 | value | 0.59 |
| collection | 0.63 | frontal operculum | 0.51 | desired | 0.58 |
| thalamic nuclei | 0.63 | pd | 0.51 | dorsal striatum | 0.58 |
| related | 0.63 | anterior insula | 0.51 | ventral tegmental area | 0.58 |
| consumption | 0.62 | paradigm | 0.51 | availability | 0.58 |
| taste | 0.62 | objective | 0.5 | monetary reward | 0.58 |
| trial | 0.62 | grand | 0.5 | prediction error | 0.58 |
| substance | 0.62 | dysfunction | 0.5 | alcohol abuse | 0.57 |
| blood | 0.62 | respiratory | 0.5 | da | 0.57 |
| accumbens | 0.62 | substantia | 0.5 | personality traits | 0.57 |
| money | 0.62 | subject | 0.5 | mesolimbic | 0.56 |
| transmission | 0.62 | overt | 0.49 | positive negative | 0.55 |
| oral | 0.62 | physiological | 0.49 | drink | 0.55 |
| physiological | 0.62 | program | 0.49 | reward anticipation | 0.55 |
| reward | 0.62 | motor response | 0.49 | serotonin | 0.55 |
| subcortical | 0.61 | fatigue | 0.49 | schedule | 0.55 |
| caudate | 0.61 | inhibitory control | 0.48 | pleasure | 0.54 |
| nucleus accumbens | 0.61 | floor | 0.48 | anhedonia | 0.54 |
| cognitive effort | 0.61 | button | 0.48 | investigator | 0.54 |
| leaving | 0.61 | classical | 0.48 | history | 0.54 |
| learning | 0.6 | leaving | 0.48 | substance abuse | 0.54 |
| subcortical structures | 0.6 | precentral gyrus | 0.48 | caudate putamen | 0.54 |
| rating | 0.6 | dopamine | 0.48 | reinforcement learning | 0.54 |
| decision making | 0.6 | pallidal | 0.48 | behavior | 0.53 |
| autonomic | 0.6 | partner | 0.48 | reception | 0.53 |
| linked | 0.6 | generation | 0.48 | sensitive | 0.53 |
| transition | 0.59 | interoceptive | 0.48 | expectancy | 0.53 |
| bed | 0.59 | external | 0.48 | gambling | 0.53 |
| objective | 0.59 | motor area | 0.47 | prediction | 0.53 |
| inhibitory control | 0.59 | bed | 0.47 | addictive | 0.52 |
| interoceptive | 0.59 | lentiform | 0.47 | virtue | 0.52 |
| substance dependence | 0.59 | effort | 0.47 | arterial | 0.52 |
| expected | 0.58 | subcortical nuclei | 0.47 | striatal | 0.52 |
| anticipation | 0.58 | pervasive | 0.47 | driven | 0.51 |
| subject | 0.58 | striatum | 0.47 | minimization | 0.51 |
| rejection | 0.58 | subthalamic | 0.47 | social context | 0.51 |
| social context | 0.58 | response selection | 0.47 | raphe | 0.51 |
| pervasive | 0.58 | review | 0.47 | basal | 0.5 |
| unexpected | 0.58 | prone | 0.46 | subcortical structures | 0.5 |
| fatigue | 0.58 | posterior insula | 0.46 | card | 0.5 |
| treatment | 0.58 | habit | 0.46 | monetary incentive | 0.5 |
| reinforcement | 0.58 | extreme | 0.46 | pallidus | 0.5 |
| respiratory | 0.57 | simple | 0.46 | financial | 0.5 |
| extreme | 0.57 | red nucleus | 0.46 | spin | 0.49 |
| seeking | 0.57 | lentiform nucleus | 0.46 | liking | 0.49 |
| intense | 0.57 | bodily | 0.46 | reward learning | 0.49 |
| pleasure | 0.57 | excessive | 0.46 | research | 0.49 |
| monetary | 0.57 | insular cortex | 0.46 | circadian | 0.49 |
| prone | 0.57 | sma proper | 0.46 | persistent | 0.49 |
| delusions | 0.57 | huntington | 0.46 | depressive symptom | 0.48 |
| posterior insula | 0.56 | awareness | 0.45 | learning | 0.48 |
| loss | 0.56 | stop signal | 0.45 | caudate nucleus | 0.48 |
| driven | 0.56 | mediating | 0.45 | addiction | 0.48 |
| subcortical nuclei | 0.56 | premotor cortex | 0.45 | habit | 0.48 |
| positive | 0.56 | instruction | 0.45 | rating | 0.48 |
| hunger | 0.56 | autonomic | 0.45 | complete | 0.47 |
| thalamus | 0.56 | dorsal striatum | 0.45 | substance dependence | 0.47 |
| influence | 0.56 | psychomotor | 0.45 | drug addiction | 0.47 |
| bodily | 0.56 | trigger | 0.45 | substance use disorder | 0.47 |
| mediating | 0.56 |  |  | adaptive | 0.47 |
| pharmacological | 0.55 |  |  | arterial spin | 0.47 |
| altered | 0.55 |  |  | syndrome | 0.47 |
| awareness | 0.55 |  |  | contingency | 0.47 |
| sma | 0.55 |  |  | connectivity | 0.46 |
| gustatory | 0.55 |  |  | excitement | 0.46 |
| intrinsic | 0.55 |  |  | approach | 0.46 |
| frontal operculum | 0.54 |  |  | pg | 0.46 |
| insular cortex | 0.54 |  |  | choice behavior | 0.46 |
| losses | 0.54 |  |  | brainstem | 0.46 |
| psychophysiological | 0.54 |  |  | fatigue | 0.46 |
| reward processing | 0.54 |  |  | psychosocial | 0.45 |
| da | 0.54 |  |  | effort | 0.45 |
| classical | 0.54 |  |  | thalamic | 0.45 |
| sharing | 0.54 |  |  | meta | 0.45 |
| rewarding | 0.54 |  |  | erotic | 0.45 |
| urine | 0.54 |  |  | response | 0.45 |
| adjustment | 0.53 |  |  | monoamine | 0.45 |
| complete | 0.53 |  |  | addictive behavior | 0.45 |
| cardiac | 0.53 |  |  | globus pallidus | 0.45 |
| impulsivity | 0.53 |  |  | token | 0.45 |
| signaling | 0.53 |  |  |  |  |
| incentive | 0.53 |  |  |  |  |
| probability | 0.53 |  |  |  |  |
| insensitivity | 0.53 |  |  |  |  |
| red nucleus | 0.53 |  |  |  |  |
| questionnaire | 0.53 |  |  |  |  |
| heart | 0.53 |  |  |  |  |
| response | 0.53 |  |  |  |  |
| stopping | 0.52 |  |  |  |  |
| diminished | 0.52 |  |  |  |  |
| tegmental | 0.52 |  |  |  |  |
| head caudate nucleus | 0.52 |  |  |  |  |
| receptor | 0.52 |  |  |  |  |
| drink | 0.52 |  |  |  |  |
| feedback | 0.52 |  |  |  |  |
| shock | 0.52 |  |  |  |  |
| ventral tegmental area | 0.51 |  |  |  |  |
| card | 0.51 |  |  |  |  |
| subthalamic | 0.51 |  |  |  |  |
| relay | 0.51 |  |  |  |  |
| punishment | 0.51 |  |  |  |  |
| insula anterior | 0.51 |  |  |  |  |
| liking | 0.51 |  |  |  |  |
| avoidance behavior | 0.51 |  |  |  |  |
| disgust | 0.51 |  |  |  |  |
| prediction error | 0.51 |  |  |  |  |
| skin conductance | 0.51 |  |  |  |  |
| zero | 0.51 |  |  |  |  |
| marginal | 0.5 |  |  |  |  |
| individual difference | 0.5 |  |  |  |  |
| pre supplementary | 0.5 |  |  |  |  |
| stop | 0.5 |  |  |  |  |
| insula inferior | 0.5 |  |  |  |  |
| dysfunction | 0.5 |  |  |  |  |
| anhedonia | 0.5 |  |  |  |  |
| eating | 0.5 |  |  |  |  |
| ventral anterior | 0.5 |  |  |  |  |
| pallidal | 0.5 |  |  |  |  |
| pencil | 0.5 |  |  |  |  |
| breathing | 0.5 |  |  |  |  |
| reinforcement learning | 0.5 |  |  |  |  |
| alcohol abuse | 0.5 |  |  |  |  |
| acceptance | 0.5 |  |  |  |  |
| consumer | 0.5 |  |  |  |  |
| excess | 0.5 |  |  |  |  |
| fronto striatal | 0.49 |  |  |  |  |
| withdrawal | 0.49 |  |  |  |  |
| subthalamic nucleus | 0.49 |  |  |  |  |
| self report | 0.49 |  |  |  |  |
| claustrum | 0.49 |  |  |  |  |
| severity | 0.49 |  |  |  |  |
| sweet | 0.49 |  |  |  |  |
| mediodorsal | 0.49 |  |  |  |  |
| mesolimbic | 0.49 |  |  |  |  |
| safe | 0.49 |  |  |  |  |
| reception | 0.49 |  |  |  |  |
| monetary reward | 0.49 |  |  |  |  |
| monoamine | 0.48 |  |  |  |  |
| intralaminar | 0.48 |  |  |  |  |
| pre sma | 0.48 |  |  |  |  |
| immediate | 0.48 |  |  |  |  |
| ideation | 0.48 |  |  |  |  |
| inflammatory | 0.48 |  |  |  |  |
| expectation | 0.48 |  |  |  |  |
| medication | 0.48 |  |  |  |  |
| salience network | 0.48 |  |  |  |  |
| nigrostriatal | 0.48 |  |  |  |  |
| weight | 0.48 |  |  |  |  |
| sympathetic | 0.48 |  |  |  |  |
| discounting | 0.47 |  |  |  |  |
| nuclei basal | 0.47 |  |  |  |  |
| daily life | 0.47 |  |  |  |  |
| addiction | 0.47 |  |  |  |  |
| insular cortices | 0.47 |  |  |  |  |
| proper | 0.47 |  |  |  |  |
| insular region | 0.47 |  |  |  |  |
| visceral | 0.47 |  |  |  |  |
| abstinence | 0.47 |  |  |  |  |
| addictive | 0.47 |  |  |  |  |
| habit learning | 0.47 |  |  |  |  |
| erotic | 0.47 |  |  |  |  |
| gambling | 0.47 |  |  |  |  |
| empathy | 0.47 |  |  |  |  |
| cortex anterior | 0.46 |  |  |  |  |
| neuropsychiatric | 0.46 |  |  |  |  |
| suicidal ideation | 0.46 |  |  |  |  |
| prediction | 0.46 |  |  |  |  |
| regulation | 0.46 |  |  |  |  |
| reward anticipation | 0.46 |  |  |  |  |
| risk aversion | 0.46 |  |  |  |  |
| overactivity | 0.46 |  |  |  |  |
| heart rate | 0.46 |  |  |  |  |
| smoking | 0.46 |  |  |  |  |
| expectancy | 0.46 |  |  |  |  |
| positive negative | 0.46 |  |  |  |  |
| boredom | 0.46 |  |  |  |  |
| island | 0.46 |  |  |  |  |
| consummatory | 0.46 |  |  |  |  |
| cingulate cortices | 0.46 |  |  |  |  |
| salt | 0.46 |  |  |  |  |
| choice | 0.46 |  |  |  |  |
| somatic | 0.45 |  |  |  |  |
| gratification | 0.45 |  |  |  |  |
| magnetic | 0.45 |  |  |  |  |
| lateral globus pallidus | 0.45 |  |  |  |  |
| midbrain | 0.45 |  |  |  |  |
| virtue | 0.45 |  |  |  |  |
| substance abuse | 0.45 |  |  |  |  |
| subdivision | 0.45 |  |  |  |  |
| choose | 0.45 |  |  |  |  |
| minimization | 0.45 |  |  |  |  |
| proneness | 0.45 |  |  |  |  |
| personality traits | 0.45 |  |  |  |  |
| smokers | 0.45 |  |  |  |  |
| rate | 0.45 |  |  |  |  |
| phobia | 0.45 |  |  |  |  |

The terms are sorted by the order of similarity score. The terms with a similarity score > 0.45 were listed.

**Table S12. List of contributing publications related to “flexibility”**

| **Title** | **Year** | **Auther** |
| --- | --- | --- |
| Neural predictors of moment-to-moment fluctuations in cognitive flexibility | 2008 | Leber et al. |
| Striatal activation as a neural link between cognitive and perceptual flexibility | 2016 | Sekutowicz et al. |
| Stochastic Dynamics Underlying Cognitive Stability and Flexibility | 2015 | Ueltzhöffer et al. |
| A developmental study on the neural circuitry mediating response flexibility in bipolar disorder | 2013 | Weathers et al. |
| Dissociable fronto-striatal effects of dopamine D2 receptor stimulation on cognitive vs. motor flexibility | 2013 | Stelzel et al. |
| Cognitive flexibility in adolescence: Neural and behavioral mechanisms of reward prediction error processing in adaptive decision making during development | 2015 | Hauser et al. |
| Neural Correlates of Attentional Flexibility during Approach and Avoidance Motivation | 2015 | Calcott and Berkman |
| An fMRI investigation of the relationship between future imagination and cognitive flexibility | 2017 | Roberts et al. |
| Neural correlates of cognitive flexibility in children at risk for bipolar disorder | 2012 | Kim et al. |
| Cognitive flexibility in internet addicts: fMRI evidence from difficult-to-easy and easy-to-difficult switching situations | 2014 | Dong et al. |
| Neural Basis of Impaired Cognitive Flexibility in Patients with Anorexia Nervosa | 2013 | Sato et al. |
| Embodied cognitive flexibility and neuroplasticity following Quadrato Motor Training | 2015 | Ben-Soussan et al. |
| Aberrant Function of Learning and Cognitive Control Networks Underlie Inefficient Cognitive Flexibility in Anorexia Nervosa: A Cross-Sectional fMRI Study | 2015 | Lao-Kaim et al. |
| Association of creative achievement with cognitive flexibility by a combined voxel-based morphometry and resting-state functional connectivity study | 2014 | Chen et al. |
| The bilingual brain: Flexibility and control in the human cortex | 2013 | Buchweitz and Prat |
| Cognitive flexibility depends on white matter microstructure of the basal ganglia | 2014 | Van Schouwenburg et al. |
| Dimensional Change Card Sort performance associated with age-related differences in functional connectivity of lateral prefrontal cortex | 2013 | Ezekiel et al. |
| Affective Modulation of Cognitive Control is Determined by Performance-Contingency and Mediated by Ventromedial Prefrontal and Cingulate Cortex | 2013 | Braem et al. |
| Switch the itch: A naturalistic follow-up study on the neural correlates of cognitive flexibility in obsessive-compulsive disorder | 2013 | Vriend et al. |
| Structural magnetic resonance imaging predictors of responsiveness to cognitive behaviour therapy in psychosis | 2009 | Premkumar et al. |
| White matter correlates of cognitive inhibition during development: A diffusion tensor imaging study | 2014 | Treit et al. |
| Adaptive Adolescent Flexibility: Neurodevelopment of Decision-making and Learning in a Risky Context | 2017 | McCormick and Telzer |
| Brain effects of computer-assisted cognitive remediation therapy in anorexia nervosa: A pilot fMRI study | 2016 | Brockmeyer et al. |
| Shifting set about task switching: Behavioral and neural evidence for distinct forms of cognitive flexibility | 2008 | Ravizza and Carter |
| Neural Components Underlying Behavioral Flexibility in Human Reversal Learning | 2010 | Ghahremani et al. |
| Task-switching Cost and Intrinsic Functional Connectivity in the Human Brain: Toward Understanding Individual Differences in Cognitive Flexibility | 2015 | Yin et al. |
| Speed-accuracy strategy regulations in prefrontal tumor patients | 2016 | Campanella et al. |
| Neural circuitry underlying affective response to peer feedback in adolescence | 2012 | Guyer et al. |
| Striatal dopamine influences the default mode network to affect shifting between object features | 2012 | Dang et al. |
| Examining dorsal striatum in cognitive effort using Parkinson's disease and fMRI | 2014 | MacDonald et al. |

The list is sorted by the order of contributing publications to the term.

**Reference**

Ben-Soussan, T. D., Berkovich-Ohana, A., Piervincenzi, C., Glicksohn, J., and Carducci, F. (2015). Embodied cognitive flexibility and neuroplasticity following Quadrato Motor Training. *Frontiers in Psychology* 6, 1021. doi: 10.3389/fpsyg.2015.01021.

Braem, S., King, J. A., Korb, F. M., Krebs, R. M., Notebaert, W., and Egner, T. (2013). Affective modulation of cognitive control is determined by performance-contingency and mediated by ventromedial prefrontal and cingulate cortex. *Journal of Neuroscience* 33, 16961–16970. doi: 10.1523/JNEUROSCI.1208-13.2013.

Brockmeyer, T., Walther, S., Ingenerf, K., Wild, B., Hartmann, M., Weisbrod, M., et al. (2016). Brain effects of computer-assisted cognitive remediation therapy in anorexia nervosa: a pilot fMRI study. *Psychiatry Research: Neuroimaging* 249, 52–56. doi: 10.1016/j.pscychresns.2016.02.007.

Buchweitz, A., and Prat, C. (2013). The bilingual brain: Flexibility and control in the human cortex. *Physics of life reviews* 10, 428–443. doi: 10.1016/j.plrev.2013.07.020.

Calcott, R. D., and Berkman, E. T. (2015). Neural correlates of attentional flexibility during approach and avoidance motivation. *PloS One* 10, e0127203. doi: 10.1371/journal.pone.0127203.

Campanella, F., Skrap, M., and Vallesi, A. (2016). Speed-accuracy strategy regulations in prefrontal tumor patients. *Neuropsychologia* 82, 1–10. doi: 10.1016/j.neuropsychologia.2016.01.008.

Chen, Q., Yang, W., Li, W., Wei, D., Li, H., Lei, Q., et al. (2014). Association of creative achievement with cognitive flexibility by a combined voxel-based morphometry and resting-state functional connectivity study. *Neuroimage* 102, 474–483. doi: 10.1016/j.neuroimage.2014.08.008.

Dang, L. C., Donde, A., Madison, C., O’Neil, J. P., and Jagust, W. J. (2012). Striatal dopamine influences the default mode network to affect shifting between object features. *Journal of cognitive neuroscience* 24, 1960–1970. doi: 10.1162/jocn_a_00252.

Dong, G., Lin, X., Zhou, H., and Lu, Q. (2014). Cognitive flexibility in internet addicts: fMRI evidence from difficult-to-easy and easy-to-difficult switching situations. *Addictive Behaviors* 39, 677–683. doi: /10.1016/j.addbeh.2013.11.028.

Ezekiel, F., Bosma, R., and Morton, J. B. (2013). Dimensional change card sort performance associated with age-related differences in functional connectivity of lateral prefrontal cortex. *Developmental cognitive neuroscience* 5, 40–50. doi: /10.1016/j.dcn.2012.12.001.

Ghahremani, D. G., Monterosso, J., Jentsch, J. D., Bilder, R. M., and Poldrack, R. A. (2010). Neural components underlying behavioral flexibility in human reversal learning. *Cerebral cortex* 20, 1843–1852. doi: /10.1093/cercor/bhp247.

Guyer, A. E., Choate, V. R., Pine, D. S., and Nelson, E. E. (2012). Neural circuitry underlying affective response to peer feedback in adolescence. *Social cognitive and affective neuroscience* 7, 81–92. doi: /10.1093/scan/nsr043.

Hauser, T. U., Iannaccone, R., Walitza, S., Brandeis, D., and Brem, S. (2015). Cognitive flexibility in adolescence: neural and behavioral mechanisms of reward prediction error processing in adaptive decision making during development. *Neuroimage* 104, 347–354. doi: /10.1016/j.neuroimage.2014.09.018.

Kim, P., Jenkins, S. E., Connolly, M. E., Deveney, C. M., Fromm, S. J., Brotman, M. A., et al. (2012). Neural correlates of cognitive flexibility in children at risk for bipolar disorder. *Journal of psychiatric research* 46, 22–30. doi: /10.1016/j.jpsychires.2011.09.015.

Lao-Kaim, N. P., Fonville, L., Giampietro, V. P., Williams, S. C., Simmons, A., and Tchanturia, K. (2015). Aberrant function of learning and cognitive control networks underlie inefficient cognitive flexibility in anorexia nervosa: a cross-sectional fMRI study. *PloS one* 10, e0124027. doi: /10.1371/journal.pone.0124027.

Leber, A. B., Turk-Browne, N. B., and Chun, M. M. (2008). Neural predictors of moment-to-moment fluctuations in cognitive flexibility. *Proceedings of the National Academy of Sciences* 105, 13592–13597. doi: /10.1073/pnas.0805423105.

MacDonald, A. A., Seergobin, K. N., Tamjeedi, R., Owen, A. M., Provost, J.-S., Monchi, O., et al. (2014). Examining dorsal striatum in cognitive effort using Parkinson’s disease and fMRI. *Annals of clinical and translational neurology* 1, 390–400. doi: /10.1002/acn3.62.

McCormick, E. M., and Telzer, E. H. (2017). Adaptive adolescent flexibility: neurodevelopment of decision-making and learning in a risky context. *Journal of cognitive neuroscience* 29, 413–423. doi: /10.1162/jocn_a_01061.

Premkumar, P., Fannon, D., Kuipers, E., Peters, E. R., Anilkumar, A. P., Simmons, A., et al. (2009). Structural magnetic resonance imaging predictors of responsiveness to cognitive behaviour therapy in psychosis. *Schizophrenia Research* 115, 146–155. doi: /10.1016/j.schres.2009.08.007.

Ravizza, S. M., and Carter, C. S. (2008). Shifting set about task switching: Behavioral and neural evidence for distinct forms of cognitive flexibility. *Neuropsychologia* 46, 2924–2935. doi: /10.1016/j.neuropsychologia.2008.06.006.

Roberts, R. P., Wiebels, K., Sumner, R. L., van Mulukom, V., Grady, C. L., Schacter, D. L., et al. (2017). An fMRI investigation of the relationship between future imagination and cognitive flexibility. *Neuropsychologia* 95, 156–172. doi: /10.1016/j.neuropsychologia.2016.11.019.

Sato, Y., Saito, N., Utsumi, A., Aizawa, E., Shoji, T., Izumiyama, M., et al. (2013). Neural basis of impaired cognitive flexibility in patients with anorexia nervosa. *PloS one* 8, e61108. doi: /10.1371/journal.pone.0061108.

Sekutowicz, M., Schmack, K., Steimke, R., Paschke, L., Sterzer, P., Walter, H., et al. (2016). Striatal activation as a neural link between cognitive and perceptual flexibility. *NeuroImage* 141, 393–398. doi: /10.1016/j.neuroimage.2016.07.046.

Stelzel, C., Fiebach, C. J., Cools, R., Tafazoli, S., and D’Esposito, M. (2013). Dissociable fronto-striatal effects of dopamine D2 receptor stimulation on cognitive versus motor flexibility. *Cortex* 49, 2799–2811. doi: /10.1016/j.cortex.2013.04.002.

Treit, S., Chen, Z., Rasmussen, C., and Beaulieu, C. (2014). White matter correlates of cognitive inhibition during development: a diffusion tensor imaging study. *Neuroscience* 276, 87–97. doi: /10.1016/j.neuroscience.2013.12.019.

Ueltzhöffer, K., Armbruster-Genç, D. J., and Fiebach, C. J. (2015). Stochastic dynamics underlying cognitive stability and flexibility. *PLoS computational biology* 11, e1004331. doi: /10.1371/journal.pcbi.1004331.

Van Schouwenburg, M. R., Onnink, A. M. H., Ter Huurne, N., Kan, C. C., Zwiers, M. P., Hoogman, M., et al. (2014). Cognitive flexibility depends on white matter microstructure of the basal ganglia. *Neuropsychologia* 53, 171–177.

Vriend, C., de Wit, S. J., Remijnse, P. L., van Balkom, A. J., Veltman, D. J., and van den Heuvel, O. A. (2013). Switch the itch: a naturalistic follow-up study on the neural correlates of cognitive flexibility in obsessive-compulsive disorder. Psychiatry Research: *Neuroimaging* 213, 31–38. doi: /10.1016/j.pscychresns.2012.12.006.

Weathers, J., Brotman, M. A., Deveney, C. M., Kim, P., Zarate Jr, C., Fromm, S., et al. (2013). A developmental study on the neural circuitry mediating response flexibility in bipolar disorder. Psychiatry Research: *Neuroimaging* 214, 56–65. doi: /10.1016/j.pscychresns.2013.05.002.

Yin, S., Wang, T., Pan, W., Liu, Y., and Chen, A. (2015). Task-switching cost and intrinsic functional connectivity in the human brain: Toward understanding individual differences in cognitive flexibility. *PloS one* 10, e0145826. doi: /10.1371/journal.pone.0145826.

# Supplementary Figures

**Figure S1.** **Decoding analysis using NeuroQuery platform**


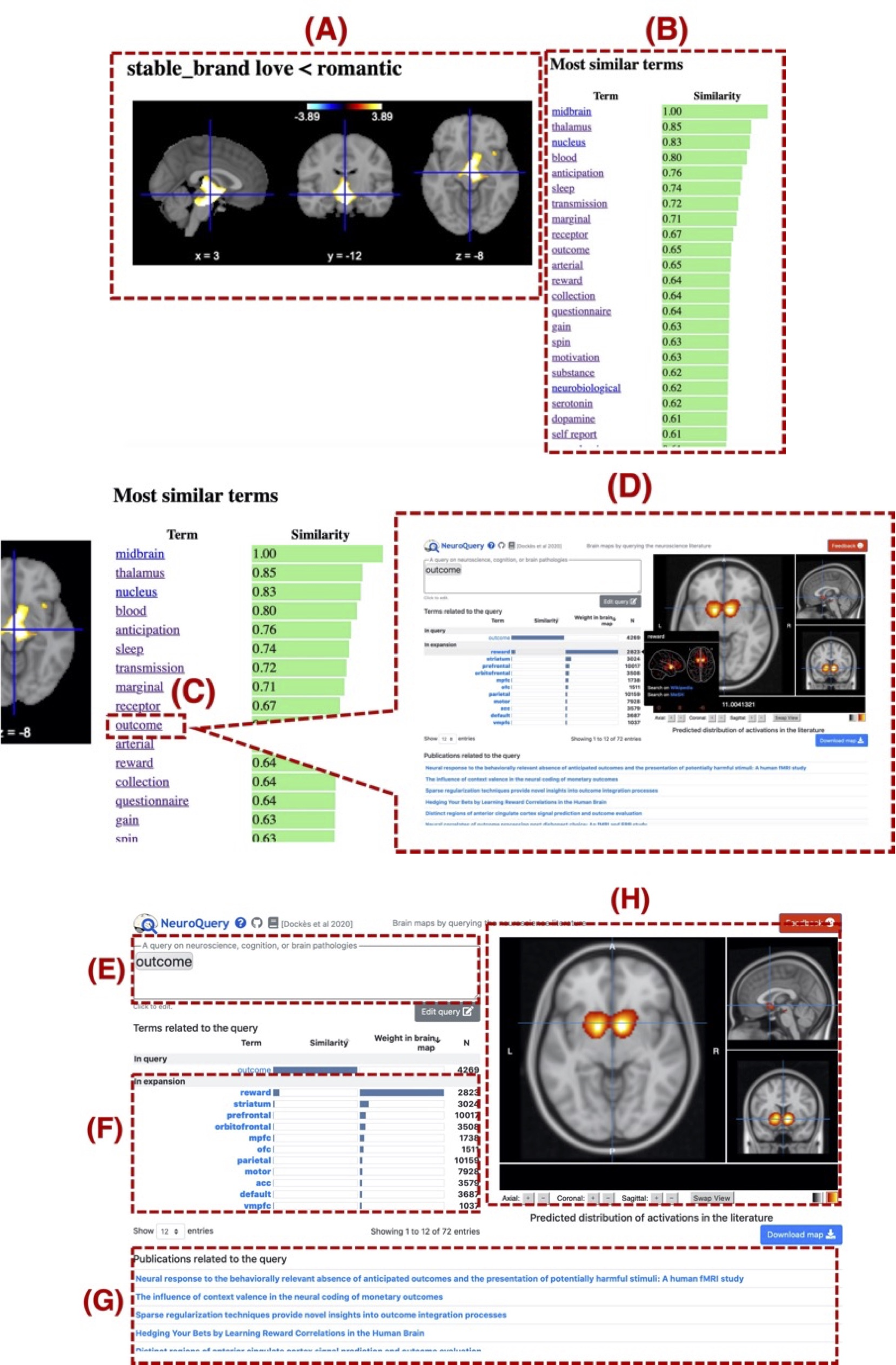


**Step 1: (A) and (B) are displayed by coding with Python API**, (A) Displays activated brain regions**,** (B) Decoded terms related to the activated brain regions / Similarity score **(**If a term is interpretable, this step is a terminal step. If a term is uninterpretable, continue to step 2). **Step 2: Infer and interpret the uninterpretable terms,** (C) Select an uninterpretable term**,** (D) Results queried by an uninterpretable term, (E) Query field (Filled in an interpretable and interesting term), (F) In expansion field (Terms related to an interpretable and interesting term), (G) Publication field **(**Display publications related to the query)**,** (H) Brain regions related to the query **(**Predicted by foci listed in related publications).
